# Effects of Smoking and Cessation on Subclinical Arterial Disease: A Substudy of a Randomized Controlled Trial

**DOI:** 10.1371/journal.pone.0035332

**Published:** 2012-04-09

**Authors:** Heather M. Johnson, Megan E. Piper, Timothy B. Baker, Michael C. Fiore, James H. Stein

**Affiliations:** Department of Medicine, University of Wisconsin School of Medicine and Public Health, Madison, Wisconsin, United States of America; Virginia Commonwealth University, United States of America

## Abstract

**Background:**

The mechanisms by which smoking cessation reduces cardiovascular disease risk are unclear. We evaluated longitudinal changes in carotid intima-media thickness among current smokers enrolled in a prospective, randomized smoking cessation clinical trial.

**Methodology/Principal Findings:**

Subjects were enrolled in a randomized, double-blind, placebo-controlled trial of 5 smoking cessation pharmacotherapies and underwent carotid ultrasonography with carotid intima-media thickness measurement. Subjects were classified as continuously abstinent (biochemically confirmed abstinence at 6 months, 1 year, and 3 years post-quit attempt), intermittently abstinent (reported smoking at one of the three time points), or smoked continuously (reported smoking at all three time points). The primary endpoint was the absolute change (mm) in carotid intima-media thickness (ΔCIMT_max_) before randomization and 3 years after the target quit date. Pearson correlations were calculated and multivariable regression models (controlling for baseline CIMT_max_ and research site) were analyzed. Among 795 subjects (45.2±10.6 years old, 58.5% female), 189 (23.8%) were continuously abstinent, 373 (46.9%) smoked continuously, and 233 (29.3%) were abstinent intermittently. There was a greater increase in carotid intima-media thickness among subjects who were continuously abstinent than among those who smoked continuously (p = 0.020), but not intermittently (p = 0.310). Antihypertensive medication use (p = 0.001) and research site (p<0.001) independently predicted ΔCIMTmax – not smoking status. The greatest increase in carotid intima-media thickness among continuous abstainers was related to increases in body-mass index (p = 0.043).

**Conclusions/Significance:**

Smoking status did not independently predict ΔCIMT_max_; increasing body-mass index and antihypertensive medication use were the most important independent predictors. The rapid reduction in cardiovascular disease events observed with smoking cessation is unlikely to be mediated by changes in subclinical atherosclerosis burden.

**Trial Registration:**

ClinicalTrials.gov NCT00332644

## Introduction

It has been hypothesized that the strong relationship between cigarette smoking and the development of cardiovascular disease (CVD) is mediated by inflammatory pathways, lipid oxidation, and vascular dysfunction [1], [2]. Smoking cessation reduces cardiovascular disease risk; however, the mechanisms and time-course of the effects of cessation on CVD risk are unknown [3]–[5]. In some studies cardiovascular disease risk begins to decrease within 3 years of smoking cessation [3], [4]. Carotid intima-media thickness (CIMT) is a measure of subclinical arterial injury that predicts future cardiovascular disease events. In cross-sectional analyses, we and others previously demonstrated that smoking intensity (pack-years) was associated with increased CIMT [6], [7]. The purpose of this paper was to describe longitudinal 3-year changes in CIMT observed in a large, modern cohort of current smokers enrolled in a prospective, randomized clinical trial of smoking cessation pharmacotherapies and to determine if smoking cessation is associated with a reduction in progression in CIMT.

## Methods

The protocol for this trial and support CONSORT checklist are available as supporting information; see Checklist S1 and Protocol S1. Also included as supporting information is a manuscript by Piper, et al.; see Piper S1.

### Study Procedures

#### Ethics Statement

This study was approved by the institutional review board of the University of Wisconsin School of Medicine and Public Health. It was conducted according to the principles expressed in the Declaration of Helsinki. All participants provided written informed consent.

Subjects were enrolled in a randomized, double-blinded, placebo-controlled comparative effectiveness trial of smoking cessation pharmacotherapies that also intended to study the natural history of smoking and cessation on subclinical atherosclerosis [6], [8]. Each participant’s consent for the randomized clinical trial included permission to evaluate the physiologic effects of active cigarette use and smoking cessation on atherosclerosis by ultrasonographic evaluation of CIMT at baseline and after 3 years. At the baseline and year 3 visits, anthropometric data, fasting laboratory tests, validated questionnaires, and interviews were completed. Participants were randomized to one of six treatment conditions: nicotine lozenge, nicotine patch, sustained-release bupropion, nicotine patch plus nicotine lozenge, sustained-release bupropion plus nicotine lozenge, or placebo. Inclusion and exclusion criteria were based on participation in the clinical trial. Inclusion criteria were age ≥18 years, current smoking of ≥10 cigarettes?day for the previous 6 months, an expired carbon monoxide level of >9 ppm, and stated motivation to try to quit smoking. Major exclusion criteria were uncontrolled hypertension (blood pressure >160?100 mmHg) and myocardial infarction in the previous 4 weeks. Additional study details, including the flowchart of participants, have been reported previously [6], [8].

### Carotid Ultrasonography

**Figure 1:**
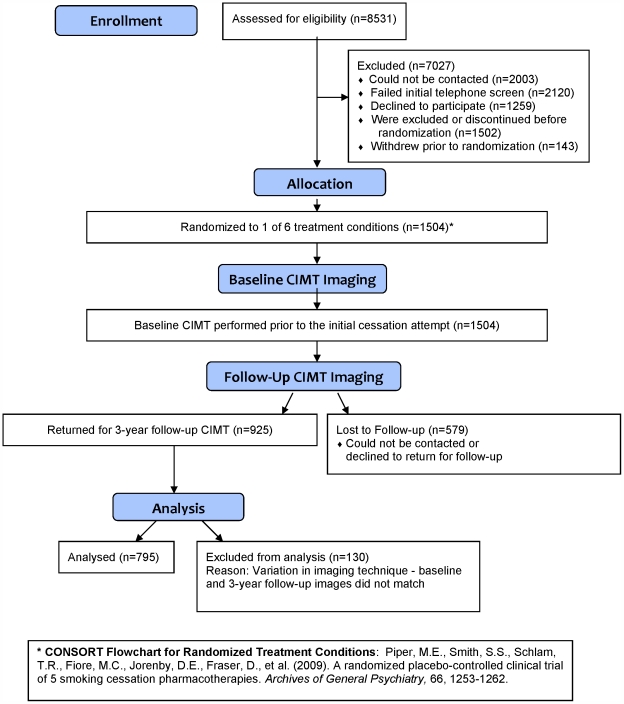
CONSORT: Screening, Randomization, Baseline Imaging and Follow-up.

Subjects underwent carotid ultrasonography with CIMT measurement prior to randomization and 3 years after the target quit date at two research sites in Milwaukee and Madison, Wisconsin [6], [8]. Maximum right- and/or left-sided far wall common carotid artery CIMT measurements were averaged. The far wall CIMT of the distal 1 cm of each common carotid artery was measured in triplicate at the electrocardiographic R-wave using a high-resolution linear array transducer (L10–5) and cardiovascular ultrasound system (CV70; Siemens Medical Solutions, Mountain View, WA) [6]. The CIMT imaging protocol has been described previously [9]. Images were transferred via the Internet to a secure Web server at the University of Wisconsin Atherosclerosis Imaging Program, the core ultrasound laboratory. All scanners were trained and certified by the core lab. A single reader blindly measured all baseline and follow-up images using a semi-automated border detection tool [8]. At the time of CIMT image analysis, approximately 130 participants were excluded because the year 3 CIMT scan images did not match the baseline images closely enough for comparative analysis (Figure 1). This was a predefined exclusion criterion. The primary endpoint was the absolute change (mm) in CIMT (ΔCIMT_max_) between baseline and year 3. The coefficient of variation for repeatability of CIMT_max_ measurements was 3.46%. Subjects were classified as continuously abstinent (biochemically confirmed abstinence at 6 months, 1 year, and 3 years post-quit attempt), intermittently abstinent (reported smoking at one of the three time points), or smoked continuously (reported smoking at all three time points).

### Statistical Analysis

Analyses were performed with SPSS software (SPSS, Inc., Chicago, IL). Our sample size estimate was based on the effect size of abstinence from smoking (compared to continued smoking) reported in statistical models from the Atherosclerosis Risk in Communities Study that adjusted for CVD risk factors and environmental tobacco smoke [7]. Our target sample size was 750 participants (300 abstainers and 450 smokers), based on a conservative 5–10% attrition rate from baseline to Year 3. We hypothesized that the ΔCIMT_max_ of continuing smokers would progress at a rate of approximately 0.043 mm/3 years and former smokers would progress at a rate of approximately 0.035 mm/3 years for an absolute difference of 0.007–0.008 mm/3 years, a relative decrease (treatment effect) of 17%. We used a two-tailed α = 0.05 to determine that we had 81% power to detect a 20% difference in progression between abstinent and smoking participants.

CVD risk factors and CIMT were described by means ± standard deviations, unless noted otherwise. Pearson correlations were estimated between ΔCIMT_max_ and current age, separately for the baseline values and the 3-year change values for the following continuous variables: body mass index (BMI), waist circumference, carbon monoxide levels, current pack-years smoking, heart rate, systolic blood pressure, diastolic blood pressure, total cholesterol, triglycerides, low-density lipoprotein cholesterol (LDL), high-density lipoprotein (HDL) cholesterol, fasting glucose, hemoglobin A_1_C, high sensitivity C-reactive protein, average number of alcohol-containing beverages per month, and mean CIMT. Pearson correlations also were estimated between ΔCIMT_max_ and baseline Fägerstrom Test for Nicotine Dependence score (total). A one-way ANOVA was performed between ΔCIMT_max_ and smoking status groups and smoking cessation treatment arms (placebo, bupropion, lozenge, patch, bupropion + lozenge, patch + lozenge). T-tests were performed to compare the change in ΔCIMT_max_ between genders, races (white vs. non-white), research sites (Madison vs. Milwaukee, WI), and subjects who did and did not use lipid-lowering medications (yes/no) or anti-hypertensive medications (yes/no). The best-fitting multivariable linear regression models were developed using a backward model-building approach (Hosmer & Lemeshow) for ΔCIMT_max_. All variables with p<0.20 in individual analyses were added to the model. Predictors were then systematically removed until only statistically significant (p<0.05) predictors remained, controlling for baseline CIMT_max_ and research site.

## Results

The 795 subjects (45.2±10.6 years old, 58.5% female) who had CIMT scans at baseline and year 3 are described in Table 1. There were 189 (23.8%) subjects who were continuously abstinent, 373 (46.9%) smoked continuously, and 233 (29.3%) were abstinent intermittently. Abstinence rates did not differ by gender (p = 0.410).

**Table 1:** Subject Characteristics at Baseline and 3 Years after the Target Quit Date*.

	All subjects		Continuously Smoking				Intermittently Smoking				Continuously Abstinent				P values for one-way ANOVA	
	Baseline		Baseline Mean (SD)	3 Year Mean (SD)	Delta = Year 3-Baseline (SD)	Baseline Mean (SD)		3 Year Mean (SD)	Delta (SD)		Baseline Mean (SD)	3 Year Mean (SD)	Delta (SD)	Baseline		Delta (Year 3-Baseline)
N	795		373			233					189					
Age	45.3 (10.6)		45.1 (9.8)			44.5 (11.3)					46.6 (11.3)			0.101		
Body mass index (kg/m^2^)	28.7 (6.1)		29.0 (6.4)	29.2 (6.6)	0.3a,c (3.2)	28.4 (5.8)		29.5 (6.5)	1.1 b,c (3.0)		28.6 (5.8)	30.7 (6.9)	2.2 a,b (4.0)	0.522		<0.001
Waist circumference (cm)	95.3 (15.67)		95.3 (15.7)	100.0 (34.2)	4.3 (33.1)	94.8 (16.0)		98.2 (16.5)	3.2 (12.2)		100.0 (15.0)	101.2 (16.1)	5.0 (8.7)	0.753		0.725
Carbon monoxide (ppm)	25.6 (12.1)		26.9 (12.1)	19.8 (10.9)	-7.0 a,c (12.2)	24.72 (12.9)		8.8 (9.9)	-16.1 b,c (14.4)		24.3 (11.0)	1.6 (1.3)	-22.6a,b (11.1)	0.026		<0.001
Current Pack-years	29.3 (20.0)		30.8 (19.4)	32.5 (19.1)	1.9a,c (1.1)	27.9 (21.6)		28.8 (21.9)	0.7 b,c (1.1)		28.1 (19.2)	28.1 (19.2)	0.0a,b (0.0)	0.139		<0.001
Heart rate (beats/minute)	73.5 (9.4)		73.8 (9.6)	74.3 (33.1)	0.5 (34.0)	73.4 (9.2)		69.4 (9.1)	-3.6 (12.0)		73.1 (9.5)	71.5 (24.3)	-1.8 (25.3)	0.721		0.180
Systolic blood pressure (mmHg)	119.2 (14.6)		119.2 (15.0)	117.5 (17.5)	-1.8 (15.7)	117.8 (13.4)		114.3 (14.9)	-3.7 (15.3)		120.9 (15.2)	116.6 (15.6)	-4.2 (15.2)	0.093		0.150
Diastolic blood pressure (mmHg)	74.4 (10.1)		74.1 (10.6)	75.3 (33.0)	1.2 (33.6)	74.0 (9.7)		75.1 (41.7)	1.3 (42.6)		75.7 (9.3)	76.9 (43.8)	1.4 (44.6)	0.154		0.998
Total cholesterol (mg/dL)	185.2 (35.2)		186.1 (36.5)	192.0 (38.5)	5.6 (29.0)	183.5 (35.2)		189.2 (37.5)	7.7 (29.4)		185.7 (32.5)	189.4 (37.3)	4.7 (31.4)	0.662		0.571
Low-density lipoprotein cholesterol (mg/dL)	118.7 (90.0)		118.9 (30.9)	125.3 (33.5)	6.0 c (26.1)	116.8 (29.7)		121.6 (31.9)	6.3 (25.1)		120.7 (28.4)	120.2 (30.7)	0.2 b (25.9)	0.424		0.031
High-density lipoprotein cholesterol (mg/dL)	42.7 (13.6)		42.4 (13.6)	44.6 (15.4)	2.0 c (9.6)	43.5 (13.8)		46.4 (14.5)	2.9 c (9.7)		42.3 (13.4)	48.3 (15.4)	5.5 b (9.9)	0.598		0.001
Triglycerides (mg/dL)	147.9 (106.4)		155.8 (123.7)	145.0 (107.2)	-8.5 (99.5)	143.1 (92.7)		135.0 (86.2)	-5.2 (77.7)		138.6 (81.9)	135.5 (75.5)	-0.3 (76.4)	0.148		0.608
Fasting glucose (mg/dL)	94.2 (15.2)		94.7 (17.3)	95.1 (19.4)	0.5 (17.5)	94.0 (14.2)		99.8 (62.2)	6.6 (62.1)		93.6 (11.5)	100.0 (22.8)	6.1 (18.1)	0.702		0.116
Hemoglobin A_1_C (%)	5.5 (0.5)		5.5 (0.4)	5.7 (0.6)	0.1 (0.5)	5.5 (0.5)		5.6 (0.4)	0.1 (0.3)		5.5 (0.5)	5.8 (0.6)	0.2 (0.4)	0.445		0.196
High-sensitivity C-reactive protein (mg/L)	1.6 (2.7)		1.7 (2.9)	4.3 (9.5)	2.7 (9.6)	1.4 (2.5)		3.7 (4.7)	2.4 (4.4)		1.6 (2.6)	4.3 (5.7)	2.7 (5.8)	0.527		0.894
Alcohol use (drinks/month)	18.3 (35.4)		18.4 (44.1)	18.2 (34.1)	2.3 (29.5)	15.9 (22.3)		15.5 (26.2)	-1.2 (22.4)		21.3 (29.5)	20.9 (29.9)	-1.2 (22.8)	0.329		0.226
Maximum CIMT	0.88 (0.14)		0.88 (0.13)	0.88 (0.16)	-0.002 c (0.084)	0.86 (0.14)		0.87 (0.16)	0.007 (0.082)		0.89 (0.17)	0.91 (0.20)	0.019^b^ (0.091)	0.100		0.021

* all values are means (standard deviation); ANOVA  =  analysis of variance, CIMT  =  carotid intima-media thickness

a =  significantly different from intermittent smokers (p<0.05), based on a post-hoc Tukey test

b =  significantly different from continuous smokers (p<0.05), based on a post-hoc Tukey test

c =  significantly different from continuously abstinent (p<0.05), based on a post-hoc Tukey test

Age was significantly correlated with baseline cigarettes smoked/day (r = 0.17, p<0.001) but was not correlated with ΔCIMT_max_ (r = 0.04, p = 0.321). ΔCIMT_max_ was correlated weakly, but significantly, with 3-year changes in LDL cholesterol (r = -0.10, p = 0.007), total cholesterol (r = -0.09, p = 0.020), hemoglobin A_1_C (r = 0.08, p = 0.030), and use of antihypertensive medications at either visit (r = 0.11, p = 0.002). ΔCIMT_max_ did not differ between smoking cessation treatment arms (p = 0.993), by sex (p = 0.146), race (p = 0.338), or age (p = 0.321). There was a greater increase in CIMT among subjects at the Milwaukee site (p<0.001) and among subjects who were continuously abstinent compared to those who smoked continuously (p = 0.020) but not intermittently (p = 0.310).

Some CIMT predictors were significantly different between the Madison and Milwaukee research sites. Important demographic differences between sites were gender (p = 0.048), age (p = 0.003), race (p<0.001), marital status (p = 0.029), education (p<0.001), and Fägerstrom Test for Nicotine Dependence score (p = 0.021). Abstinence rate (p<0.001), antihypertensive medication use (p = 0.036), and 3-year changes in systolic blood pressure (p = 0.048), HgbA1C (p<0.001), and LDL cholesterol (p = 0.001) differed between sites.

ANOVA tests with post-hoc analysis were performed to compare 3-year changes in CIMT predictors to the three smoking classifications. Continuously abstinent participants smoked fewer cigarettes/day at baseline (p = 0.023) and by year 3 had a greater increase in HDL cholesterol (p = 0.001), less increase in LDL cholesterol (p = 0.031), and greater increase in glucose (p = 0.036) than both those who smoked continuously and those who were intermittently abstinent. Those who were intermittently abstinent had greater increases in body-mass index by year 3 than those who smoked continuously (p = 0.030), but not those who were continuously abstinent (p = 0.292).

Using all biological, demographic, site, and cessation parameters as candidate variables, and controlling for baseline CIMT_max_ and site, the only independent associations of ΔCIMT_max_ in the best-fitting linear regression model were anti-hypertensive medication use (standardized β = 0.15, p = 0.001) and site (β = 0.31, p<0.001). At the Milwaukee site, use of antihypertensive medications (p = 0.009) was the only variable independently associated with ΔCIMT_max_; at the Madison site, male sex (p = 0.015) was the only variable independently associated with ΔCIMT_max_. In the entire subject population, neither baseline smoking burden, baseline smoking intensity, nor abstinence status were independently associated with ΔCIMT_max_. Change in BMI (p = 0.031), antihypertensive medication use (p = 0.019), and continuous abstinence (p = 0.040) were weakly but independently associated with being in the highest quartile of ΔCIMT_max_. These effect sizes were modest (R^2^_adj_ = 0.032).

Based upon these findings we conducted exploratory mediation analysis using the conservative Baron and Kenny [10] approach and the joint significance test to examine how the direct path from smoking status to ΔCIMT_max_ was affected by the inclusion of the change in BMI over 3 years. Linear regression analyses were performed among continuously abstinent subjects whose ΔCIMT_max_ was in the top or bottom quartile. There were significant effects of smoking status on the change in BMI (β  =  0.225, p <0.001) and significant effects of the change in BMI on ΔCIMT_max_ (β  =  0.124, p  =  0.015). Both of the indirect paths were sufficient to conclude mediation using the joint significance test [11]. Subsequently, we assessed the direct effect of smoking status on ΔCIMT_max_ when change in BMI was added to the model and the direct effect changed from β  =  0.126 (p  =  0.012) to beta  = 0.102 (p  =  0.048) suggestive of mediation.

## Discussion

After smoking cessation, CVD risk decreases within 3 years [3], [4]. We hypothesized that CIMT progression would be reduced after 3 years of smoking cessation, but it was not. Male sex, increasing BMI, and antihypertensive medication use were the most important independent predictors of ΔCIMT_max_. We also observed a powerful site effect that could not be explained by any patient or technical factors evaluated. Smoking status did not independently predict ΔCIMT_max_; indeed, individuals who were continuously abstinent had a nominally greater increase in CIMT. In this same population we demonstrated previously that endothelial function improved [12] and HDL cholesterol, total HDL, and large HDL particles increased [13], within the first year after smoking cessation, *despite weight gain*. Therefore, the early decrease in CVD risk observed after smoking cessation may be due to improved endothelial function, CVD risk factors, and perhaps unmeasured factors related to hemostasis and thrombosis.

The longitudinal effects of smoking cessation on subclinical arterial disease are less clear. Such effects may require more than 3 years to emerge or might have been masked by BMI increases among abstainers. In this same cohort we previously described an increase in waist circumference and lack of a significant reduction in high-sensitivity C-reactive protein levels after 1 year of cessation [12], [14], both of which may influence short-term changes in CIMT. A previous study suggested that C-reactive protein levels require approximately 5 years to return to baseline after smoking cessation, even after controlling for other cardiovascular disease risk factors [15]. Both short- and long-term decreases in CVD risk after smoking cessation are well-established [3]–[5], and the beneficial effects of smoking cessation clearly outweigh any adverse metabolic effects. Thus, the decrease in CVD morbidity and mortality after cessation is likely due to multiple mechanistic pathways with differing temporal responses to smoking cessation. This study highlights the limitations of CIMT as a surrogate marker for short-term CVD risk reduction after smoking cessation due to the complex vascular pathophysiology associated with cessation.

Limitations of this study include between-site differences and a relatively small sample size of continuous abstainers. In addition, the lack of longitudinal carotid plaque assessment limits further evaluation of confounding factors associated with subclinical atherosclerosis and smoking cessation. It also is possible that changes in the internal carotid or bulb CIMT may have been different than we observed in the common carotid artery. We did not assess the incidence of CVD events during this short-term follow-up. Our 3-year drop-out rate (38.2%) is consistent with previous smoking cessation trials [12]. A larger prospective clinical trial with longer follow-up and combined CIMT and plaque assessment is needed to better understand the effects of smoking cessation on subclinical arterial disease.

## Supporting Information

Checklist S1 **CONSORT Checklist.**
(DOC)

Piper S1 **A randomized placebo-controlled clinical trial of five smoking cessation pharmacotherapies.**
(PDF)

Protocol S1 **Trial Protocol.**
(DOC)
