# Supplementary material for: Effects of Smoking and Cessation on Subclinical Arterial Disease: A Substudy of a Randomized Controlled Trial
Source: PLoS One. 2012 Apr 9;7(4):e35332. doi: 10.1371/journal.pone.0035332 (PMC3322167; doi:10.1371/journal.pone.0035332)
Supplement: Piper S1 — A randomized placebo-controlled clinical trial of five smoking cessation pharmacotherapies. (PDF) [file pone.0035332.s002.pdf]

NIH-PA Author Manuscript
NIH-PA Author Manuscript
NIH-PA Author Manuscript

NIH Public Access
Author Manuscript
Arch Gen Psychiatry. Author manuscript; available in PMC 2010 November 1.

Published in final edited form as:
Arch Gen Psychiatry. 2009 November ; 66(11): 1253–1262. doi:10.1001/archgenpsychiatry.2009.142.

A randomized placebo-controlled clinical trial of five smoking cessation pharmacotherapies

Megan E. Piper, Ph.D.,
Center for Tobacco Research and Intervention, University of Wisconsin School of Medicine and Public Health, Madison, WI

Stevens S. Smith, Ph.D.,
Center for Tobacco Research and Intervention and Department of Medicine, University of Wisconsin School of Medicine and Public Health, Madison, WI

Tanya R. Schlam, Ph.D.,
Center for Tobacco Research and Intervention, University of Wisconsin School of Medicine and Public Health, Madison, WI

Michael C. Fiore, M.D., M.P.H.,
Center for Tobacco Research and Intervention and Department of Medicine, University of Wisconsin School of Medicine and Public Health, Madison, WI

Douglas E. Jorenby, Ph.D.,
Center for Tobacco Research and Intervention and Department of Medicine, University of Wisconsin School of Medicine and Public Health, Madison, WI

David Fraser, M.S., and
Center for Tobacco Research and Intervention, University of Wisconsin School of Medicine and Public Health, Madison, WI

Timothy B. Baker, Ph.D.
Center for Tobacco Research and Intervention and Department of Medicine, University of Wisconsin School of Medicine and Public Health, Madison, WI

Abstract

Context—Little direct evidence exists on the relative efficacies of different smoking cessation pharmacotherapies, yet such evidence is needed to make informed decisions about their clinical use.

Objective—The primary objective of this research was to assess the relative efficacies of five smoking cessation pharmacotherapy interventions using placebo-controlled, head-to-head comparisons.

Design—This was a randomized double-blind, placebo-controlled clinical trial.

Setting—Smokers were recruited from the community at two urban research sites.

Patients—Participants were 1504 adult smokers who smoked at least 10 cigarettes per day during the past 6 months and reported being motivated to quit smoking. Participants were excluded if they reported: using any form of tobacco other than cigarettes; current use of bupropion; having a current

Corresponding Author: Megan E. Piper, Ph.D., Center for Tobacco Research and Intervention, 1930 Monroe St., Suite 200, Madison, WI, 53711. Telephone: (608) 265-5472. Fax: (608) 265-3102. mep@ctri.medicine.wisc.edu.

The authors report the following potential conflicts of interest for the last 5 years. Megan E. Piper, Tanya R. Schlam and David Fraser have no potential conflicts of interest to disclose.

NIH-PA Author Manuscript

NIH-PA Author Manuscript

NIH-PA Author Manuscript

psychosis or schizophrenia diagnosis; or having medical contraindications for any of the study medications.

Interventions—Participants were randomized to one of six treatment conditions: nicotine lozenge, nicotine patch, bupropion SR, nicotine patch + nicotine lozenge, bupropion + nicotine lozenge or placebo. In addition, all participants received six individual counseling sessions.

Main Outcome Measures—The main outcome measures were biochemically-confirmed 7-day point-prevalence abstinence assessed at 1 week post-quit, end of treatment (8 weeks post-quit) and 6 months post-quit. Other outcomes were initial cessation, number of days to lapse, number of days to relapse, and latency to relapse after the first lapse.

Results—All pharmacotherapies differed from placebo when examined without protection for multiple comparisons (OR's = 1.63–2.34). With such protection, only the nicotine patch + nicotine lozenge (OR = 2.34, p < .001) produced significantly higher abstinence rates at 6-months post-quit than did placebo.

Conclusions—While the nicotine lozenge, bupropion, and bupropion + lozenge produced effects that were comparable to those reported in previous research, the nicotine patch + lozenge produced the greatest benefit relative to placebo for smoking cessation.

Introduction

Many smokers have successfully quit using a variety of smoking cessation pharmacotherapies. Yet there is little direct evidence on the relative efficacies of these different pharmacotherapies, and without such evidence clinicians and smokers lack a strong empirical basis for recommending or selecting from amongst them. The current paper reports data on the efficacies of five different smoking cessation pharmacotherapies. This research also evaluates the efficacy of the nicotine lozenge, providing additional information on a treatment that has been supported by only one placebo controlled study published to date. Finally, this research evaluates two different medication combinations, one of which (i.e., the nicotine patch + nicotine lozenge) has not been previously evaluated.

There is a great deal of evidence that smoking cessation medications increase the success of a quit smoking attempt.[1–4] The 2008 Update to the Public Health Service (PHS) *Treating Tobacco Use and Dependence Clinical Practice Guideline* found that five nicotine replacement therapies (NRT's) and two non-nicotine replacement first-line pharmacotherapies (bupropion SR, varenicline) reliably increase abstinence rates relative to a placebo control.[1] However, less is known about the *relative* efficacies of these medications. This limitation is due, in part, to a lack of clinical trials that provide "head-to-head" comparisons of different pharmacotherapies within the same study. Cessation studies of individual medications differ in myriad respects which makes it difficult to gauge effectiveness across treatments, even when the individual studies contain a placebo control for the medication. Meta-analyses that attempt to account for interstudy differences may yield conclusions that conflict markedly with large-scale head-to-head trials.[5] Without evidence based on head-to-head comparisons, clinicians and smokers lack a strong empirical basis for recommending or selecting from amongst the available smoking cessation medications. Finally, the small number of studies offering head-to-head comparisons yield some conflicting evidence.[6, 7]

Five pharmacotherapies were selected for comparison in this placebo controlled trial: Nicotine Lozenge, Nicotine Patch, Bupropion, Nicotine Patch + Nicotine Lozenge, and Bupropion + Nicotine Lozenge.[1] These therapies were selected for several reasons. The nicotine lozenge

[1]This study was designed and conducted prior to varenicline's release as an FDA-approved medication. However, it should be noted that varenicline is a highly efficacious smoking cessation pharmacotherapy.[1, 4, 8, 9]

NIH-PA Author Manuscript NIH-PA Author Manuscript NIH-PA Author Manuscript

was selected because there was limited evidence regarding its efficacy. The 2008 PHS Guideline Update identified only one randomized placebo-controlled trial evaluating the lozenge, and thus gave the lozenge a “B” level strength-of-evidence rating. The single placebo-controlled trial on the lozenge suggests that it is both acceptable to smokers and highly efficacious.[8]

The nicotine patch was included in this study because it is the most commonly used pharmacotherapy for smoking cessation.[6, 9, 10] Given that so many smokers use the nicotine patch, it is important to determine the efficacy of other agents relative to the patch. Finally, it is important to examine the efficacy of the patch because recent data suggest that patch efficacy may have declined over the past 5–10 years.[11–13]

Bupropion SR was selected because there is modest evidence that it may be more efficacious than the nicotine patch, 11, [14] suggesting that we might observe differential efficacy in the current trial. Also, bupropion has never been directly compared with the nicotine lozenge. Finally, smokers could be encouraged to seek out this prescribed agent, and insurers and health care systems could be encouraged to make this treatment more widely available, if it could be demonstrated that bupropion is more efficacious than over-the-counter (OTC) medication (e.g., the nicotine patch or lozenge).

In addition to the three monotherapies, we tested two combination therapies. Research has generally supported the efficacy of NRT combinations. The 2008 PHS Guideline identified long-term (> 14 weeks) nicotine patch paired with either nicotine gum or nicotine nasal spray as efficacious relative to placebo and relative to the nicotine patch alone.1 A recent Cochrane meta-analysis also found that nicotine patch plus “fast acting” NRT was more effective than monotherapy.2 Combination NRT could be superior to monotherapy for several reasons. For instance, the uses of two NRT’s might produce more adequate nicotine replacement (i.e., higher blood nicotine levels 15) than a single NRT, although high-dose nicotine patches have not been shown to produce higher abstinence rates than standard-dose patches on a consistent basis.1, 2 Or, it is possible that each type of agent works through a different mechanism, so that having two types produces additive effects. The patch, for instance, produces a steady-state supply of nicotine to prevent severe nicotine withdrawal, and *ad libitum* NRT’s (gum, lozenge) provide a means for coping with situational challenges and transient urges to smoke.[15], 16

The combination of bupropion plus the nicotine lozenge was also examined because of promising initial results with the nicotine lozenge as a monotherapy.[17] Also, the combination of the nicotine patch + bupropion was found to be highly efficacious in the 2008 PHS Guideline meta-analysis (OR = 2.5).[1] It is possible that an NRT that permits *ad libitum* dosing might produce even better outcomes.

The current research evaluated the five pharmacotherapy interventions on a range of outcome indices, including 6 month 7-day point-prevalence quit rate, a traditional standard for assessing efficacy of smoking cessation interventions.1 This research also determined whether the medications were efficacious in helping a smoker achieve early success (i.e., being able to quit for a week following the quit date) or any success at all (i.e., being able to establish abstinence for at least 1 day during the first week of a quit attempt). In addition, outcomes assessed whether different medications increased the time to first lapse (the first cigarette smoked after quitting) or the time to relapse (smoking on 7 consecutive days following the quit day) or prevented a lapse from becoming a relapse. These different outcomes may help researchers understand the mechanisms of action of different medications and may be helpful in cessation counseling. For instance, if a medication reduces the transition of a lapse to a relapse (e.g., 18), smokers could be urged to continue medication use despite lapsing.

NIH-PA Author Manuscript
NIH-PA Author Manuscript
NIH-PA Author Manuscript

In sum, this research attempted to gauge the relative efficacies of widely available smoking cessation medications. The results were intended to permit more informed decisions about the selection and use of smoking cessation pharmacotherapies as a means of enhancing treatment effectiveness. .

Methods

Participants

Participants were 1504 smokers (58% female, 83% Caucasian) who agreed to participate in a 3-year smoking cessation (Year 1) and health outcomes study (Years 2 and 3) conducted in Madison and Milwaukee, WI (principal investigator: Timothy Baker, Ph.D.). Adult smokers were recruited via TV, radio and newspaper advertisements, flyers, earned media including press conferences, and TV and radio news interviews from January, 2005 to June, 2007. Inclusion criteria included smoking greater than nine cigarettes per day on average for at least the past 6 months, having an alveolar carbon monoxide (CO) level greater than 9, and being motivated to quit smoking. Exclusion criteria included using any form of tobacco other than cigarettes, currently taking bupropion, or having a current psychosis or schizophrenia diagnosis. In addition, participants were excluded if they had medical contraindications for any of the study medications, including high alcohol consumption (six drinks per day on 6 or 7 days of the week), a history of seizure, high blood pressure (> 160/100), bipolar disorder, an eating disorder, a recent cardiac event, or allergies to any of the medications. Only one person per household could participate. Finally, pregnant or breast-feeding women were not eligible for participation; eligible female participants had to agree to take steps to prevent pregnancy during the medication treatment phase of the study. All participants provided written informed consent and the study was approved by the University of Wisconsin Health Sciences Institutional Review Board.

Procedures

Interested smokers phoned a central research office, where they completed a telephone screen to determine eligibility. Participants who passed the telephone screen were invited to an informational session where they provided written informed consent. Next, participants completed three in-person baseline sessions. During the first baseline session, participants underwent further screening including collection of relevant medical history information, vital signs measurements, and a carbon monoxide (CO) breath test. Additionally, at this visit, participants completed several demographic, smoking history, and tobacco dependence questionnaires.

After additional medical assessments at two more baseline sessions (e.g., brachial artery reactivity, carotid intima media thickness, and small particle lipoprotein testing), participants were randomized to one of six treatment conditions: 1) Bupropion SR (150 mg, bid for 9 weeks total: 1week pre-quit and 8 weeks post-quit); 2) Nicotine Lozenge (2 or 4 mg, based on appropriate dose for dependence level per package instructions, for 12 weeks post-quit); 3) Nicotine Patch (24-hour patch; 21, 14, and 7mg; titrated down over 8 weeks post-quit); 4) Nicotine Patch (24-hour patch; 21, 14, and 7mg; titrated down over 8 weeks post-quit) + Nicotine Lozenge (2 or 4 mg, based on appropriate dose for dependence level per package instructions, for 12 weeks post-quit) combination therapy; 5) Bupropion SR (150 mg, bid for 9 weeks total: 1week pre-quit and 8 weeks post-quit) + Nicotine Lozenge (2 or 4 mg, based on appropriate dose for dependence level per package instructions, for 12 weeks post-quit) combination therapy; or 6) Placebo. It should be noted that “pre-quit” and “post-quit” in this manuscript refer, respectively, to the periods of time prior to and following a patient’s targeted quit date. There were five distinct placebo conditions, matched to each of the active treatment conditions (i.e., placebo bupropion, placebo lozenge, placebo patch, placebo patch + lozenge

and placebo bupropion + lozenge; see Figure 2). Participants received study medication at each study visit and returned any unused medication at the following visit. Randomization was double-blind and used a blocked randomization scheme with gender and self-reported race (white/non-white) as the blocking variables. Staff did not know to which type(s) of medication (i.e., patch, pill, and/or lozenge) a participant would be assigned until the moment of randomization, and study staff were blinded to whether the medication was active or placebo. In addition to pharmacotherapy, all participants received six one-on-one counseling sessions based upon the PHS Guideline.[1] Study staff who provided counseling and conducted study sessions were bachelor-level trained case managers, supervised by a licensed clinical psychologist. Sessions lasted 10–20 minutes and occurred over 7 weeks with the first two counseling sessions occurring prior to quitting and the subsequent five occurring on the quit date or thereafter (see Figure 1). The last baseline visit, where randomization occurred and medication was dispensed, took place between 8 and 15 days pre-quit to ensure the bupropion up-titration schedule could be completed. Participants were instructed to start medications on the designated quit date, except for bupropion SR, which they were instructed to initiate 1 week prior to the quit date as per the package insert instructions. Participants had study visits on their quit day, and at 1-, 2-, 4- and 8-weeks post-quit. At study visits, vital signs, adverse events and smoking status were all recorded.

Measures

Demographics and Smoking—Baseline questionnaires assessed demographics, smoking history, and nicotine dependence. The demographics questionnaire tapped characteristics such as gender, race (smokers were asked which race they most strongly identified with), Hispanic ethnicity (i.e., reporting at least one parent of Hispanic origin), income, education level and age. A smoking history questionnaire provided information about smoking behavior, smoking restrictions at home and work, self-efficacy to quit smoking, spouse smoking patterns, and motivation to quit smoking. Nicotine dependence questionnaires included the Fagerstrom Test of Nicotine Dependence (FTND) 19, the Nicotine Dependence Syndrome Scale (NDSS) 20, the Tobacco Dependence Screener (TDS) 21, and the Wisconsin Inventory of Smoking Dependence Motives (WISDM) 22.

Smoking Status—Smoking status was assessed both as 7-day point prevalence abstinence (“Have you smoked at all, even a puff, in the last seven days?”) and continuous abstinence (smoking at all since the target quit day [TQD]) using a smoking calendar and the time-line follow-back method.23, 24 All of participants’ self-reports of smoking status during study visits were confirmed by an expired carbon monoxide (CO) level of less than 10 parts per million measured using a Micro-3 Smokerlyzer (Bedfont Scientific, USA, Williamsburg, VA).

Analytic plan

All analyses were conducted using SPSS 15.0 software (SPSS, Inc.). After verifying that all treatment groups were similar across demographic and tobacco-related variables, we evaluated treatment effects on multiple outcome variables including: 1) CO-confirmed 7-day point-prevalence abstinence at 1 week post-quit, end of treatment (8 weeks post-quit) and 6 months post-quit; 2) number of days to lapse (latency to smoke a first cigarette after the TQD); 3) number of days to relapse (latency to smoke on 7 consecutive days after the TQD); 4) latency to relapse after the first lapse; and 5) initial cessation (whether the participant went at least 1 day without smoking in the first week post-quit; due to missing data n = 1424 for this outcome). Logistic regression was used for dichotomous outcomes (e.g., 7-day point-prevalence abstinence) while Cox regression was used for continuous outcomes (e.g., latency to lapse).

We conducted eleven comparisons for each outcome, which constituted a family of analyses that compared: each active treatment to placebo (five comparisons), each monotherapy with

NIH-PA Author Manuscript NIH-PA Author Manuscript NIH-PA Author Manuscript

NIH-PA Author Manuscript

NIH-PA Author Manuscript

NIH-PA Author Manuscript

each other (three comparisons), the two combination therapies with one another (two comparisons), and a composite of the monotherapies to the bupropion + lozenge combination and the patch + lozenge combination (two comparisons). To control for the family-wise error when conducting multiple tests, we used a Bonferroni-corrected *p*-value ($p = .0045$) for the eleven comparisons for an overall $\alpha = .05$ (all tests two-sided). We report both adjusted as well as unadjusted *p*-values. All analyses were conducted using the intent-to-treat principle such that all smokers who were randomized to a treatment were included in the analyses and individuals with missing data were considered to be smoking. Analyses were also conducted controlling for race (white vs. non-white), gender, and site. This study had a priori power = .88 to detect a clinically significant improvement in abstinence rates of 12% at 6-months post-treatment (e.g., 12 vs. 24%), with no correction for multiple comparisons ($\alpha = .05$) and power = .60 for the Bonferroni-corrected $\alpha = .005$. To detect an improvement in abstinence rates of 15% (e.g., 12 vs. 27%) this study had a priori power = .97 for $\alpha = .05$ and power = .84 for $\alpha = .005$.

Results

Table 1 provides demographics and smoking history data for the 1504 adult smokers who were randomized in this double-blind placebo controlled smoking cessation study. There were no statistically significant differences between the active and placebo treatment groups on age, cigarettes smoked per day, FTND score, baseline CO level, gender, marital status, race, Hispanic origin, or education. Figure 2 presents the CONSORT data.

There were no statistically significant differences amongst the placebo conditions in 7-day point-prevalence outcomes at 1 week, end of treatment (EOT) or 6-months post-quit. Therefore, for all subsequent analyses, the placebo conditions were combined into a unified placebo condition.

There was a significant main effect for study site, such that, relative to Madison, Milwaukee had significantly lower 7-day point-prevalence abstinence rates at all three follow-up time points. However, there were no treatment by site interactions and analyses that controlled for site produced results similar to those in the uncontrolled analyses.

Efficacy

Comparing all five active treatments to the placebo group in 7-day point-prevalence analyses, using an *uncorrected p*-value of .05, logistic regression analysis indicated that all active treatments produced higher rates of initial cessation and higher 7-day point-prevalence abstinence rates at 1 week, EOT and 6 months post-quit (with the exception of the lozenge at 1 week) relative to placebo (see Tables 2 and 3). The odds ratios at 6-months post-quit were 1.63 for bupropion, 1.76 for lozenge, 1.83 for patch, 1.74 for bupropion + lozenge and 2.34 for patch + lozenge. With the corrected *p*-value of .0045, only the patch and the two combination therapies were efficacious at 1 week and EOT, and only the patch + lozenge condition was efficacious at 6 months post-quit (see Tables 2 and 3). Using the corrected *p*-values, all treatments, except the lozenge, significantly increased the rates of initial cessation (not smoking for at least 1 day in the first week post-quit). The same effects were obtained when logistic regression analyses controlled for race, gender and site.

Survival analyses (Cox regression) revealed that relative to placebo, all active treatments significantly increased the number of days to relapse using both the unadjusted ($p < .05$) as well as the Bonferroni-corrected *p*-value (p's $\leq .001$). Only the two combination conditions significantly increased the number of days to lapse, relative to placebo, when using the Bonferroni-corrected *p*-value (p's $< .001$). Figure 3 illustrates the survival curves for latency to relapse. The survival curves for latency to lapse and for latency to relapse following a lapse

NIH-PA Author Manuscript NIH-PA Author Manuscript NIH-PA Author Manuscript

were similar to the survival curves for latency to relapse. All active treatments increased the latency to relapse following the first lapse (*p*'s ≤ .003) with the exception of the lozenge (*Wald* = 6.39, *p* = .011, *OR* = .73, 95% *CI* = .75, .93). The same effects were obtained when analyses controlled for race, gender and site.

The bupropion + lozenge condition and the patch + lozenge condition were both compared to a composite monotherapy condition to determine whether either of the combination conditions was superior to monotherapy. Results of logistic regression analyses revealed that, relative to the monotherapies, patch + lozenge produced significantly higher initial cessation rates and EOT abstinence rates (see Table 3), using the Bonferroni-corrected *p*-value. There were no other differences between the combination conditions and the composite monotherapy condition. The results were similar after controlling for race, gender and site. It should be noted that there were no significant differences either between the two combination conditions or among the monotherapy conditions at any of the timepoints using the Bonferroni-corrected *p*-value.

With respect to the latency outcome variables, Cox regression analyses revealed that patch + lozenge users had a greater latency to lapse, relative to the composite monotherapy condition (*Wald* = 7.31, *p* = .007, *OR* = .80, *95% CI* = .78, .94); a similar effect was found for number of days to relapse (*Wald* = 5.45, *p* = .02, *OR* = .79, *95% CI* = .64, .96). These differences were not significant with the Bonferroni correction ($p < .005$). Even without the Bonferroni correction, there were no differences amongst the combination and monotherapy groups in latency to relapse after smoking the first cigarette (i.e., after lapsing). The same effects were obtained when analyses controlled for race, gender and site.

Medication Use

At each visit, participants were given additional medication and asked to return any unused medication. We computed the percentage of medication each participant used by subtracting the amount of medication the participant returned from the amount of medication given to the participant and then dividing that by the total amount of medication given to the participant. On average, participants used approximately 77% of the medication given out over the course of the study (placebo = 75%, patch = 86%, bupropion = 85%, lozenge = 67%, bupropion + lozenge = 77% and patch + lozenge = 74%). A one-way ANOVA revealed significant differences in the amount of medication used by treatment condition, $F(5, 1187) = 17.64$, $p < .001$. Post-hoc Tukey tests revealed that individuals in the lozenge condition used significantly less medication (67% of the medication given) than individuals in any of the other treatment conditions (p's ranged from .03 to less than .001).

Safety

The most common adverse events varied by treatment group but were consistent with previous research. In the placebo condition the most common adverse events were headaches, skin irritation in the patch condition, sleep disturbances/abnormal dreams in the bupropion condition, nausea in the lozenge condition, sleep disturbances/abnormal dreams in the bupropion + lozenge condition and both sleep disturbances/abnormal dreams and skin irritation in the patch + lozenge condition (see Table 4 for frequencies). Participants in the combination conditions (patch + lozenge and bupropion + lozenge) reported more adverse events than those in either the monotherapy or placebo groups. There were multiple serious adverse events (SAEs) during the 6 month period following the TQD, but very few were attributed to study medication. (See Table 5 for a description of the specific SAEs.) Four people (0.27%) withdrew from the study due to events related to medication: one in the bupropion condition because it interacted with other antidepressants and the participant's physician requested that the participant withdraw, one in the bupropion condition because of heartburn, one from a

"negative experience" on the placebo medication and one in the bupropion + lozenge condition due to hospitalization for seizures.

Discussion

A principal goal of this research was to identify particularly efficacious smoking cessation pharmacotherapy interventions amongst the five different treatments tested in a head-to-head comparison. The nicotine patch + lozenge combination emerged as the treatment with the strongest support. Its odds ratio at 6-months postquit was 2.34, while the next highest odds ratio was 1.83 (for the nicotine patch). The nicotine patch + lozenge combination emerged as the only efficacious treatment (after Bonferroni correction for multiple tests), relative to placebo, at 6-months post-quit. In addition, relative to a monotherapy composite, the patch + lozenge condition produced higher initial cessation rates and end-of-treatment 7-day point-prevalence rates using the Bonferroni-corrected alpha level. The patch + lozenge combination also tended to produce more positive outcomes than any other condition, active or placebo, on measures such as days to lapse and days to relapse (see Figure 3); these differences did not exceed protection levels for multiple comparisons, however. These effects are consistent with previous research showing that the patch + ad lib NRT increases the time to relapse.[25] Finally, while there was substantial evidence that the patch + lozenge combination was highly efficacious relative to the placebo condition, it is important to note that it did not differ significantly in 6-month outcome from the other active cessation treatments in head-to-head comparisons.

While the patch + lozenge combination was notably efficacious relative to placebo, the other pharmacotherapies were also significantly effective if tested with unadjusted *p*-values (see Table 3). These pharmacotherapies, with odds ratios ranging from 1.63 to 1.83, would have been found to be efficacious relative to placebo had they been tested in a typical randomized clinical trial involving only a single active treatment and a placebo control. Thus, the current results suggest that there was a relatively strong effect of the patch + lozenge versus placebo, rather than unusually weak effects of the other interventions. This pattern of findings should be evaluated in light of the relatively high abstinence rates that occurred in the placebo condition. At 6-months post-quit, participants in the placebo group achieved a 22.2% abstinence rate. This abstinence rate is larger than many 6-month abstinence rates in active treatment conditions in other studies (e.g., 7, 11). The success of placebo may have been due to the intensive counseling participants received (six sessions, totaling more than 60 minutes of counseling) 26, or due to the high level of motivation required to participate in a 3-year longitudinal trial.

During treatment, the patch, bupropion + lozenge, and patch + lozenge conditions were all significantly more efficacious than placebo, with the familywise error correction. However, after treatment was discontinued, by 6 months post-quit, only the patch + lozenge remained efficacious. These findings agree with the 2008 Guideline Update meta-analyses that showed that the combination of long-term patch + gum or spray had the highest odds ratio for efficacy (6-month abstinence) of any of the evaluated pharmacotherapies (monotherapies and combination therapies) when tested against a placebo control condition (OR = 3.6).1 These findings suggest that long-term pharmacotherapy (> 14 weeks), particularly with the nicotine patch, may be important given that the effects of bupropion + lozenge diminished significantly once participants stopped using them at the end of treatment. Future research should examine relapse dynamics following the discontinuation of treatment; it would be important to know if treatment discontinuation was more consequential for some pharmacotherapies than for others (e.g., 27, 28). Future research should also address the promising issue of using pharmacotherapy prior to the quit attempt.1, 2

NIH-PA Author Manuscript NIH-PA Author Manuscript NIH-PA Author Manuscript

NIH-PA Author Manuscript
NIH-PA Author Manuscript
NIH-PA Author Manuscript

One of the outcomes assessed in this research was whether pharmacotherapy treatment could help people achieve at least one day of abstinence (initial cessation). The ability to achieve initial abstinence is not only a stepping stone to successful quitting, but research suggests that duration of abstinence in prior quit attempts enhances success in subsequent attempts.[29–31] In this regard, the patch and patch + lozenge conditions resulted in the highest rates of initial abstinence (using adjusted *p*-values; see Table 2). This finding is consistent with earlier findings that the high-dose nicotine patch was significantly more effective in helping smokers achieve initial abstinence, relative to placebo.[18] It should be noted that bupropion alone and bupropion + lozenge also had significantly higher initial cessation rates relative to placebo using adjusted *p*-values.

Previous research on combination NRT paired the patch with either nicotine gum [25, 32], nicotine nasal spray [7, 33] or nicotine inhaler [34, 35]. The present results suggest that the nicotine lozenge can also be effective as an adjuvant to the nicotine patch. The key seems to be that an *ad libitum*, or "as needed", agent must be paired with the patch; simply using higher patch doses does not seem to augment outcomes to the same degree.[36–39] While the nicotine lozenge appears to be an effective patch adjuvant, its performance as a monotherapy was not as impressive as the patch. For instance, the lozenge did not produce significantly higher cessation rates than placebo in either the first week of treatment or at EOT (with alpha adjustment; see Table 3).

While overall medication adherence reached an average of 77%, there were significant differences in rates of use of the different medications. Bupropion and the nicotine patch had the highest use rates, followed by the two combinations and the nicotine lozenge had the lowest use rates. These findings suggest that smokers are especially unlikely to use prn medications adherently (i.e., a recommendation of 9 lozenges per day). This is consistent with other literature that suggests an inverse relation between number of doses prescribed and medication adherence[40] and consistent with research showing a direct positive relation between medication adherence and cessation outcome.41–46

The pharmacotherapy interventions used in this research appear to be safe and well tolerated. Only 4 individuals out of 1504 withdrew from the study for medication-related reasons (see above). There were more adverse events, however, among individuals in the combination pharmacotherapy versus the monotherapy or placebo conditions (see Table 4). Combination therapy did not appear to increase serious adverse events or study withdrawal in comparison to monotherapy. These findings agree with prior research that supported the safety and patient acceptance of combination NRT (e.g., [15], 25, 32, 47, 48).

One limitation of this study is that treatment took place in the context of a longitudinal study, which may have selected for participants with greater motivation to quit than smokers in the general population. In addition, treatment lasted only 8 weeks (with the exception of the nicotine lozenge, which lasted for 12 weeks). Future research will be needed to determine whether long-term usage of these pharmacotherapies improves efficacy (although some evidence suggests that longer use is not efficacious1). A final limitation is that the study did not include varenicline amongst the tested medications (varenicline was not FDA-approved at the time of study initiation), and therefore it is unknown how these agents would have fared relative to varenicline, the monotherapy designated as most effective by the 2008 PHS Guideline.1 The results do suggest the importance of testing varenicline against a combination of the nicotine patch and an *ad libitum* NRT medication because the current study identified this intervention as especially efficacious relative to placebo.

NIH-PA Author Manuscript NIH-PA Author Manuscript NIH-PA Author Manuscript

Conclusions

In this study assessing five different pharmacotherapy interventions, the nicotine patch + lozenge produced the greatest benefit relative to placebo. These findings, plus recent meta-analyses published in the 2008 PHS Guideline Update, suggest that a combination pharmacotherapy comprising the nicotine patch and an *ad libitum* NRT should be routinely considered for use as a smoking cessation treatment. In addition, this study illustrates that, while the patch has been used for smoking cessation for more than 20 years, it remains a highly efficacious pharmacotherapy for helping smokers quit.

Acknowledgments

Stevens S. Smith has received research support from Elan Corporation, plc. Timothy B. Baker has served as an investigator on research projects sponsored by pharmaceutical companies including Sanofi-Synthelabo, Pfizer, Inc. and Nabi Biopharmaceuticals. Douglas E. Jorenby has received research support from the National Institute on Drug Abuse, the National Cancer Institute, Pfizer, Inc., Sanofi-Synthelabo, and Nabi Biopharmaceuticals. He has received support for educational activities from the National Institute on Drug Abuse and the Veterans Administration, and consulting fees from Nabi Biopharmaceuticals. Michael C. Fiore has received honoria or consulting fees from pharmaceutical companies. He has served as an investigator on research studies at the University of Wisconsin that were funded by Pfizer, Sanofi-Synthelabo and Nabi Biopharmaceuticals. In 1998, the University of Wisconsin (UW) appointed Dr. Fiore to a named Chair funded by an unrestricted gift to UW from Glaxo Wellcome. All authors had full access to all of the data in the study and take responsibility for the integrity of the data and the accuracy of the data analysis.

This research was conducted at the University of Wisconsin, Madison and was supported by grant #P50 DA019706 from NIH/NIDA and by grant #M01 RR03186 from the General Clinical Research Centers Program of the National Center for Research Resources, NIH. Dr. Piper was supported by an Institutional Clinical and Translational Science Award (UW-Madison; KL2 Grant # 1KL2RR025012-01). Medication was provided to patients at no cost under a research agreement with GlaxoSmithKline (GSK); no part of this manuscript was written or edited by anyone employed by GSK. The authors are solely responsible for the analyses, content, and writing of this article.

References

1. Fiore, MC.; Jaen, CR.; Baker, TB., et al. Treating tobacco use and dependence: 2008 update. Rockville, MD: U.S. Department of Health and Human Services, U.S. Public Health Service; 2008.
2. Stead LF, Perera R, Bullen C, Mant D, Lancaster T. Nicotine replacement therapy for smoking cessation. Cochrane Database Syst Rev 2008;(1):CD000146. [PubMed: 18253970]
3. Hughes, Stead L, Lancaster T. Antidepressants for smoking cessation. Cochrane Database Syst Rev 2007;(1):CD000031. [PubMed: 17253443]
4. Cahill K, Stead L, Lancaster T. Nicotine receptor partial agonists for smoking cessation. Cochrane Database Syst Rev 2007;(1):CD006103. [PubMed: 17253581]
5. LeLorier J, Gregoire G, Benhaddad A, Lapierre J, Derderian F. Discrepancies between meta-analyses and subsequent large randomized, controlled trials. N Engl J Med 1997 Aug 21;337(8):536–542. [PubMed: 9262498]
6. West R, Hajek P, Nilsson F, Foulds J, May S, Meadows A. Individual differences in preferences for and responses to four nicotine replacement products. Psychopharmacology (Berl) 2001 Jan 1;153(2): 225–230. [PubMed: 11205423]
7. Croghan GA, Sloan JA, Croghan IT, et al. Comparison of nicotine patch alone versus nicotine nasal spray alone versus a combination for treating smokers: a minimal intervention, randomized multicenter trial in a nonspecialized setting. Nicotine and Tobacco Research 2003 Apr;5(2):181–187. [PubMed: 12745490]
8. Shiffman S, Dresler CM, Hajek P, Gilburt SJA, Targett DA, Strahs KR. Efficacy of a nicotine lozenge for smoking cessation. Arch Intern Med 2002;162(11):1267–1276. [PubMed: 12038945]
9. Centers for Disease Control and Prevention. Use of FDA-approved pharmacological treatments for tobacco dependence - United States, 1984–1998. MMWR 2000;49:665–668. [PubMed: 10943254]
10. Pierce JP, Gilpin E, Farkas AJ. Nicotine patch use in the general population: results from the 1993 California Tobacco Survey. Journal of the Nationall Cancer Institute 1995 Jan 18;87(2):87–93.

NIH-PA Author Manuscript

NIH-PA Author Manuscript

NIH-PA Author Manuscript

11. Jorenby DE, Leischow SJ, Nides MA, et al. A controlled trial of sustained-release bupropion, a nicotine patch, or both for smoking cessation. New England Journal of Medicine 1999 Mar 4;340 (9):685–691. [PubMed: 10053177]

12. Pierce JP, Gilpin EA. Impact of over-the-counter sales on effectiveness of pharmaceutical aids for smoking cessation. JAMA 2002 Sep 11;288(10):1260–1264. [PubMed: 12215133]

13. Irvin JE, Hendricks PS, Brandon TH. The increasing recalcitrance of smokers in clinical trials II: Pharmacotherapy trials. Nicotine Tob Res 2003 Feb;5(1):27–35. [PubMed: 12745504]

14. Gold PB, Rubey RN, Harvey RT. Naturalistic, self-assignment comparative trial of bupropion SR, a nicotine patch, or both for smoking cessation treatment in primary care. Am J Addict. Fall 2002;11 (4):315–331.

15. Sweeney CT, Fant RV, Fagerstrom KO, McGovern JF, Henningfield JE. Combination nicotine replacement therapy for smoking cessation: Rationale, efficacy and tolerability. CNS Drugs 2001;15 (6):453–467. [PubMed: 11524024]

16. Shiffman S, Shadel WG, Niaura R, et al. Efficacy of acute administration of nicotine gum in relief of cue-provoked cigarette craving. Psychopharmacology (Berl) 2003 Apr;166(4):343–350. [PubMed: 12601502]

17. Shiffman S, Dresler CM, Rohay JM. Successful treatment with a nicotine lozenge of smokers with prior failure in a pharmacological therapy. Addiction 2004;98:83–92. [PubMed: 14678066]

18. Shiffman S, Scharf DM, Shadel WG, et al. Analyzing milestones in smoking cessation: illustration in a nicotine patch trial in adult smokers. J Consult Clin Psychol 2006 Apr;74(2):276–285. [PubMed: 16649872]

19. Heatherton TF, Kozlowski LT, Frecker RC, Fagerstrom KO. The Fagerstrom Test for Nicotine Dependence: a revision of the Fagerstrom Tolerance Questionnaire. Br J Addict 1991 Sep;86(9): 1119–1127. [PubMed: 1932883]

20. Shiffman S, Waters A, Hickcox M. The Nicotine Dependence Syndrome scale: A multidimensional measure of nicotine dependence. Nicotine & Tobacco Research 2004 Apr;6(2):327–348. [PubMed: 15203807]

21. Kawakami N, Takatsuka N, Inaba S, Shimizu H. Development of a screening questionnaire for tobacco/nicotine dependence according to ICD-10, DSM-III-R, and DSM-IV. Addict Behav 1999 Mar–Apr;24(2):155–166. [PubMed: 10336098]

22. Piper, Piasecki TM, Federman EB, et al. A multiple motives approach to tobacco dependence: The Wisconsin Inventory of Smoking Dependence Motives (WISDM-68). Journal of Consulting and Clinical Psychology 2004;72(2):139–154. [PubMed: 15065950]

23. Sobell LC, Sobell MB, Leo GI, Cancilla A. Reliability of a timeline method: Assessing normal drinkers' reports of recent drinking and a comparative evaluation across several populations. Br J Addict 1988 Apr;83(4):393–402. [PubMed: 3395719]

24. Brigham J, Lessov-Schlaggar CN, Javitz HS, McElroy M, Krasnow R, Swan GE. Reliability of adult retrospective recall of lifetime tobacco use. Nicotine Tob Res 2008 Feb;10(2):287–299. [PubMed: 18236293]

25. Kornitzer M, Boutsen M, Dramaix M, Thijs J, Gustavsson G. Combined use of nicotine patch and gum in smoking cessation: a placebo-controlled clinical trial. Prev Med 1995 Jan;24(1):41–47. [PubMed: 7740014]

26. Lancaster T, Stead L, Silagy C, Sowden A. Effectiveness of interventions to help people stop smoking: Findings from the Cochrane Library. British Medcial Journal 2000 Aug 5;321(7257):355–358.

27. Tonstad S, Tonnesen P, Hajek P, Williams KE, Billing CB, Reeves KR. Effect of maintenance therapy with varenicline on smoking cessation: a randomized controlled trial. JAMA 2006 Jul 5;296(1):64–71. [PubMed: 16820548]

28. Hurt RD, Krook JE, Croghan IT, et al. Nicotine patch therapy based on smoking rate followed by bupropion for prevention of relapse to smoking. J Clin Oncol 2003 Mar 1;21(5):914–920. [PubMed: 12610193]

29. Alessi SM, Badger GJ, Higgins ST. An experimental examination of the initial weeks of abstinence in cigarette smokers. Exp Clin Psychopharmacol 2004 Nov;12(4):276–287. [PubMed: 15571445]

NIH-PA Author Manuscript

30. Nides MA, Rakos RF, Gonzales D, Murray RP, Tashkin DP, Bjornson-Benson WM. Predictors of initial smoking cessation and relapse through the first 2 years of the Lung Health Study. Journal of Consulting & Clinical Psychology 1995 Feb;63(1):60–69. [PubMed: 7896992]
31. Swan GE, Jack LM, Javitz HS, McAfee T, McClure JB. Predictors of 12-month outcome in smokers who received bupropion sustained-release for smoking cessation. CNS Drugs 2008;22(3):239–256. [PubMed: 18278978]
32. Puska P, Korhonen HJ, Vartiainen E, Urjanheimo EL, Gustavsson G, Westin A. Combined use of nicotine patch and gum compared with gum alone in smoking cessation - a clinical trial in North Karelia. Tobacco Control 1995;4:231–235.
33. Blondal T, Gudmundsson LJ, Olafsdottir I, Gustavsson G, Westin A. Nicotine nasal spray with nicotine patch for smoking cessation: randomised trial with six year follow up. Bmj 1999 Jan 30;318 (7179):285–288. [PubMed: 9924052]
34. Bohadana A, Nilsson F, Rasmussen T, Martinet Y. Nicotine inhaler and nicotine patch as a combination therapy for smoking cessation: a randomized, double-blind, placebo-controlled trial. Arch Intern Med 2000 Nov 13;160(20):3128–3134. [PubMed: 11074742]
35. Tonnesen P, Mikkelsen KL. Smoking cessation with four nicotine replacement regimes in a lung clinic. Eur Respir J 2000 Oct;16(4):717–722. [PubMed: 11106218]
36. Dale LC, Hurt RD, Offord KP, Lawson GM, Croghan IT, Schroeder DR. High-dose nicotine patch therapy. Percentage of replacement and smoking cessation. JAMA 1995 Nov 1;274(17):1353–1358. [PubMed: 7563559]
37. Kalman D, Kahler CW, Tirch D, Kaschub C, Penk W, Monti PM. Twelve-week outcomes from an investigation of high-dose nicotine patch therapy for heavy smokers with a past history of alcohol dependence. Psychol Addict Behav 2004 Mar;18(1):78–82. [PubMed: 15008689]
38. Killen JD, Fortmann SP, Davis L, Strausberg L, Varady A. Do heavy smokers benefit from higher dose nicotine patch therapy? Experimental and Clinical Psychopharmacology 1999 Aug;7(3):226–233. [PubMed: 10472510]
39. Jorenby DE, Smith SS, Fiore MC, et al. Varying nicotine patch dose and type of smoking cessation counseling. JAMA 1995 Nov 1;274(17):1347–1352. [PubMed: 7563558]
40. Claxton AJ, Cramer J, Pierce C. A systematic review of the associations between dose regimens and medication compliance. Clin Ther 2001 Aug;23(8):1296–1310. [PubMed: 11558866]
41. Lam TH, Abdullah AS, Chan SS, Hedley AJ. Adherence to nicotine replacement therapy versus quitting smoking among Chinese smokers: a preliminary investigation. Psychopharmacology (Berl) 2005 Feb;177(4):400–408. [PubMed: 15289997]
42. Mooney ME, Reus VI, Gorecki J, et al. Therapeutic drug monitoring of nortriptyline in smoking cessation: A multistudy analysis. Clin Pharmacol Ther. 2007 Aug 8;
43. Alterman AI, Gariti P, Cook TG, Cnaan A. Nicodermal patch adherence and its correlates. Drug Alcohol Depend 1999 Jan 7;53(2):159–165. [PubMed: 10080041]
44. Stapleton JA, Russell MA, Feyerabend C, et al. Dose effects and predictors of outcome in a randomized trial of transdermal nicotine patches in general practice. Addiction 1995 Jan;90(1):31–42. [PubMed: 7888977]
45. Killen JD, Fortmann SP, Davis L, Varady A. Nicotine patch and self-help video for cigarette smoking cessation. J Consult Clin Psychol 1997 Aug;65(4):663–672. [PubMed: 9256568]
46. Kenford SL, Fiore MC, Jorenby DE, Smith SS, Wetter D, Baker TB. Predicting smoking cessation. Who will quit with and without the nicotine patch. Journal of the American Medical Association 1994 Feb 23;271(8):589–594. [PubMed: 8301790]
47. Haustein K, Krause J, Haustein H, Rasmussen GI, Cort N. Comparison of the effects of combined nicotine replacement therapy vs. cigarette smoking in males. Nicotine & Tobacco Research 2003;5:195–203. [PubMed: 12745492]
48. Fagerstrom KO, Hughes JR. Nicotine concentrations with concurrent use of cigarettes and nicotine replacement: a review. Nicotine Tob Res 2002;(4 Suppl 2):S73–S79. [PubMed: 12573169]

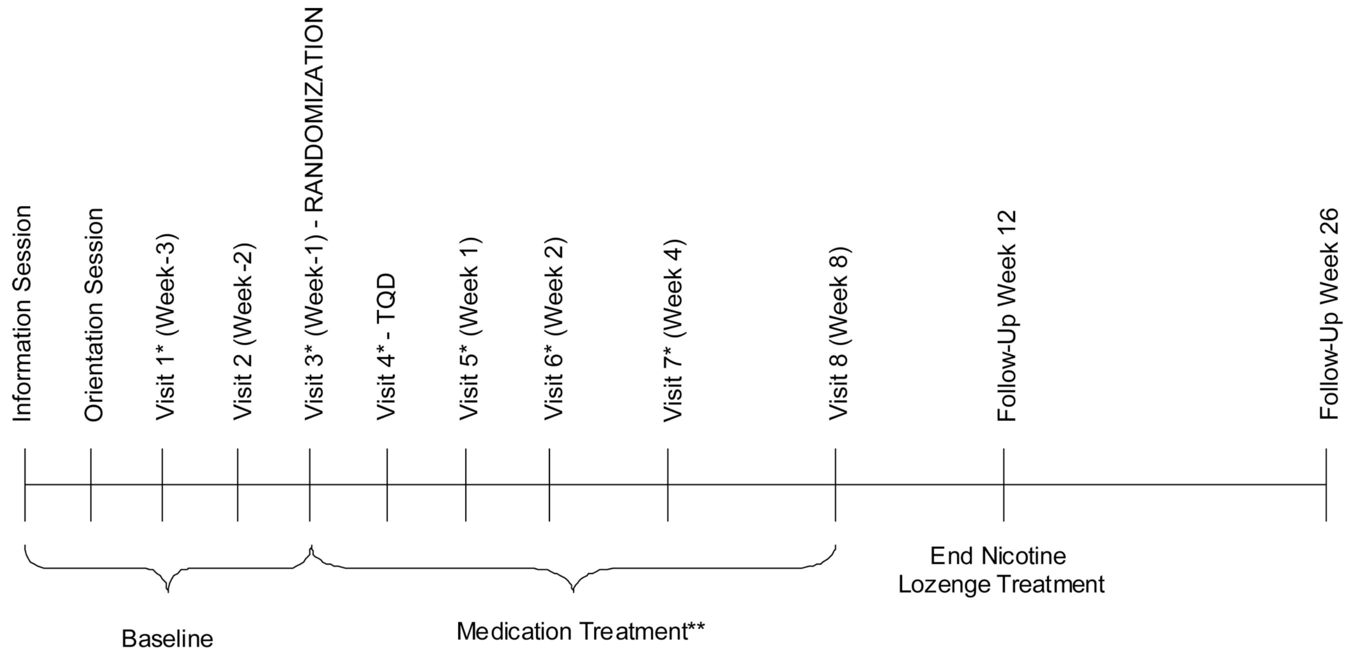

*Counseling session
**All treatments were administered through Week 8 except for the lozenge, which was continued until Week 12.

Figure 1. Study Time Line

Figure 1 illustrates the study timeline including all study visits for both assessment as well as treatment.

NIH-PA Author Manuscript
NIH-PA Author Manuscript
NIH-PA Author Manuscript

NIH-PA Author Manuscript
NIH-PA Author Manuscript
NIH-PA Author Manuscript

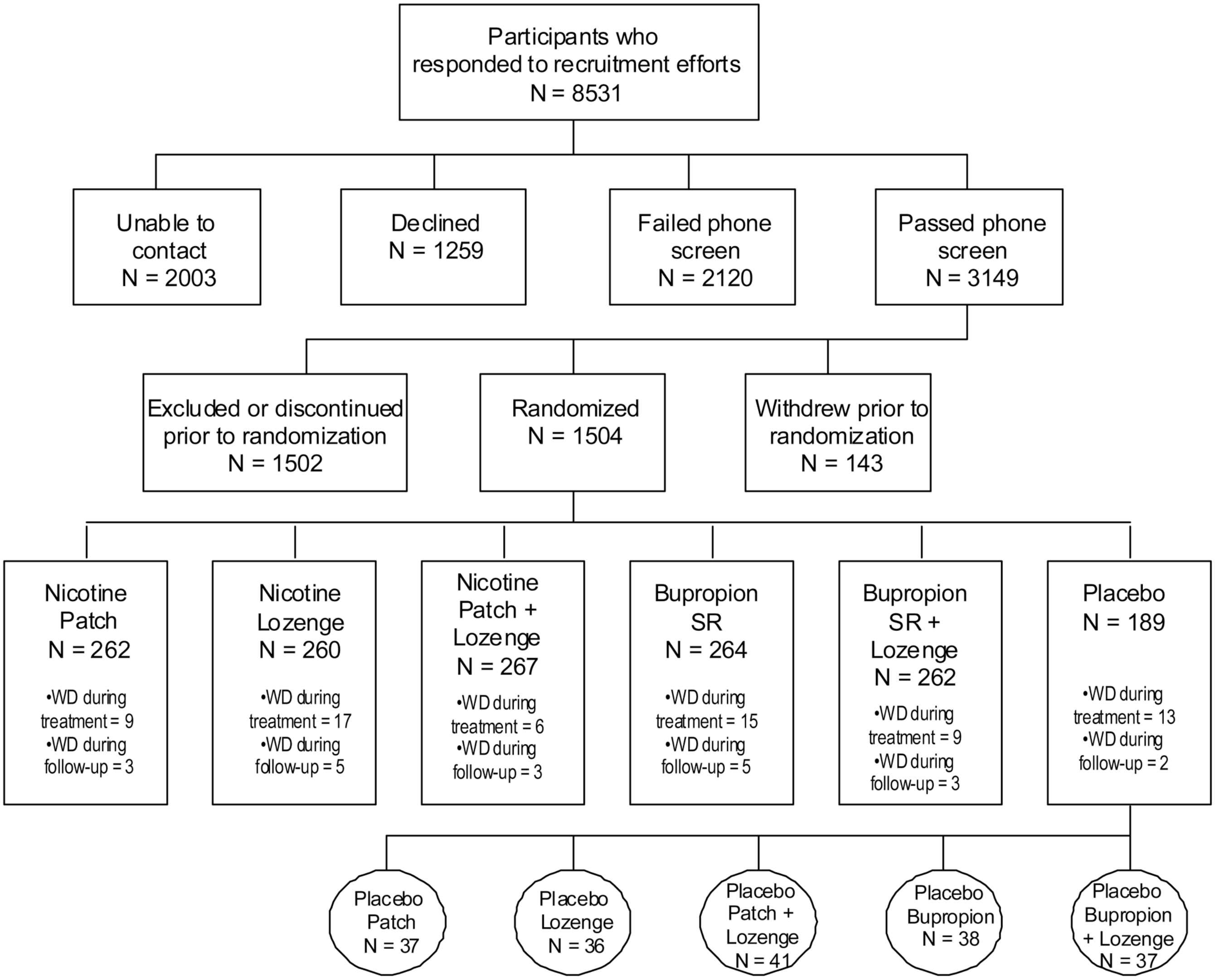

Figure 2. CONSORT and randomization information

Figure 2 is the CONSORT figure that documents the flow of participants from study recruitment, through screening, randomization and follow-up.

NIH-PA Author Manuscript
NIH-PA Author Manuscript
NIH-PA Author Manuscript

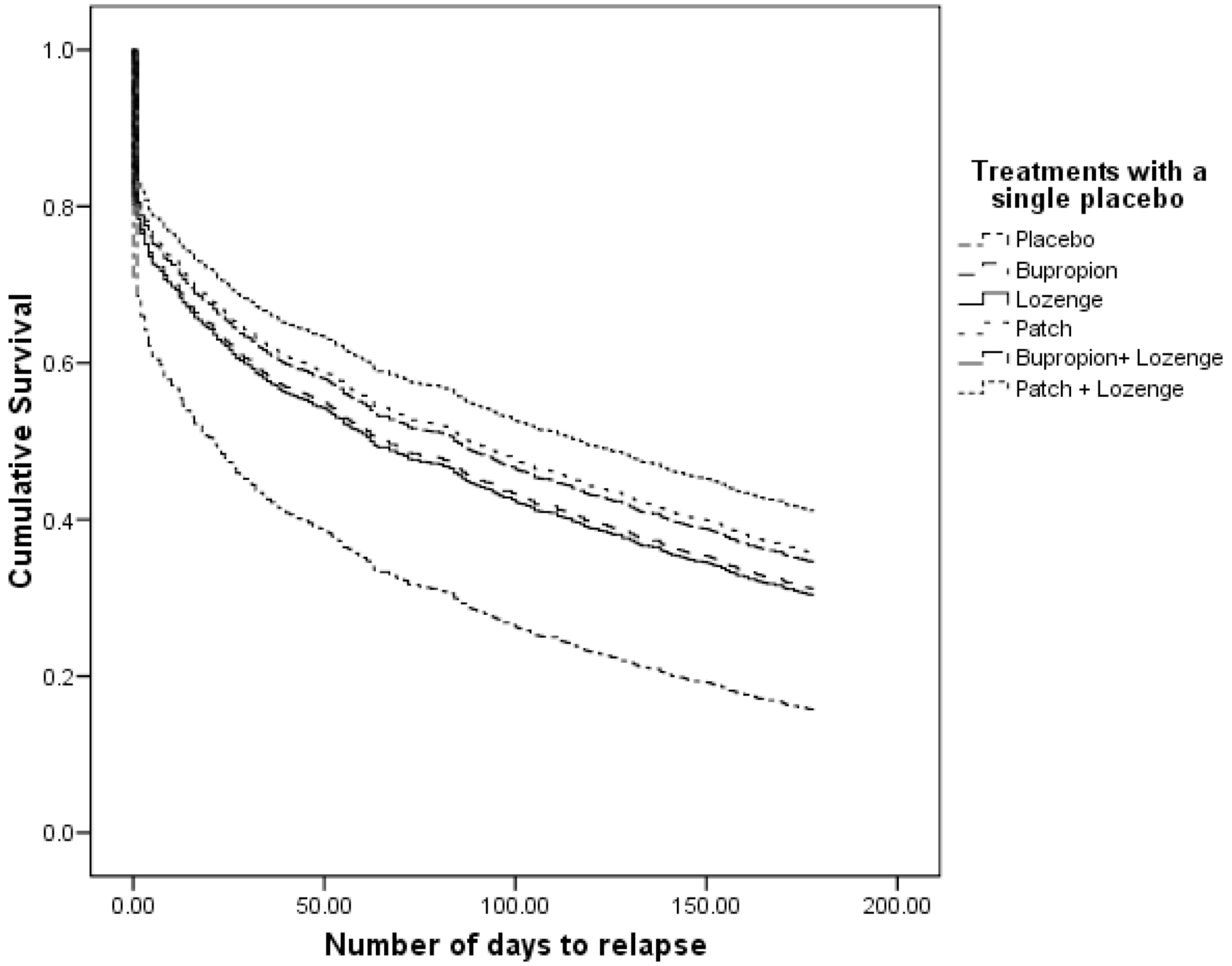

Figure 3. Latency to relapse (smoke on 3 consecutive days)

Figure 3 presents the survival curves for latency to relapse, or the number of days until the participants smoke on 7 consecutive days following the target quit day, for the 6 treatment conditions.

NIH-PA Author Manuscript

NIH-PA Author Manuscript

NIH-PA Author Manuscript

Table 1

Demographic and smoking history for the total sample and by treatment group.

	Total (N = 1504) N (%)	Placebo (N = 189) N (%)	Bupropion (N = 264) N (%)	Lozenge (N = 260) N (%)	Patch (N = 262) N (%)	Bupropion + Lozenge (N = 262) N (%)	Patch + Lozenge (N = 267) N (%)
Women	876 (58.2)	111 (58.7)	154 (58.3)	151 (58.1)	153 (58.4)	154 (58.8)	153 (57.3)
Married	667 (44.5)	77 (40.7)	117 (44.5)	116 (44.6)	114 (43.8)	128 (49.2)	115 (43.1)
Employed for wages	1,020 (67.8)	124 (65.6)	182 (68.9)	181 (69.6)	177 (67.6)	184 (70.2)	172 (64.4)
High school education only	353 (23.6)	48 (25.8)	52 (19.7)	61 (23.5)	73 (28.2)	61 (23.5)	58 (21.8)
Race/Ethnicity							
Hispanic[1]	42 (2.8)	7 (3.7)	7 (2.7)	5 (1.9)	6 (2.3)	8 (3.1)	9 (3.4)
White[2]	1,258 (83.9)	160 (84.7)	221 (83.7)	217 (83.5)	220 (84.6)	217 (83.5)	223 (83.5)
African-American[2]	204 (13.6)	20 (10.6)	35 (13.3)	38 (14.6)	35 (13.5)	38 (14.6)	38 (14.2)
Other race[2]	38 (2.6)	9 (4.7)	8 (3.0)	5(1.9)	5 (1.9)	5 (1.9)	6 (1.9)
	**Mean (SD)**	**Mean (SD)**	**Mean (SD)**	**Mean (SD)**	**Mean (SD)**	**Mean (SD)**	**Mean (SD)**
Age	44.7 (11.1)	43.1 (11.4)	43.9 (11.7)	45.3 (10.4)	44.9 (11.6)	45.3 (10.4)	44.2 (11.1)
Previous quit attempts	5.7 (9.7)	6.2 (13.1)	6.2 (10.4)	5.7 (11.2)	5.9 (10.1)	5.0 (5.2)	5.3 (6.5)
FTND Total	5.4 (2.1)	5.5 (2.2)	5.4 (2.2)	5.2 (2.2)	5.4 (2.1)	5.3 (2.1)	5.5 (2.1)
Cigarettes smoked/day	21.4 (8.9)	21.0 (8.3)	21.4 (8.2)	21.6 (9.1)	21.4 (9.2)	21.0 (8.5)	21.93 (9.6)
Baseline CO level	25.8 (12.5)	24.5 (13.3)	25.0 (10.7)	24.6 (12.0)	26.4 (12.3)	26.8 (13.6)	26.8 (12.2)

[1] Smokers who reported at least one parent was of Hispanic origin.

[2] Smokers were asked which race they most strongly identified with.

NIH-PA Author Manuscript
NIH-PA Author Manuscript
NIH-PA Author Manuscript

Table 2

CO-confirmed point-prevalent abstinence and initial cessation rates (%)

	Placebo	Bupropion	Lozenge	Patch	Bupropion + Lozenge	Patch + Lozenge
Initial cessation*	69.4	82.2	81.3	87.7	84.5	91.5
1 week	23.3	34.5	29.2	40.5	37.4	43.4
8 weeks (end of treatment)	30.2	40.2	40.4	44.7	50.4	53.6
6-months	22.2	31.8	33.5	34.4	33.2	40.1

*Initial cessation is defined as at least 1 day of abstinence during the first 7 days after the target quit day. Due to missing data n = 1424 for this analysis.

NIH-PA Author Manuscript NIH-PA Author Manuscript NIH-PA Author Manuscript

Table 3

Logistic regressions predicting initial cessation and point-prevalent abstinence.

	Initial Cessation				1 week post-quit				End of treatment (8 weeks post-quit)				6 months post-quit			
	Wald	P	OR	95% CI	Wald	P	OR	95% CI	Wald	P	OR	95% CI	Wald	P	OR	95% CI
**Relative to placebo**																
Bupropion	9.25	.002*	2.04	1.29, 3.22	6.52	.011	1.73	1.14, 2.64	4.75	.029	1.55	1.05, 2.31	5.01	.025	1.63	1.06, 2.51
Lozenge	7.60	.006	1.91	1.21, 3.03	1.97	.16	1.36	.89, 2.09	4.93	.026	1.57	1.05, 2.33	6.68	.010	1.76	1.15, 2.70
Patch	20.32	<.001*	3.14	1.91, 5.17	14.29	<.001*	2.24	1.47, 3.40	9.64	.002*	1.87	1.26, 2.77	7.70	.006	1.83	1.20, 2.81
Bupropion + Lozenge	13.14	<.001*	2.40	1.50, 3.84	10.00	.002*	1.97	1.29, 3.00	18.10	<.001*	2.35	1.59, 3.49	6.42	.011	1.74	1.13, 2.67
Patch + Lozenge	31.18	<.001*	4.73	2.74, 8.16	19.23	<.001*	2.53	1.67, 3.83	24.02	<.001*	2.67	1.80, 3.96	15.65	<.001*	2.34	1.54, 3.57
**Relative to monotherapies**																
Bupropion + Lozenge	.07	.793	1.05	.71, 1.56	.61	.43	1.12	.84, 1.50	5.95	.015	1.42	1.07, 1.88	.00	1.00	1.00	.74, 1.35
Patch + Lozenge	9.01	.003*	2.08	1.29, 3.36	6.46	.011	1.44	1.09, 1.92	11.19	.001*	1.61	1.22, 2.13	4.12	.042	1.35	1.01, 1.79
**Monotherapies relative to each other (first condition listed is the reference condition)**																
Patch vs. Lozenge	3.86	.049	.61	.37, .999	7.20	.007	.61	.42, .88	.97	.32	.84	.59, 1.19	.05	.83	.96	.67, 1.38
Bupropion vs. Lozenge	.07	.78	.94	.59, 1.48	1.65	.20	.79	.54, 1.14	.003	.96	1.01	.71, 1.43	.38	.54	.89	.62, 1.28
Patch vs. Bupropion	2.94	.086	.65	.40, 1.06	2.01	.16	.77	.54, 1.10	1.09	.30	.83	.59, 1.18	.38	.54	.89	.62, 1.28
**Combination therapies relative to each other**																
Patch + Lozenge vs. Bupropion + Lozenge	5.77	.016	.51	.29, .88	2.00	.16	.78	.55, 1.10	.53	.47	.88	.63, 1.24	2.68	.102	.74	.52, 1.06

* $p < .005$, Bonferroni-corrected for 11 comparisons with $\alpha = .05$

NIH-PA Author Manuscript
NIH-PA Author Manuscript
NIH-PA Author Manuscript

Table 4

Number (%) of adverse events by treatment condition

	Placebo (n = 189)	Bupropion (n = 262)	Lozenge (n = 260)	Patch (n = 264)	Bupropion + Lozenge (n = 267)	Patch + Lozenge (n = 262)
Nausea	16 (4.4)	20 (3.8)	44 (7.8)	25 (4.3)	33 (5.0)	55 (7.9)
Skin irritation	10 (2.7)	14 (2.7)	3 (1.0)	86 (14.7)	14 (2.1)	62 (8.9)
Dizziness	6 (1.7)	6 (1.1)	5 (1.0)	7 (1.2)	15 (2.3)	9 (1.3)
Diarrhea	4 (1.1)	8 (1.5)	13 (2.3)	8 (1.4)	11 (1.7)	6 (1.0)
Vomiting	4 (1.1)	10 (1.9)	8 (1.4)	4 (1.0)	9 (1.4)	11 (1.6)
Dry mouth	3 (1.0)	20 (3.8)	0 (0.0)	1 (0.2)	25 (3.8)	2 (0.2)
Mouth/throat irritation	12 (3.3)	11 (2.1)	38 (6.7)	11 (1.9)	15 (2.3)	40 (5.7)
Alteration of taste	2 (1.0)	8 (1.5)	1 (0.2)	0 (0.0)	9 (1.4)	0 (0.0)
Sleep disturbance and abnormal dreams	20 (5.6)	88 (16.8)	18 (3.2)	66 (11.3)	69 (10.6)	63 (9.0)
Flatulence/gas	5 (1.4)	1 (0.2)	16 (2.8)	0 (0.0)	28 (4.3)	15 (2.1)
Hiccups	1 (0.3)	0 (0.0)	35 (6.2)	0 (0.0)	7 (1.1)	22 (3.2)
Headaches	24 (6.7)	23 (4.4)	29 (5.1)	26 (4.4)	30 (4.6)	34 (4.9)
Dyspepsia (heartburn and indigestion)	4 (1.1)	3 (1.0)	1 (0.2)	4 (1.0)	23 (3.5)	25 (3.6)
Total adverse events	359	524	566	585	654	697

NIH-PA Author Manuscript
NIH-PA Author Manuscript
NIH-PA Author Manuscript

Table 5

Serious adverse events (SAE's) reported between 2 weeks pre-quit and 6-months post-quit by treatment condition

**Placebo (n = 189)**	**Bupropion (n = 262)**	**Lozenge (n = 260)**	**Patch (n = 264)**	**Bupropion + Lozenge (n = 267)**	**Patch + Lozenge (n = 262)**
• Hospitalized for chest pain	• Hospitalized after being hit by a car	• Hospitalized for breathing difficulty	• Hospitalized for treatment of a blood clot in leg	• Appendectomy	• Hospitalized for cataract surgery
• Hospitalized for cardiac arrhythmia	• Hospitalized for chest pain and transient increase in blood pressure	• Surgery to place stents	• Hospitalized for heart attack	• Hospitalized due to breast infection	• Inpatient tonsillectomy
• Hospitalized for bronchitis	• Medication blind was broken at request of MD; no other info available	• Vein transplant surgery related to dialysis	• Hospitalized because of auto accident	• Hospitalized due to falling	• Inpatient hysterectomy because of ovarian tumor
• Hospitalized for pneumonia	• Hospitalized for abdominal pain	• Hospitalized for CAD and TIA	• Hospitalized for mild heart attack	• Inpatient carpal tunnel surgery	• Hospitalized for pneumonia
• Hospitalized for respiratory infection	• Inpatient treatment for alcohol dependence			• Death due to COPD and coronary artery disease	• Hospitalized for stress-related chest pain
	• Hospitalized for pneumonia			• Hospitalized for seizures*	• Hospitalized for lung infection
	• Hospitalized for shortness of breath and heart problems				

* The only SAE labeled as possibly related to study medication. Seizures are an identified potential side effect of bupropion, which the participant was taking at the time of the seizure.
