# Supplementary material for: Effects of Smoking and Cessation on Subclinical Arterial Disease: A Substudy of a Randomized Controlled Trial
Source: PLoS One. 2012 Apr 9;7(4):e35332. doi: 10.1371/journal.pone.0035332 (PMC3322167; doi:10.1371/journal.pone.0035332)
Supplement: Protocol S1 — Trial Protocol. (DOC) [file pone.0035332.s003.doc]

**Natural History of Smoking & Quitting: Longterm Outcomes**

**A. Specific Aims**

At present, some 50 million Americans smoke, these individuals are routinely encouraged to quit, and millions try to quit each year. A great deal is known about how quitting smoking affects a limited set of outcomes. For instance, it is well known that cessation affects the likelihood of lung cancer and heart disease. However, our knowledge of the effects of cessation is narrow. There are major gaps in our understanding of how cessation affects disease processes, mental health, psychosocial adjustment and life satisfaction. Also, little is known about the potential costs or adverse impacts of cessation. Finally, little is known about the precipitants of late relapse. The proposed research is aimed at addressing these gaps in our knowledge. Our cardinal goals are:

*1. To determine the effects of cessation and continued smoking on physiological mechanisms of cardiovascular disease*. We will use state-of-the-art assays such as carotid intima-media thickness, brachial artery endothelial function, and advanced lipoprotein testing to identify the mechanisms via which cessation leads to reduction of cardiovascular disease. We hypothesize that cessation will be followed by an arrest or reversal of atherosclerosis, but that this benefit will also be influenced by other modifiable risk factors (e.g., weight gain, diet, sedentariness, high stress). Our transdisciplinary assessment plan will allow us to produce new knowledge on the impact of cessation on atherosclerosis, and how cessation exerts its effects.^1-8^

*2. To determine the relation between cessation and mental health outcomes*. While cessation may exacerbate psychiatric symptoms in the short run (e.g., increased depression in the first year post-quit) we hypothesize that, over time, it will lead to decreased symptoms and a reduced likelihood of psychiatric diagnosis. These benefits may occur because smoking per se is a stressor^9^ because opponent-processes oppose the euphorogenic effects of nicotine^10^ or for other reasons. In sum we believe that our data will show that quitting smoking is the best thing that people can do to improve their mental health.

*3. To assess the impact of cessation on quality of life (QOL).* We hypothesize that cessation will significantly benefit QOL, but that the extent of benefit will be highly variable across individuals. Most will report enhanced QOL, but a minority will report reductions (e.g., weight gain,^11^ prolonged depression and/or chronic craving could trump the benefits of quitting).

*4. To identify factors associated with late-occurring relapse.* We hypothesize that late relapse (>1 yr postquit) will be related to presence of tobacco in the person’s social network and to lifestyle factors (e.g., a spouse who smokes, time in smokefree environments, alcohol use). We also hypothesize that late relapse will be unrelated to factors that typically predict short-term relapse (e.g., nicotine dependence, withdrawal symptoms, stressors). While much is known about early relapse, little is known about subsequent relapse. Such late relapse has great public health significance since about 1/3^rd^ ^12-14^ of those who abstain for one year eventually relapse.

*5. To characterize better the long-term interdependence between smoking and alcohol use and their relations with important health and adjustment domains.* We hypothesize that there will be a relation between smoking and drinking after a quit attempt with heavier drinking predicting a heightened tendency to relapse. We also hypothesize that successful smoking cessation will produce a reduction in drinking frequency. These hypotheses are based on the recognition that tobacco use and alcohol use often co-occur within contexts and within social groups, and the pharmacologic effects of alcohol may foster behavioral dysregulation (e.g., temporal discounting, reduction of executive control, priming of incentive systems).^15,16^

These goals are part of a research program that has three overarching aims: (1) To provide a broader, more complete view of the long-term effects of quitting and quitting failure than has been available thus far; (2) To reveal the extent to which other modifiable risk factors such as drinking, weight gain, diet and exercise contribute to observed outcomes; and (3) To identify which smokers are most likely to develop positive and negative outcomes. Such information should prompt greater quit rates and ensure that each smoker enjoys the full benefits of quitting. In addition, this research will identify individual difference variables (e.g., genotypes) and other modifiable risk factors that predict important physical health, mental health, smoking, and drinking outcomes. This information should be of great benefit in devising preventive interventions.

**B. Background and Significance**

This proposal is intended to reveal how successful, and unsuccessful attempts to quit smoking are related to important physiologic and psychosocial outcomes, and how such outcomes may also reflect the influence of individual differences and other modifiable risk factors. As a field we have gathered a tremendous amount of data on how various treatments affect cessation rates. However, we have largely neglected the question of how cessation affects people’s lives and functional status outside of achieving abstinence. Moreover, our window of inquiry is typically limited to one year or less, leaving unknown the long-term consequences of a quit attempt. We also have largely neglected the question of how cessation *failure,* and consequent continuation of smoking, affects individuals across broad life domains.

This proposal addresses the natural histories of both the successful quitter and the relapsed smoker focusing on several key questions. **First,** what are the different long-term, physiological, medical, and psychosocial fates of those who are successful and unsuccessful in quitting? **Second**, how do other modifiable risk factors contribute to these outcomes? **Third**, are there individual differences, especially genetic characteristics, that correlate with the important physiologic and psychosocial outcomes under study? This proposal is not intended merely to characterize important outcomes associated with quitting or not quitting. Rather, it is intended to use a transdisciplinary approach to tobacco science to explore processes or mechanisms responsible for important long-term physiologic and psychosocial endpoints of a quit attempt, and to determine how these endpoints and associated processes are interrelated. The key measures that will be used to assess these processes and mechanisms are listed in Table 1.

There is a compelling rationale for exploring the long-term outcomes associated with cessation and cessation failure. Millions of smokers are encouraged to stop smoking each year. Yet, little is known about how the quit attempt, whether successful or unsuccessful, is associated with later events in people’s lives. Whether tobacco cessation should be recommended is not a question of this research. The data documenting the extraordinary burden of illness and death resulting from tobacco use is indisputable. Rather, this research is intended to provide a more comprehensive and fundamental understanding of both cessation and relapse so that smokers can achieve greater quitting success and achieve maximal long-term benefits from quit attempts.

We believe that these questions can be answered only in a transdisciplinary, inclusive manner. This is because smoking is impressively “sticky”; tobacco use is associated with diverse interrelated behaviors and physiologic processes that can be captured adequately only via a synthesis of data derived from diverse but complementary assessment methods that are generated by distinct scientific disciplines and perspectives. In the next section we summarize what is already known about life after a quit attempt.

**Life After a Quit Attempt - Physiologic Outcomes**

An overwhelming body of research shows that tobacco kills more Americans than any other preventable cause.^17^ While some ultimate health impacts of tobacco use have been well documented, relatively little is known about the impacts of cessation or continued smoking on other specific physiologic outcomes such as atherosclerotic progression. This knowledge deficit stems, in part, from a failure to use state-of-the-art assessments from different fields, and then integrate the resultant information. Newer assessments such as carotid intima-media thickness measures (Carotid IMT)^1,18-20^ that quantify real-time risk for adverse atherosclerotic outcomes such as heart attack and stroke^21-33^ can help answer these questions.

*Atherosclerosis and cardiovascular disease.* Tobacco use is a major modifiable risk factor for atherosclerosis,^34-37^ the underlying pathological process leading to myocardial infarction and stroke, the first and third leading causes of death in America. Almost half of the 440,000 deaths caused by tobacco use each year in the United States are the result of cardiovascular diseases.^17^ However, atherosclerosis has other known risk factors that may be influenced by a quit attempt. These include weight-gain induced type II diabetes mellitus, dyslipidemia, hypertension, and physical inactivity.^38,39^ To assess sensitively the global physiologic risk trajectory for atherosclerosis among smokers and quitters, such individuals must be followed in a prospective, long-term way with periodic assessments across diverse modifiable risk domains including blood pressure, lipid status, hyperglycemia, weight, exercise, diet, and others.

Importantly, we now have highly quantifiable measures (e.g., Carotid IMT) that can, in an accurate and reproducible manner, document atherosclerotic progression or regression. These measures document the anatomical basis for the cardiovascular benefits of smoking cessation reported in epidemiologic studies.^40-42^ Such measures will also permit insight into the mechanisms of health outcomes, interactions among risk factors, and the subpopulations most affected by particular outcomes. Specifically, we need to know more about how cessation’s relation to atherosclerosis is influenced by individual differences such as gender, level of nicotine dependence and genotype^43^ and how cessation’s impact is related to changes in other modifiable risk factors such as diet, weight gain, exposure to environmental tobacco smoke, and exercise, which themselves may be affected by cessation.^44^ For instance, cessation induced weight gain might increase the risk of hypertension, insulin resistance, and hyperlipidemia, all important risk factors for atherosclerosis. Some data do address the impacts of such risk factors,^45^ but most data in this area are from cross-sectional or short-term cohort studies.^1,21,29-32,46,47^ Thus, measures such as Carotid IMT and flow-mediated vasodilatation of the brachial artery have rarely been studied prospectively with rich measures of individual differences and other risk factors.

While earlier data on cessation’s impact on carotid IMT showed *arrest* of carotid IMT over three years,^1^ we believe that more careful assessment of other risk factors may reveal *regression*. Such data have the potential to induce clinicians to change fundamentally their practice patterns. For example, data documenting the arrest of atherosclerotic progression resulting from hyperlipidemia has been associated with an explosive increase in the rate of prescribing statin agents.^31^ If tobacco cessation produces similar results, clinicians may be motivated to treat tobacco dependence more consistently and intensively. Of course, such data may also motivate more smokers to quit – pictures of atherosclerotic pathology and its arrest due to cessation could powerfully affect smokers’ motivation to quit.^48^ By integrating prospectively data on changes in smoking habits as well as other key risk factors and objective measures of atherosclerosis, we hope to clarify mechanisms and identify those at greatest risk.

*Metabolic/Anthropometric data.* Research has documented that smokers tend to weigh less than non-smokers and gain weight when they quit, and recent research shows that the weight gain is greater than once thought, averaging 10-20 lbs depending on gender and time since quitting.^11,49,50^ The relative risk of major weight gain has been estimated as 5.8 comparing women who quit with those continuing to smoke.^51^ Most post-quit weight gain occurs in the first three years after quitting.^11^ For both sexes, African Americans, people under age 55, and heavy smokers (25+ cigarettes/day) are at elevated risk for major weight gain.^49,51-58^

Smoking cessation is also associated with increased risk of central adiposity^59^ which places individuals at increased risk of metabolic syndrome, a major risk factor for adverse cardiovascular outcomes. According to the National Cholesterol Education Program Adult Treatment Panel III, patients are diagnosed with Metabolic Syndrome if they fulfill 3 of 5 following factors: waist circumference, triglycerides, HDL-C, blood pressure, and fasting plasma glucose.^60^ Metabolic syndrome and visceral obesity are associated with many other metabolic abnormalities that contribute to cardiovascular disease, including insulin resistance, glucose intolerance, hypertension, abnormal lipoprotein densities, and a prothrombotic state. In cross-sectional studies, smoking itself is associated with the deposition of visceral fat, or central adiposity, and smoking cessation is linked to further central adiposity.^61^

Little is known regarding the progression of central adiposity and the development of the metabolic syndrome in cohorts of smokers and quitters who are followed prospectively, particularly for more than one year. Moreover, little is known about how these metabolic outcomes of cessation are related to individual differences (e.g., nicotine dependence) and other modifiable risk factors. Routine measurement of waist circumference at the iliac crest among smokers and quitters would provide information on the prospective risk of the metabolic syndrome. Among successful quitters, this risk can be assessed in the context of the beneficial metabolic effects associated with smoking cessation including improved glucose tolerance and lipid profile.^59^

**Life After The Quit Attempt - Lifestyle**

*Physical activity & fitness*. Cross-sectionally, both male and female smokers report lower levels of leisure-time physical activity and exercise compared to non-smokers.^62-65^ Former smokers typically have leisure-time activity levels closer to those of never smokers, than current smokers.^63,65,66^ Conversely, current smokers tend to have higher levels of occupational activity,^63,67^ which may be due, in part, to socioeconomic status. Moreover, there is evidence that women who quit smoking adopt a more active lifestyle.^68^ Additionally, even modest amounts of exercise appear to reduce post-quit weight gain^69^ and enhance functional capacity.^70^ These findings suggest that modest amounts of exercise may mitigate potential adverse effects of cessation. Collectively, they suggest that physical activity co-varies with smoking status, and that exercise may be beneficial, at least in women, during quit attempts. Lacking is similar data in men, as well as an understanding of the mechanism by which activity, cessation, and physiological processes lead to vital health outcomes.

Cardiorespiratory fitness is lower in smokers than in non-smokers.^71-76^ This association is mediated by acute and chronic physiological effects of smoking and a tendency for smokers to be less physically active than non-smokers. However, smoking’s impact on fitness cannot be explained completely by differences in physical activity. Longitudinal data suggest that cardiorespiratory fitness recovers following cessation, and that recovery is greater among men than among women.^75,77^ Chronotropic incompetence, or an attenuated heart rate response to exercise, also has been associated with smoking among otherwise healthy adults^78-80^ and may identify smokers with a particularly high mortality rate.^80^ Blunted heart rate responses have also been identified in young adult smokers undergoing symptom-limited exercise testing/^81^ Thus, smoking and smoking cessation *appear* to be significantly related to physical activity and cardiovascular fitness, but the potentially complex interplay of smoking/cessation, weight gain, physical activity, and fitness has not been characterized.

*Diet.* Diet may be influenced by smoking and smoking cessation and may serve as an explanatory variable for other outcomes associated with cessation, such as changes in blood lipids, glucose control, endothelial dysfunction, and Carotid IMT. Much research has assessed the short-term effects of smoking cessation on diet,^82,83^ however, most such data are limited to 6 months post-quit. It is unclear whether there are long-term impacts of quitting on diet, and how quitting and diet operate together to influence health endpoints. While short-term dietary effects may reflect body weight set-point perturbations,^82^ long-term effects could reflect changes in response to weight gain or greater health consciousness among quitters.

**Life After The Quit Attempt - Psychosocial Status**

*Psychiatric symptoms & comorbidity.* The evidence is compelling that smokers have higher rates of psychiatric comorbidities than do nonsmokers. Data show that psychiatric disorders such as depression, anxiety disorders, and antisocial personality disorder are more prevalent among smokers than nonsmokers.^84-87^ However, many unanswered questions remain about this association. For instance, does quitting smoking reduce or increase the likelihood that a person will experience a psychiatric disorder over the long-term?

Some data suggest that quitting can exacerbate psychiatric comorbidities. About 5-7% of smokers experience a new bout of depression within a few months of a quit attempt.^88-91^ This is a high rate of depression considering the short timeframe (and a major depression new-incidence over 12 months of about 1-3%.^92,93^ Other data suggest that psychiatric symptoms and comorbidities may *decline* due to cessation. For instance, some cross-sectional studies^94-96^ have found that cessation is related to improved perceptions of mental health and mental health quality of life. In a rare longitudinal study in this area, Mino et al.^97^ found sizable decreases in psychiatric symptoms over 1 year in a very small sample of quitters (n = 18).

Perhaps quitting has been associated with both decreases and increases in symptoms because cessation produces both an initial increase in symptoms, and also a subsequent and sustained decline. Also, it may be that different outcomes are found in different subgroups of individuals (e.g., those with a prior history of depression.^88,91^ Only a large sample longitudinal study that collects diagnostic information as well as broader mental health information, both before and after cessation, will be able to address adequately the mental health consequences of quitting and failing to quit. Further knowledge gaps include little evidence on the long-term impact of quit attempts on psychiatric comorbidities/symptoms other than depression (e.g., anxiety symptoms; although cf. Breslau^98^) and whether mental health outcomes associated with quitting reflect quitting per se versus other changes associated with quitting (e.g., physical health status, alcohol consumption).

*Alcohol use.*  There is a well-documented dose-response relation between smoking and drinking: heavy smoking is associated with heavy drinking.^99-101^ In fact, while only 10% or less of the general population qualifies for the diagnosis of alcohol abuse or dependence, the figure stands at 30% among smokers.^102^ There is also evidence that alcohol use may interfere with the ability to quit smoking or stay quit. For instance, heavy drinkers are less likely to try to quit smoking and are less successful when they do try.^103^ In general, cross-sectional data suggest a strong link between smoking and drinking.^101,104^ There are also data suggesting linkages between the two behaviors within individuals, across time.^105,106^ However, conflicting data show little relation across time between cessation of one substance and intake of the other.^105-110^

The relation between drinking and smoking remains unclear for a variety of reasons. For instance, smoking status was not biochemically confirmed in many relevant studies. In addition, drinking measures often do not target heavy drinking, problems from drinking, or formal diagnoses of abuse and dependence. Finally, as McClure et al.^101^ acknowledged, in some of the studies, the retrospective nature of some of the measures, makes it unclear whether quitting smoking precedes a decrease in drinking, or vice versa. In addition, some studies comprise very few data points and a small number of quitters.^109^ Moreover, if smoking and drinking are highly correlated across people, but not across time within the same person,^109^ it is likely that the association is produced by common risk factors such as genes or personality factors (e.g., behavioral undercontrol;^111^ also see Bierut et al.^112^ for evidence of a common genetic diathesis). The proposed research would provide an ideal opportunity to explore such common risk factors. There is a need for high quality longitudinal data that can further clarify how and why these two behaviors are interrelated, and how they contribute to other important health outcomes (e.g., health QOL, mental health outcomes).

*Social relationships*. Relatively little is known about how quitting smoking affects social relationships (e.g., marital satisfaction, or satisfaction with, or structure of, a person’s social network^113^). For example, it is not known whether quitting smoking changes the nature of social networks or satisfaction with such networks. Does quitting, in fact, cause smokers to lose smoking friends, or does a smoker’s quitting cause friends to be more likely to quit themselves? An important and related question is whether cessation has long-lasting effects on marital/partner relationships. To the extent that quitting affects mental health status (or stress level) it may indirectly affect marital/partner relationships. Also, while it is clear that having a smoking spouse makes it more likely that a person will relapse, it is unclear whether one spouse successfully quitting increases the likelihood that his/her partner will quit. Finally, it is important to assess social relationship factors because they can play such a strong role in influencing risk and progression for other disorders. For instance, supportive relationships such as marriage are associated with longer life and less severe disease.^114-116^ Many of these questions can be best addressed through a prospective study of social networks.

*Stress.* Some information shows that although smokers report more stress than nonsmokers, their stress levels fall once they quit smoking.^117,118^ Chassin et al.^119^ examined self-reports of stress among those who successfully quit smoking (n = 88) in a cohort study. In comparison to continuing smokers, quitters showed decreased stress from pre- to post-quit. Cessation may reduce stress because smoking is associated with social stigma, opponent-processes that may produce acute stress,^9,120^ worries about disease, and the prospect of having to quit.

The Chassin et al.^119^ study yielded important data that might be very useful in encouraging more smokers to try to quit. However, that study did not address some important questions. For instance, are there gender differences in reported declines in stress? (The number of successful quitters did not permit evaluation of this issue.) Also, perceived stress was measured with only a composite, retrospective stress variable rated with respect to the past year. We have no idea how this is related to actual stress or stressors experienced in daily life, and we don’t know *why* quitters might have reported less stress. Thus, we do not know if quitters reported less stress because they actually encountered fewer stressors, or because of declines in psychiatric symptoms or physical disease. In short, there are intriguing *suggestions* that quitters lead less stressful lives than those who continue smoking - - but we don’t know why this occurs or what its consequences might be.

*Quality of life*. Quality of life (QOL) refers to a person’s net appraisal of the positive and negative aspects of life with respect to such important domains as health, physical functioning, emotional functioning, and vitality. Very few studies have assessed quality of life as a function of quitting, and most were cross-sectional. This is unfortunate since QOL is a valuable, global measure of life satisfaction. Evidence currently shows that smoking is related to poor QOL and that exsmokers report QOL that is similar to that of nonsmokers.^121,122^ For instance, Mulder et al.^94^ performed a cross-sectional study in a Dutch population aged 20-59. They reported that current smokers reported lower QOL than ex- or never-smokers. Differences were especially pronounced on QOL domains related to mental health. This finding agrees with other cross-sectional research showing ex-smokers having greater perceived mental health than continuing smokers.^95,97^

Previous research shows that smokers, when compared to nonsmokers and ex-smokers, have significantly higher rates of psychiatric co-morbidity, higher stress levels, and worse QOL - - especially mental health QOL.^94,121^ Moreover, some evidence suggests that smoking causes or exacerbates perceived stress and mental disorders, and that quitting alleviates these effects.^84,117^ This suggests that QOL improvements may be secondary to improvements in psychiatric symptomatology; i.e., smokers can meaningfully reduce feelings of worry, anxiety, and sadness by quitting. Such an effect could have considerable public health significance, and could motivate a greater number of smokers to try to quit. It would be of great importance to be able to assert that quitting smoking is the single most important thing that smokers can do, not only for their physical health, but for their mental health as well.

Unfortunately, limitations in current research preclude strong conclusions. For example, much of the research is cross-sectional and thus cannot reveal the directionality of the relation between quitting success and QOL; viz. are those with the best QOL more able to quit, or does quitting promote better QOL? Also, this research does not reveal which smokers do *not* experience QOL enhancements after quitting (e.g., the most dependent or those who gain weight?).

**Life After The Quit Attempt – Smoking, Dependence, and Delayed Relapse**

*Smoking and dependence-related phenomena*. It is important to determine how quitting affects phenomena related to dependence. For instance, how quickly do smoking motives, urges, and withdrawal symptoms decline after quitting? Smokers contemplating quitting often report stories of urges and relapse after years of abstinence. It is important to determine the trajectories of urges long after quitting. The little research in this area suggests that smoking-related beliefs change as a function of successful cessation. For instance, successful quitters show decreases in smoking motives.^117^ However, little or no research has been conducted on this issue beyond one year post-quit, and it is unknown whether such attitudinal changes are related to late relapse.

*Late relapse*. The proposed research will provide new information on the predictors of late relapse (> 1yr post-quit). Among former smokers who have been abstinent for a year, about 1/3^rd^ eventually relapse.^12-14^ We know very little about what causes such delayed relapse and the proposed research will help us identify factors associated with it and whether these factors are distinct from those associated with “early” relapse. Such information should be critical to developing new, effective interventions to prevent late relapse.

Early relapse is associated with severe withdrawal, heightened negative affect, early morning craving, and environmental stressors.^123-126^ However, there is reason to believe that long-term relapse is precipitated by different factors. In our research we discovered that early relapse was associated with elevated craving.^127^ However, later relapse (> 3 months post-quit) was associated with the density of smokers in an individual’s environment. Other research also supports interpersonal/social and associative factors in precipitating later relapse.^128^ These findings support the model of relapse articulated previously by our research group^124,125^ that holds that different factors influence relapse vulnerability across the post-quit period.

The present research will address relapse that occurs across a 5-year span (at least 3 years being funded by this application). In one of the few studies on this topic, Killen et al.^129^ explored the predictors of relapse among 420 individuals who had remained abstinent for at least 3 months post-treatment. This research suggested that the development of depression over the first three months post-quit was predictive of later relapse, as were lower BMI gain and higher nicotine dependence among subgroups of subjects. While these findings are of interest, more research is clearly needed because: (1) Some measures were not optimal (e.g., craving was assessed at a single point in time, psychiatric diagnoses were not obtained); (2) No measures were collected post-treatment; and, (3) Only a very narrow range of assessments was used (four variables); and so on. Other research in this area suffers from similar limitations.^130^ After surveying available research in this area Ockene et al.^131^ concluded that very little could be discerned about late relapse from available research because studies typically used only a small number of parochial variables as predictors.

In the present research we will be able to contrast distinct models of late relapse: viz. is late relapse related to: (a) *Initial level of dependence, (b) Withdrawal severity,* (c) *Chronic distress* (e.g., levels of negative affect, psychiatric symptoms/co-morbidity, reduced QOL, fatigue) *(d) Stressful events* *(e) Environmental context & lifestyle* (e.g., alcohol intake, time spent with smokers, sedentariness, weight gain), and

*(f) Overconfidence.*^132,133^ This data will allow us not only to understand the process of relapse, but also, perhaps, to identify those at heightened risk for relapse and aid in the design of effective interventions. For instance, it may be that an effective relapse intervention program should really concentrate not on the abstinent smoker, but instead on his/her environment.

**Summary: Life After The Quit Attempt**

Our review indicates that too little is known about important long-term consequences of quitting and failing to quit smoking (e.g., mental health status, atherosclerosis progression, QOL). In addition, little is known about the roles of other modifiable risk factors in these outcomes, and about how individual differences identify those at greatest risk for outcomes. How are modifiable risk factors associated with one another? Is the likelihood of exercise related to the magnitude of weight gain over the long-term? How are all of these related to important physical outcomes such as central adiposity, diabetes, or atherosclerotic progression? For instance, there is suggestive evidence from a *cross-sectional* study^134^ that physical activity and a healthy diet reduce atherosclerosis, but not among people who smoke; i.e., smoking trumps positive lifestyle factors. It is important to explore such relations in a more inclusive longitudinal study, and determine whether such relations differ by gender, race, or level of nicotine dependence.^43^

Extant studies addressing questions such as those listed above tend to have significant limitations: (1) *Inadequate, brief follow-up*: the follow-up is too brief and often does not include both prequit and postquit measurement; (2) *Inadequate measurement*: measures are often global and impressionistic and convergent measures are rarely used; (3) *Cross-sectional designs:* many studies are cross-sectional rather than longitudinal or prospective in design (e.g., ^94,95,97,135^), making it difficult to determine causal relations and directionality; and (4) *Narrow, parochial focus*: for instance, studies may focus on smoking behaviors and attitudes, but not health and genotype. Or, they may focus on health, but not psychosocial factors. An informed and complete picture of the fate of the quitting smoker can be gleaned only from a synthesis of information and theory across diverse disciplines.

**Individual Differences – Genes and Personality**

*Genotype*. There is a growing literature linking nicotine dependence with genetic variation.^136,137^ The proposed research would add to this literature by examining a rich array of physiologic, addictive, and psychsocial phenotypic measures over an extended period of time and their relations with promising candidate genes. In this research, outcomes will be related to genotype and personality (in addition to other individual differences: gender, psychiatric diagnostic history, nicotine dependence). As discussed more fully in Project 1, genotype will be related to diverse measures of nicotine dependence: e.g., multivariate questionnaire measures,^138^ relapse latency, and withdrawal.^124,125^ In the current project genotypes will be related to measures of dependence over the 3-year study period (e.g., pack-years of smoking/long-term trajectories of smoking). Previous research^136,137,139,140^ suggests targeting candidate genes coding for nicotinic receptors, nicotine metabolism, dopamine synthesis, dopamine re-uptake, dopamine receptors and function, acetylcholine re-uptake & metabolism, and positional candidates. Genotypes will also be related to alcohol use/abuse and comorbidity with smoking (e.g., ^112^). Some candidate genes in these relations will be ADH, ALDH, dopamine, and GABA genes.^141^ Comorbidity of smoking with affective disorder will also be examined. Genotype will also be related to atherosclerotic progression among smokers (e.g., 5A/6A polymorphism of the stromelysin-1 promoter;^142^ leucine7 –to-proline7 polymorphism in preproneuropeptide Y gene signal peptide^143^ & glutathione S-transferase M1;^144^ endotoxin receptor polymorphism CD14^145^).

*Personality*. A considerable body of research links both negative affectivity and disinhibition/behavioral undercontrol with addictive disorders.^111,146^ Therefore, BF/MPQ^147^ Negative Emotionality and Constraint higher order factors will be related to both smoking and alcohol trajectories and outcomes, and analyses will determine whether personality mediates genetic effects on these outcomes.^111^ Negative Emotionality will also be related to measures of atherosclerosis and its progression given the links between negative affectivity and cardiovascular disease.^2,4,8^

**Importance of the Proposed Research to Science and the Public Health**

This research may yield the best long-term information to date on both the physiologic benefits and costs of successful and unsuccessful attempts to quit smoking. It will make important contributions to descriptive psychopathology. It will provide the most fine-grained and accurate information to date on the histories of quitters and relapsers after a quit attempt.

This research will not only characterize the fates of smokers who are successful and unsuccessful in a quit attempt, but will also shed light on mechanisms that mediate observed outcomes. Observed mediational relations may suggest the importance of comprehensive interventions^148-150^ that can mitigate or prevent negative outcomes of cessation, or enhance positive outcomes. Application of such interventions would be greatly aided by identification of individuals most likely to experience important outcomes of cessation or continued smoking.

This research will also elucidate the precipitants of late relapse - - relapse occurring after the first year of abstinence. Additionally, this research will examine genetic correlates of outcomes such as ability to quit smoking and remain smoke-free, severity of nicotine dependence, severity and patterning of withdrawal symptoms, weight gain, psychiatric diagnoses/symptom profiles, and progression/status of smoking-related disease. Finally, this research may yield information that enhances smokers’ motivation to quit. Smokers may be more likely to make a quit attempt if they believe that smoking cessation will lead to greater life satisfaction, reduced sadness and worry, improved social relations and clinically significant reductions in risk of heart attack and stroke.

It is clear that the information to be gathered is of substantial public health importance. Since 50 million current tobacco users in America are encouraged to quit and approximately 20 million try to quit each year, it is essential to learn how quitting and continued smoking are related to mental health status, disease status and progression, quality of life, and late relapse. It is equally important to be able to identify risk factors for these important outcomes so we can better assist smokers to successfully quit, prevent relapse, and improve mental and physical health.

1. **Preliminary Studies**

The UW-TTURC research team at UW-CTRI has extensive experience in tobacco intervention research. Since 1992, CTRI has conducted more than 20 randomized smoking cessation clinical trials, including trials of pharmacotherapies^151,152^ and psychosocial treatments.^153,154^ Of particular relevance to the current proposal is UW-CTRI’s demonstrated ability to recruit large numbers of smokers for clinical trials. More than 1500 community smokers enrolled in the first UW-TTURC studies, and more than 1500 additional primary care clinic patients enrolled in a cessation effectiveness trial.^153^ Recently, CTRI has demonstrated an ability to recruit minority smokers at meaningful levels; 22% of participants in a combination pharmacotherapy clinical trial were African American,^152^ as were 27% of participants in a primary care effectiveness study.^153^ CTRI has also achieved impressive retention rates in studies. For example, in a clinical trial requiring very extensive assessments 82% of 608 enrolled smokers were successfully followed up for one-year.^152^

Recent research emerging from CTRI reflects significant methodological advances. For example, UW-CTRI researchers have adopted state-of-the-art psychophysiological (e.g., electromyography)^127^ and real-time self-report assessments (e.g., palmtop computers for ecological momentary assessment)^155^ to sensitively detect changes associated with cessation. With regards to assessment, the research team has developed and validated the WISDM-68, a multidimensional research measure of smoking dependence motives that predicts heavy use, withdrawal and relapse.^138^ The WI-PRISM is a brief clinical measure of relapse proneness that appears useful for treatment matching, as well as predicting early and late relapse as well or better than other dependence scales.^156^ The WSWS^157^ is a 28-item multi-dimensional measure of nicotine withdrawal containing a craving scale that was found to mediate bupropion and counseling effects in our first TTURC research.

Researchers at CTRI have also demonstrated an ability to analyze data from clinical trials in novel, sophisticated ways. For example, researchers have published papers on innovative ways (e.g., hierarchical linear modeling) to analyze nicotine withdrawal symptom data. These analyses have revealed that withdrawal experiences are not as transient as previously thought and that symptom ratings over time can be parsed into multiple dimensions (e.g., elevation, trajectory, volatility) that significantly and independently predict relapse.^124,125,155,158^ Researchers at CTRI recently conducted mediational analyses using data derived from individual withdrawal symptom growth curves. Results suggest that the effects of bupropion and individual counseling on cessation success are partially mediated by reductions in the severity of cigarette cravings and changes in the linear slope in craving ratings over time. Past CTRI research has also identified moderators of treatment effects that may be included in a treatment assignment algorithm developed through the proposed TTURC research. Our research showed that women and persons with a history of depression are particularly aided by bupropion as opposed to NRT.^159^

During initial TTURC funding, CTRI researchers established links with the University of Utah Huntsman Cancer Institute. Wisconsin has sent over 1100 DNA samples of smokers and community controls to Utah. All samples have undergone quality control at Utah and have been diluted to 200 mg/ml, to facilitate subsequent DNA sequencing and SNP genotyping. After identification of common haplotypes in the nicotinic subunits in a subset of these smokers and controls, we will identify tagged SNPs and genotype them in the remaining samples. A total of 192 samples from smokers and nonsmokers (two trays of 96) have been defined. Amplification and sequencing has begun on the first tray of 96 samples for the CHRNA3 and CHRNB4 genes.

In addition to the experience in conducting tobacco research, the UW-CTRI has a long history of linking with researchers across the UW campus and beyond to address vital tobacco science and policy challenges. The proposed UW-TTURC, and specifically this research project will advance our scientific collaboration to a new level bringing together a team of co-investigators each with considerable relevant expertise from diverse disciplines. Some of our collaborators in the proposed research are:

**Lisa Colbert, PhD, MPH** is a kinesiologist with expert knowledge in the assessment of physical activity and exercise. She has conducted extensive research on the relationship between physical activity and health outcomes, including longitudinal research^160,161^ and research with smokers.^162-164^

**Michael Fleming, MD, MPH** is a nationally recognized leader in the assessment and treatment of alcoholism and alcohol abuse.^165-170^ In addition, he has expertise in the assessment of alcohol use in longitudinal research.^168,171^

**Patrick McBride, MD, MPH** is an expert in all areas of preventive cardiology, especially cholesterol, exercise, and exercise testing.^172-174^ He has served on the NCEP ATP III, AACVPR Cardiac Rehabilitation, and AHA Preventive Cardiology for Women Expert Panels. He has also conducted research on the role of lipoproteins and emerging risk factors in atherosclerosis.^175-178^

**F. Javier Nieto, MD, PhD** is a physician epidemiologist with expertise in carotid ultrasound measures of artherosclerotic progression^179^ and the study of endothelial function.^180^ He has also examined racial disparities in health outcomes^181^ and served on quality control committees for large-scale studies.^182^

**Linda Roberts, PhD** has great expertise in social network, social mapping and couples research.^183,184^ She also has expertise in how social relationships are affected by substance use (especially alcohol).^183,185-187^.

**James Stein, MD** is an expert in preventive cardiology, with particular knowledge of the ultrasound assessment of atherosclerosis^188^ and the study of endothelial function.^189,190^ He has pioneered work using carotid intima-media thickness measures to determine “cardiovascular age” and predict cardiovascular risk.^189,191,192^

**Walter Willett, MD, DrPH** is a researcher of international stature who has expertise in the assessment of diet and nutrition and their relations with other modifiable risk factors and important health outcomes.^193-196^ In addition, he has significant expertise in longitudinal research methods.^197-201^

Finally, UW-CTRI researchers have made significant theoretical contributions to the tobacco control field. During the first UW-TTURC, researchers developed a sophisticated model of addiction motivation with heuristic value,^120^ proposed a data-based model for the study of relapse proneness,^133^ and contributed to the development of a model for the transdisciplinary evaluation of tobacco dependence treatments.^202^

**D. Research Design And Methods**

**Recruitment and Enrollment**

*Recruitment.* Subjects in the current study will be recruited from participants from Project 1: Efficacy. To provide the reader with necessary context, we will describe the randomization plan and cessation treatments used in that study (Figure 1). Each pharmacotherapy will be used in a manner consistent with recommendations from the PHS Guideline, *Treating Tobacco Use and Dependence*^203^ or with package insert instructions (lozenge). Pharmacotherapy for this study will be made available as an unrestricted gift from GlaxoSmithKline to the University of Wisconsin contingent on NIH funding. In addition to pharmacotherapy, subjects will receive brief in-person counseling (4 sessions).

Participants who volunteer for Project 1 will simultaneously volunteer for this follow-up study (Project 2: Longterm Outcomes) that is intended to last five years (although only three years will be funded by this TTURC application). During recruitment, prospective subjects will be told of the long-term nature of the study, and will be given explicit information on study requirements. Researchers will stress the vital importance of a firm commitment to participate in the entirety of the study (although we will note their right to withdraw at any point). Subjects will be told that the study has two aims: (1) to determine which medicines are most helpful to smokers trying to quit, and (2) to study the long-term effects of quitting and not quitting on health, behavior, and life satisfaction. They will be told that it is as vital for everyone to remain in the study regardless of success. In addition, subjects will be fully informed of the potential risks and benefits of participation and will be provided appropriate HIPAA information. Thus, this project is described in a separate application from Project 1, the two projects will share the same subjects.

Individuals may be included who: report smoking at least 10 cigarettes/day for the previous six months, produce a breath sample with a CO level > 9 ppm, report being motivated to quit, and are able to read and write English. Medical screening at baseline will ensure that participants can safely be prescribed any of the included medications (e.g., no uncontrolled hypertension, history of bipolar illness, recent myocardial infarction, current alcohol dependence with risk of seizure, seizure history, history of bulimia or anorexia nervosa, or current use of a MAO inhibitor). In addition, any individual who reports regular thoughts of suicide or self-harm will be assessed by a clinical psychologist, and those judged to be at risk for suicide will be excluded and offered referrals to appropriate treatment resources.

Figure 1: Treatments for the 1,520 study participants randomized to Project 1: Efficacy For the purposes of this grant application, participants will be considered part of Project 1: Efficacy from the time of their enrollment and completion of pre-quit assessments until their one-year post-quit anniversary date. They will be considered part of the current project (Project 2: Longterm Outcomes) from their one-year post-quit anniversary date until at least three years after the quit attempt anniversary date. Enrollment for Project 1 will take place over the first two years of this five-year TTURC grant. Enrollment in Project 1 will commence about six months after the start of grant funding and will continue for approximately 18 months.

*Enrollment*. Enrollment for Project 2 (Longterm Outcomes) will begin at around 18 months (when the first participant in Project 1 reaches his/her 12 month quit date anniversary) and continues through month 36 (when the last participant in Project 1 reaches his/her 12 month quit date anniversary). The end of Project 1 and the start of Project 2 are designed to be seamless for participants. Based upon cost considerations and power, we will invite only 900 Subjects from Project 1 to participate in Project 2.

Participants will be enrolled in Project 2 in the following manner. All Project 1 participants will be contacted by telephone around 12 months after the quit attempt and queried regarding their smoking status. At that 12 month phone contact, all abstinent individuals (estimate = 360, see “expected abstinence rates and attrition” below) will be enrolled in Project 2 and scheduled for the one-year, post-quit in-person assessments. Additionally, we will enroll relapsers until approximately 540 have been scheduled for the one-year in-person assessments. The estimated 360 abstinent and 540 continuing smokers will serve as the total cohort (N=900) to be followed for two additional years in Project 2. These figures are inexact since they depend on abstinence rates. Subject enrollment will continue until the 900 sample size target is met. Selection of relapsers for Project 2 will be random with one constraint: ethnic minorities will be over-recruited since evidence suggests that these individuals tend to attrit at a higher rate than other subjects^204^. Project 2 is designed so that all essential physiologic and psychosocial outcomes are collected at the two-year follow-up point (three years from the quit date).

Additional details of our recruitment plan are in Project 1 and will be based on our successful recruiting strategies used during the first UW-TTURC when more than 1,500 participants were enrolled. Participants for this study will be recruited in both Madison and Milwaukee to increase diversity (with at least 50% of subjects recruited in Milwaukee). Based upon our previous research, the overall induction effort will yield the following approximate percentage of subjects: female = 56%, White = 79%, African-American = 17%, and other ethnic minorities = 4%.

**Expected Abstinence Rates and Attrition**

Based on projections detailed in Project 1, we expect the overall biochemically-confirmed one-week point–prevalence abstinence rates to be 27.6% at six months and 23.6% at 12 months. A survey of some dozen clinical trials (e.g., ^151,154,205-207^) shows that a typical decline in abstinence rates from 6 to 12 months is about 4%. A similar adjustment was applied to our expected six-month abstinence rates to yield projected 12-month abstinence rates (See Table 2). Comparisons with actual reported 12 month rates ^151,153,207-211^ shows precedence for these estimates.

*Attrition*. Attrition in Project 2 will be influenced by attrition that occurs during Project 1 (the first year after the quit attempt; see Project 1 for more details). Our attrition expectation for Project 1 suggests one-year attrition rates of 10-20%, based on prior research. For instance, in our most recent clinical trial,^138^ 10% of the sample stopped study participation over the 12-month follow-up period. This study is highly relevant to the current attrition estimate since it involved similar assessment and ethnic representation as is proposed for Project 1. That study, however, did not include additional aggressive steps that we will follow to minimize attrition in the current research (see “Steps to reduce attrition” below). In two of our other recent clinical trials having extensive assessment burdens and visit schedules, attrition was about 18% at one year. The attrition projection of 15% over the course of Project 1 therefore, seems reasonable given the retention steps to be employed in the proposed study. Thus, we envision that 15% of 1,520 participants enrolled Project 1 will attrit by month 12, leaving approximately 1,292 available for enrollment in Project 2 (many more than the target enrollment for Project 2 of 900).

Among the 900 subjects enrolled in Project 2, some will attrit over the two-year course of this study. Table 3 summarizes the expected sample size by year for Project 2 based on an initial enrollment of 900 and factoring in expected rates of attrition, additional quitting and relapse that are project to take place over the two year course of the study. Based upon other long-term cohort studies that involve aggressive efforts to maintain subject involvement across multiple years of follow-up, we expect the bulk of attrition (15%) to occur in Year 1.^212,213^ Moreover, we expect to limit attrition over the next two years (Project 2) to 5% per year (see Table 3). These projections are consistent with prior longitudinal follow-up studies involving both smokers^214^ and other subjects.^204,212,215,216^ These estimates suggest that 812 subjects (approximately 90% of the original 900) will be available for analysis at the end of Project 2.

*Steps to reduce attrition*. Attrition will occur not only in the first year (Project 1: Efficacy), but will continue throughout the course of Project 2. Since attrition can have extreme negative impacts on the external and internal validity of a study (producing bias, reducing power), we will take a series of steps to minimize attrition. These steps are supported by considerable research on determinants of attrition in longitudinal studies and will include the following:

- We will obtain an extensive list of family, social network, and employment information that we can use to locate subjects lost to follow-up.^213,216,217^
- At least 10 efforts to contact individuals, using both telephone and mail, will be made to ensure that subjects fully participate in the key follow-up points: telephone (at months 18 and 30) and in-person (at 12, 24 and 36 months). Successful contact strategies will be maintained and used in subsequent efforts to establish contact.
- Each participant will be assigned a UW TTURC staff person who will be the liaison for the participant throughout the entire study period. Previous research shows that a personal relationship with a study liaison is highly important in study retention.^204,215,216^ The contact person will conduct principal assessments in this study with the exception of the smoking outcome questionnaires.
- Research suggests that belief in the importance of the research and the importance of personal participation both affect attrition.^218^ In light of this, we will inform subjects of study progress through a participant newsletter that will be sent to them twice/year, we will send them birthday cards each year acknowledging and thanking them for their participation, and we will send them small gifts labeled with study logos and phone/email information (e.g., pens, refrigerator magnets, notebooks).
- Previous research shows that incentives can promote adherence and continued participation^204^. Therefore, we will use inducements and monetary compensation to recognize subjects’ efforts.
- Allowing considerable personal control over assessment completion has been linked to greater study adherence. Research suggests that nonadherence or reluctance is often a temporary state and that 70% of subjects are willing to participate at some point in time after a refusal.^216,219,220^ In the proposed research we will allow subjects flexibility regarding assessments including “rain checks” and make-up scheduling.
- A significant proportion of our sample will be African-American and some data suggest that they are more likely to attrit than other participants.^212,221^ In order to retain high percentages of African-American participants we will ensure that research staff are culturally sensitive, that assessments are scheduled around work and childcare obligations, and that appointments can be easily rescheduled.^218^

Some of these steps designed to promote adherence might affect some outcomes such as abstinence. We view this possibility as being preferable to high levels of missing data and the bias that would result from a less aggressive retention strategy. Also, we note that some of these steps (e.g., sending a newsletter) will be started in Project 2, so they should not bias abstinence rates in Project 1. Contact persons will be extensively trained to avoid giving any counseling/advice during contacts, as well as to be nonjudgmental, active listeners, and persistent.^222^ Research shows that when steps such as the above are used, 80 to 97% of participants can be followed effectively over multiple years. This is true even for individuals low in SES, drug users, and those with psychiatric comorbidities.^204,212,214-216^

*Smoking outcomes.* Smoking status will be somewhat fluid over the two years of follow-up for subjects in Project 2 as some participants will transition between smoking and abstinence during this time. Research suggests that between 3 – 5% of abstainers at the end of Year one (end of Project 1) will resume smoking each year over the course of follow-up Years 2 & 3 of Project 2.^13^ Based upon this, we estimate an annual relapse rate of 4% among those confirmed to be successful quitters one year post-quit (See Table 3).

Conversely, data suggest that a significant percentage of those relapsing by Year 1 or later will re-establish abstinence. The large COMMIT longitudinal study suggests a natural quit rate of about 4%/year over several years^223^. These figures agree with those from the Working Well Trial, the Normative Aging Study, and others showing approximate long term 3% to 5% spontaneous quit rate/year.^12,101,224^ Some studies such as the Health and Retirement Study^113^ report higher spontaneous quit rates but the older age range of those participants makes the data less relevant. It is possible that quit rates over Follow-up Years 2 & 3 will be somewhat higher than 4% in this study due to follow-up contacts and the fact that participants entered the study because of an interest in quitting smoking. However, we will use a 4% quit rate as a conservative estimate for both Follow-up Years 2 & 3 (Table 3). We recognize that newly abstinent subjects cannot be treated as continuously abstinent. However, our data will permit continuous measures of smoking over the three-year follow-up period.

Some analyses will use subjects who have been continuously abstinent for the entire 3-year follow-up. Given the abstinence and attrition estimates in Table 3, we expect 300 subjects to fulfill criteria for continuous abstinence at the end of Year 3.

Table 4. Assessment schedules (Orientation visits part of Project 1)

| **Evaluation** | Orientation  (3 Visits) | One Year In-Person Visit | 18-month Phone Follow-Up | Two Year In-Person Visit | 30-month Phone Follow-Up | Three Year In-Person Visit |
| --- | --- | --- | --- | --- | --- | --- |
|
|
| Sign Informed Consent and HIPAA | X |  |  |  |  |  |
| Demographics | X |  |  |  |  |  |
| Smoking History | X |  |  |  |  |  |
| Wisconsin Inventory of Smoking Dependence Motives (WISDM-68) | X | X |  | X |  | X |
| Wisconsin Predicting Relapse in Smoking Measure (WI-PRISM) | X |  |  |  |  |  |
| Fagerström Test of Nicotine Dependence (FTND) | X |  |  |  |  |  |
| Wisconsin Smoking Withdrawal Scale (WSWS) | X | X | X | X | X | X |
| Multidimensional Personality Questionnaire-Short Form (MPQ-S) | X |  |  |  |  |  |
| Positive and Negative Affect Scale (PANAS) | X | X | X | X | X | X |
| Depression Proneness Inventory (DPI) | X |  |  |  |  |  |
| Holmes-Rahe Social Readjustment Rating Scale | X | X |  | X |  | X |
| International Physical Activity Questionnaire (IPAQ) | X | X |  |  |  | X |
| PrimeScreen (dietary questionnaire) | X | X | X | X | X | X |
| Food Frequency Questionnaire (FFQ) | X | X |  | X |  | X |
| Vital Signs^a^ | X | X | X | X | X | X |
| Suicidality Assessment | X | X |  | X |  | X |
| Complete Physical Exam | X |  |  |  |  |  |
| Waist Circumference | X | X |  | X |  | X |
| National Comorbidity Survey – Revised (NCS-R-CIDI)^b^ | X | X |  | X |  | X |
| Short Inventory of Problems (SIP-2R) – Alcohol related problems | X | X | X | X | X | X |
| Social Network Interview | X | X |  | X |  | X |
| Kansas Marital Satisfaction Scale ^c^ | X | X |  | X |  |  |
| Quality of Life Inventory (QOLI) | X | X |  | X |  | X |
| Pregnancy Test for women | X |  |  |  |  |  |
| Ultrasound Carotid Intima–Media Thickness (CIMT) | X |  |  |  |  | X |
| Ultrasound Brachial Artery Response Test (UBART) | X |  |  |  |  |  |
| Exercise Stress Test | X |  |  |  |  | X |
| Pedometer Training and Assessment | X | X |  | X |  | X |
| Blood Draw^d^ | X | X |  |  |  | X |
| Smoking Status^e^ | X | X | X | X | X | X |
| **Time Required For Each Visit (in hours)** | **8** | **4** |  | **2** |  | **4** |

^a^ Vital signs includes carbon monoxide, blood pressure, weight, temperature, pulse, and height at Visit 2. ^b^ NCS-R-CIDI, which should last on average 90 minutes, includes a Screener and the following modules: Depression, Irritable Depression, Panic Disorder, Specific Phobia, Social Phobia, Agoraphobia, Generalized Anxiety Disorder, Suicidality, Use of Services, Alcohol Use, Tobacco Use, Chronic Conditions, Neurasthenia, 30-Day Functioning, 30-Day Symptoms, Obsessive-Compulsive Disorder, Worries and Unhappiness. ^c^ This will be administered to participants who are married or who have a domestic partner. ^d^ Blood will be drawn for the following tests: NMR lipid profile, creatinine, simultaneous fasting blood glucose and insulin, possibly carotenoids, hemoglobin A1C, and high-sensitivity c-reactive protein. ^e^ Smoking status includes a review of the abstinence calendar, nature of relapse contexts, use of cessation aids and new quit attempts.

**Measurement and Data Collection Plan**

For Project 1, baseline questionnaire/interview data as well as physiological assessments will be collected during visits occurring prior to the quit day. This pre-quit data will also provide baseline data for Project 2. In addition to this baseline data, many of these same assessments will be collected at 12, 24, and 36 month (in-person) and 18, 30, and 42 month (telephone) follow-up contacts for Project 2. The timetable for data collected over the three years that comprise Projects 1 and 2 is depicted in Table 4.

*Annual In-Person Clinic Visits (Months 12, 24, and 36).* Participants enrolled in Project 2: Longterm Outcomes will attend an in-person clinic visit at one, two, and three years (12, 24, and 36 months) post-quit attempt. During these visits, participants will repeat many of the baseline assessments (see Table 4) including information to determine continuous and point-prevalence abstinence, the nature of any relapse event (environmental and social context, stressors, alcohol use, and so on), use of any new cessation aids, new quit attempts, and withdrawal and affective status (WSWS, PANAS, etc.). Suicidality will be assessed and appropriate referrals made in the event that suicide risk is determined.

Table 4 also lists other assessments that will take place at each annual in-person visit. Virtually all of the baseline paper-pencil questionnaires will be re-administered as well as the NCS-R-CIDI. In addition, some physiologic measures will be assessed including weight, body mass index, vital signs, waist circumference, and carbon monoxide (provided via a breath sample). Selected physical measures will be collected at some of these visits. For instance, the Carotid IMT, Exercise Stress Test, and NMR LipoProfile tests will be performed at the Baseline and Year 3 Visit. Brachial artery endothelial function will be tested at the Baseline and Year 1. Blood tests will completed at Baseline, Year 1 and Year 3 (but not Year 2) including NMR Lipoprofile, fasting glucose and insulin, hemoglobin A1C, creatinine, high sensitivity C-Reactive Protein, and possibly carotenoids.

The annual in-person clinic “visits” will occur over two days. The first day will involve assessment administration (questionnaires, simple physiologic measures). The second day will involve more complex physiologic measures including, when indicated, carotid IMT, brachial artery endothelial function, exercise treadmill stress test, and blood tests. Each of the two in-person Follow-up Visits days require about 2 hours (except for Year 2 when only a single day 2 hour visit is required). To ease the assessment burden we will take the following steps: (1) participants may interrupt the assessment session to take breaks, (2) there will be snacks to provide energy and a break from responding, (3) questionnaires may be completed at home if requested, and (4) participants may complete the assessments in multiple visits.

Pedometers will be distributed at the Annual Visits (months 12, 24, 36) along with a weekly recording log and a stamped, padded envelope addressed to the research center. Participants will be instructed to record the pedometer reading each night for a week and then return the pedometer by mail. Participants will receive a reminder phone call the day after they receive the pedometer to encourage daily recordings.

Assessments will be completed using the same procedures as at baseline. For instance, the NCS-R-CIDI will be administered in a computer-assisted format. If the participant is tested outside the research site, study personnel will administer it via laptop computer. Only the medical tests mandate an in-person visit. Finally, an additional visit will occur at 60 months after the quit day, but not within the funding period of this award. As in our previous research, responses on all forms will either be entered via computer (with “catch” criteria) at the time of collection or recorded on scannable forms to minimize the need for manual data entry. When scannable forms are used the researcher will inspect each form as it is completed to reduce scanning errors.

*Telephone Clinic Visits (Months 18 and 30).* A follow-up telephone assessment will take place at 18 and 30 months post quit-attempt. At this phone contact, the following factors will be assessed: continuous and seven day point-prevalence tobacco abstinence, the nature of the relapse contexts (environmental context, stress occurrence, use of alcohol, social context and so on), use of any cessation aids, new quit attempts, alcohol use in the last 7 days, tobacco withdrawal symptoms (WSWS), the PrimeScreen qualitative dietary survey, and affective status (PANAS) including suicidality. In total, this telephone assessment is estimated to take between 20 and 30 minutes.

**Measurement and Data Collection Plan - Assessments**

Physiologic

This research will target cardiovascular disease risk as the primary physiologic outcome of interest. This research will utilize state of the art, well-validated technologies and measures to prospectively assess atherosclerosis progression as well overall cardiovascular risk. Three of these measures (ultrasound measurement carotid IMT, brachial artery endothelial function, and advanced lipoprotein testing using NMR spectroscopy) are described below.

*Carotid intima-media thickness (Carotid IMT*). Carotid IMT quantifies sub-clinical atherosclerosis. This measure will be assessed at baseline and at the three year follow-up visit. Carotid IMT has been shown to provide specific predictive value for risk of heart attack and stroke and will be measured using the standardized protocol from the Atherosclerosis Risk in Communities (ARIC) study that optimizes images of the common, bifurcation, and internal segments of each carotid artery.^225^ The carotid arteries will be imaged with an 8.0 MHz linear array ultrasound transducer (8L5, Acuson Sequoia, Siemens Ultrasound). Images will be recorded and stored digitally using a Camtronics Vericis System (Camtronics Medical Systems, Hartland, WI) (Figure 2). The combined thicknesses of the intimal and medial layers of the far walls of each 10 mm carotid artery segment are measured in triplicate. Far wall mean and maximum wall thicknesses of each carotid segment are averaged to define a segmental mean or maximum Carotid IMT score. Composite Carotid IMT_max_ is calculated as the mean of the segmental maximum scores from all measurable segments (maximum of six). Composite Carotid IMT_mean_ is calculated as the mean of the segmental mean scores from all measurable segments (maximum of six).^226,227^ In our laboratory, the coefficient of variation for Carotid IMT_max_ is 4.9% and Carotid IMT_mean_ is 3.2%. These values imply a high degree of reproducibility, as good as or better than reported in major epidemiologic trials.^20,23,225,228^

*Brachial artery reactivity testing (BART):* Endothelial function will be assessed using ultrasound measurement of the brachial artery in response to flow mediated vasodilation (FMD). This dynamic measure of arterial function is impacted immediately by cigarette smoking and is extremely sensitive to rapid changes in risk factors for cardiovascular disease. This will be measured at baseline and at the one-year post-quit attempt visit. Impaired endothelial function is associated with cardiovascular risk factors and prevalent as well as incident cardiovascular disease including among smokers.^229-233^ This association is dose-dependent, both with increasing pack-years of exposure and varying levels of environmental tobacco smoke. It also seems to be potentially reversible as evidenced by the weaker association seen in former smokers.^234^ Further studies suggest a synergism between smoking and hypercholesterolemia,^232^ raising the possibility that smoking potentiates endothelial dysfunction by enhancing LDL oxidation.^235,236^

Brachial artery endothelial function will be measured noninvasively in the morning in the fasting state by a well-validated technique using high-resolution B-mode ultrasound of the brachial artery.^235-239^ Subjects who regularly use tobacco-containing products must refrain for 12 hours. After a 10-minute rest in a temperature-controlled room (68-70 F), the diameter of the right brachial artery blood flow are measured at baseline and in response to increased blood flow with a high-resolution (≥7.5 MHz) linear array vascular ultrasound transducer. Increased forearm blood flow, an endothelium-dependent vasodilator, is induced by inflating a pneumatic blood pressure tourniquet placed around the widest part of the forearm to a systolic blood pressure of 250 mm Hg., the release of which after 4.5 minutes leads to hyperemia. The change in brachial artery diameter in response to hyperemia relative to the baseline diameter defines FMD, which is described as a percentage change from baseline. A single-lead electrocardiogram is monitored throughout the study. Coronary and brachial artery flow-mediated vasodilation are correlated strongly (r=0.79, p<0.001).^240^ Endothelial function of the brachial artery also predicts future adverse cardiovascular events.^235,236^ In our laboratory, the coefficient of variation for FMD is 2.9% which compares favorably to that reported in the literature.^241-243^

*Advanced lipoprotein testing.* We intend to assess atherosclerotic risk and gain insight into observed changes in carotid IMT and endothelial function by assessing lipoprotein status using nuclear magnetic resonance (NMR) spectroscopy at baseline and during the three-year follow-up period of this grant. The NMR Lipoprofile (LipoScience, Inc. Raleigh, NC) provides a comprehensive assessment, including a large array of lipid sub-fractions that have been associated with atherosclerotic progression and cardiovascular events. Lipoproteins are also influenced by other factors associated with cessation such as weight gain and change in exercise rate.

Figure 3. Lipoprotein Subclasses Identified by NMR

Although total cholesterol levels are correlated with risk of cardiovascular disease, total cholesterol is not a good predictor of cardiovascular disease because cholesterol is a constituent of all major lipoproteins found in fasting plasma, including very-low density lipoproteins (VLDL), low-density lipoproteins (LDL), and high-density lipoproteins (HDL). Patients can have widely varying concentrations of these lipoproteins and have the same total cholesterol concentration.^244,245^ VLDL and LDL concentrations are associated positively with atherosclerotic vascular disease, whereas HDL concentrations are associated negatively with vascular disease. Lipoprotein metabolism is complex, however, and VLDL, LDL, and HDL each represent heterogeneous groups of particle subclasses that differ in size, lipid composition, and observed associations with cardiovascular disease.^244-250^

Advanced lipoprotein testing using NMR spectroscopy provides direct measurements of chylomicrons, VLDL, intermediate-density lipoproteins (IDL), LDL, and HDL and quantitative information about particle sizes (Figure 3).^244,245,247^ The NMR lipoprotein profile is necessary to interpret the physiological impact of metabolic changes and any changes in atherosclerosis progression and endothelial function that may be observed in this study. Recent data demonstrate that information about LDL particle concentration and LDL and HDL particle sizes improves cardiovascular disease risk prediction, even after controlling for standard lipid values.^244-250^ The limitations of standard enzymatic determinations of lipid levels are well known.^244,245^

The following methods will be used to determine NMR Lipoprofiles. After a 12 hour fast, approximately 2 cc of blood will be collected into a purple top EDTA specimen tube. This sample will be centrifuged immediately at 3000 rpm for 15 minutes. Plasma will be stored at -70°C and shipped to LipoScience, Inc.. The technique LipoScience, Inc. uses to acquire and process the NMR data has been described in detail^244,245,247^ and consists of three steps: 1) acquisition, in duplicate, of the 400 MHz proton NMR spectra from the plasma specimen at 47°C, 2) deconvolution of the lipid methyl group signal envelope appearing in the spectra at approximately 0.8 ppm, which yield the derived NMR signal amplitudes broadcasted by 16 modeled lipoprotein subclasses (Figure 4), and 3) conversion of the signal amplitudes into subclass concentrations using experimentally determined factors that relate the signal amplitudes of isolated subfraction standards to their chemically measured cholesterol and triglyceride concentrations. In general, large VLDL particles (V6, V5), IDL, small LDL (L1), and small HDL (H3, H2, H1) are considered atherogenic and associated with Metabolic Syndrome, as is an increased number of LDL particles.^244,245,247^

Figure 4. Relationship Between Plasma Lipid NMR Signal and Lipoprotein Subclasses

Carotid IMT, brachial artery enthothelial function testing and NMR lipoprofile testing will occur during Baseline of Project 1 (pre-quit). Because of the rapid changes in response expected for brachial artery endothelial function, it will be assessed again at one year post quit attempt. In contrast, carotid IMT will be assessed three years post quit when beneficial effects of cessation and detrimental effects of continued smoking are most likely to be seen. Finally, NMR Lipoprofile will be assessed at baseline, Year 1 and Year 3 post quit.

Other measures correlated with atherosclerotic risk will be assessed including blood pressure, weight, body mass index, heart and respiratory rate, creatinine, high-sensitivity C Reactive Protein, fasting plasma glucose and insulin, and hemoglobin A1C.

Physical Activity and Physical Fitness

*Physical activity*. This will be quantified using two methods: a physical activity questionnaire and pedometer measurements at baseline prequit, and at the ends of Years 1 & 3. The first is the International Physical Activity Questionnaire that covers all realms of physical activity (occupational, housework, etc.). This instrument has recently been shown to have acceptable reliability (Spearman’s rho = 0.80) and criterion-related validity (rho = 0.30), similar to other physical activity questionnaires.^251^ Questionnaires, however, tend not to be valid measures of moderate activity such as walking.^252^ Since walking is the most prevalent leisure-time activity in the U.S., is intrinsic to many daily routines, and is specifically mentioned in current nationwide physical activity recommendations^253^ it is particularly important to measure this specific activity. Pedometry has proven to be a very reliable and valid measure of steps walked when specific pedometer models are used.^254^

We propose to measure 7 days of walking annually with a Yamax Digiwalker SW-701. Subjects can be easily and quickly trained in the appropriate use of the pedometer and asked to fill out a simple log to record daily steps over the 7-day period.^255^ Through the use of both a simple questionnaire and a pedometer, we should be able to capture regular physical activity at targeted timepoints throughout the study.

*Physical fitness*. Each subject will have a symptom-limited treadmill test administered at baseline and at the 3-year follow-up. Peak oxygen consumption will be calculated from the final workload achieved on the test, and oxygen saturation (via pulse oximetry) will be measured at rest and continually during the test. The calculated oxygen consumption provides a measure of fitness, not physical activity, and is therefore a more objective measure than physical activity questionnaires and pedometry monitoring. This provides the opportunity to assess important changes in cardiorespiratory fitness associated with smoking cessation and physical activity.

While directly measuring oxygen consumption would provide a slightly more accurate assessment, the cost would be much greater. A modified Balke protocol will be used.^256^ Briefly, each subject will maintain a brisk walking pace and treadmill grade will be increased 2.5% every two minutes until volitional exhaustion. Oxygen saturation and 12-lead electrocardiogram (ECG) will be monitored continuously. ECG, heart rate, and blood pressure will be recorded at the end of each stage, maximum exercise, and every two minutes during recovery. The Borg scale rating of perceived exertion (RPE) ranging from 6 to 20 will be obtained near the end of each stage and maximum exercise. Differences will be compared between subject’s baseline prequit and three year follow-up tests for changes as they relate to smoking status. Changes in fitness based on these treadmill tests will be compared to other changes in the subject’s health status, including body weight, metabolic changes, and health outcomes.

Diet

Dietary factors will be assessed by a combination of a well documented food frequency questionnaire (FFQ), a qualitative screening food frequency questionnaire to track short-term changes, and objective biochemical indicators of diet (NMR Lipoproteins and, possibly, carotenoids).

The semi-quantitative FFQ to be used is that developed by Willett and colleagues,^257,258^ which has been continually updated and refined over time to account for secular changes. The questionnaire consists of approximately 130 food items with specified serving sizes, and for each item the participant is asked to report how often, on average over the past year, they have consumed that item. A multiple-choice response (nine categories) is provided. Nutrients are calculated using an extensive companion food composition database maintained at the Department of Nutrition, Harvard School of Public Health. The FFQ will be administered at baseline prequit and at annual in-person follow-up visits (12, 24, 36 months post quit attempt). The validity of this 130-item questionnaire has been demonstrated consistently on the bases of strong relations with multiple diet records (r’s > .60),^257,259^ and with biochemical markers of dietary intake (plasma beta-carotene, red cell folate, adipose linoleic acid, and others.^260-264^  Recently, a validation analysis using N-3 fatty acids from adipose aspirates and plasma carotenoids has demonstrated validity for the questionnaire in an African-American population in Detroit (MD Holmes, submitted manuscript).

For tracking short-term changes in diet we will use a brief screening questionnaire (PrimeScreen), also developed at Harvard School of Public Health. This questionnaire takes about 5 min to complete and has been validated with correlations with the full FFQ and via associations with selected biomarkers.^265^ For foods and food groups, the mean correlation coefficient (r) was 0.70 for reproducibility and 0.61 for comparability with the FFQ. For nutrients, the mean r was 0.74 for reproducibility and 0.60 for comparability with the FFQ. No substantial differences were evident by sex, race, body mass index, occupation or education. The PrimeScreen will be administered at follow-up phone contacts at 18 and 30 months post quit attempt.

###### Psychosocial

*Structured interview.* Mental health will be assessed in this study using the new computer-assisted National Comorbidity Survey-Replication Composite International Diagnostic Interview (the NCS-R-CIDI), which arose out of a joint project of the World Health Organization and the former US ADAMHA. The reliability and validity of the original paper-pencil CIDI was demonstrated in both large epidemiological studies as well as in smaller validity studies.^266-269^ The NCS-R-CIDI is now being used by three International Consortia as part of the World Mental Health 2000 Initiative,^270,271^ which seeks to characterize mental health in 27 countries (see Girolamo & Bassi^272^). The newer CIDI has been adapted for computer-assisted administration (versus the CIDI-Auto which is self-administered). It generates mental health diagnoses using DSM-IV criteria. The reliability and validity of the CIDI is supported by the extensive field testing of the instrument,^272^ the prior validation of the paper-pencil CIDI, its reliance on ICD-10 and DSM-IV criteria and earlier validation studies.^273^

The NCS-R-CIDI has notable strengths that make it appropriate for the present research. First, it permits assessment and diagnosis over three timeframes (lifetime, past year, and past month). In the present work we will administer the lifetime NCS-R-CIDI in Project 1: (Efficacy) and the past-year versions in the current project (Year 1, 2 & 3 follow-ups). In addition, it also assesses such relevant constructs as quality of life (daily functioning, social and family relationships), impact of mental health symptoms, and constructs relevant to physical illness (major diseases, use of clinicians). Also, it permits skip patterns so that individuals may be briefly screened for a disorder/condition, and it is suitable for administration by trained lay interviewers. NCS-trained staff will train lay interviews in all structured interview measures. Interviewers will be subject to repeated reliability assessments throughout the study.

We plan to administer the following NCS-R-CIDI components: mental health (Depression, Irritable Depression, Panic Disorder, Specific Phobia, Social Phobia, Agoraphobia, Generalized Anxiety Disorder, Suicidality, Alcohol Use, Tobacco Use, Neurasthenia, Obsessive-Compulsive Disorder, Worries and Unhappiness, and 30-Day Symptoms), physical health and health quality of life (Use of Services, 30-Day Functioning, Chronic Conditions). Importantly, we are deleting a number of CIDI scales in the interest of reducing burden (e.g., all childhood disorder scales, eating disorders, gambling, demographics).

These scales will provide critical information on diagnosis and dimensional information on mental health, physical health, their impact on quality of life, and health & mental health care utilization. These NCS-R-CIDI measures will be supplemented with other measures to achieve a greater sense of status in critical domains.

*Psychometric status of additional instruments.* The instruments used in this study (see Table 4) all have psychometric properties that support the inferences for which they are being used. For instance, the MPQ brief form (MPQ-BF) has excellent psychometric properties as a personality measure.^147^ The Wisconsin Dependence Measure (WISDM-68) the Wisconsin-Probability of Relapse and Smoking Measure (WI-PRISM), and the Fagerström Test of Nicotine Dependence (FTND) have been shown to predict dependence outcomes such as relapse and withdrawal severity.^138,156^ In fact, there is a substantial literature attesting to the positive psychometric properties of all of the measures that will be used: e.g., the PANAS,^274^ the Quality of Life Inventory,^275^ the Depression Proneness Inventory (DPI),^154,276^ and so on. The psychometric properties of other, chief measures were discussed earlier.

Genotyping

DNA sequencing will take place at the University of Utah Huntsman Cancer Institute. It has four ABI 3700’s and one ABI3730 which can run a total of 28,000 sequence ladders per week. SNP typing will be performed by the ABI SNPlex system. This system can detect 4500 SNPs in parallel in as little as 15 minutes. Extensive capabilities also exist in this center for bioinformatics that enable investigators to collect and analyze DNA sequence, haplotype and SNP data. DNA extraction is described in Project 1.

Compensation

Participants will be compensated for completion of vital study milestones. Specifically, we will compensate participants $100 for completing each of the three annual in-person study visits to take place at Months 12, 24, and 36 (total compensation $300). In addition, we will send participants birthday cards each year thanking them for their participation, and small gifts labeled with study identifiers and phone/email information (e.g., pens, refrigerator magnets, notebooks) (see “Steps to reduce attrition” above).

**Analytic Plan**

*Representing Constructs: Selection and Synthesis of Measures*. One virtue of this research is that many of the important constructs are assessed with multiple measures. The measures are not only psychometrically strong individually, but their conjoint use permits us to determine convergent validity. Moreover, some of these measures are collected in very different ways and this has the advantage of reducing method variance. While use of multiple measures confers advantages, it also requires decisions regarding which measures are to be used for which purposes.

The general approach we will adopt with dependent variables is as follows: when there are multiple measures of a type of dependent variable, we will examine the interrelations among the measures, determine the stability of findings across the class, and perhaps analyze a composite measure. For instance, with respect to *mental health* the NCS-R-CIDI yields categorical diagnoses and symptom counts. In addition it comprises measures of mental health quality of life, impact of mental health symptoms on daily functioning, and use of psychiatric services. We will explore how these different indicators are interrelated, and whether predictors bear similar relation to the different indicators. The latter will help us determine whether a finding is robust and stable. This strategy requires that we report findings across the different measures of common constructs. Finally, we may examine composite measures. For instance, in some analyses we plan to collapse across all major anxiety, affective, and substance use diagnoses (taking into account comorbidity). We plan to employ a similar strategy across the different major types of dependent variables that have multiple indicators: e.g., atherosclerosis, quality of life, nicotine dependence, alcohol use/abuse. For instance, alcohol variables will arise from time-line follow-back data, the Short Inventory of Problems, CIDI Diagnosis, and electronic diary data gathered in Project 1. Smoking relapse and calendar measures of smoking over each year will be produced using data generated by the time-line follow-back procedures used in follow-up interviews. This will produce measures of continuous abstinence, point-prevalence, and percent days smoking each year. Amount of smoking will be determined in each 7-day point prevalence period. Any of these could be used in analyses, but for modeling over time, percent days smoking over each year combined with heaviness measures should yield the most useful measures of tobacco exposure over time. Use of other tobacco or nicotine containing products will also be examined.

With respect to independent variables, we will use model-building procedures (described below) to systematically select best measures from a class, and also report prediction stability across measures.

*Data Acquisition and Preparation.* Data will be inspected on a continuous basis over the course of the study to ensure that data acquisition is satisfactory. Virtually all of the data will be captured via computer entry, scannable forms and palm-top computers. One further data source will be data obtained from medical tests. These typically do not generate computer data files and when they do not, they will be double-entered.

*Data Comparisons.* This study will permit us to address numerous important questions about how quitting smoking and relapse are related to health and psychosocial outcomes, and for whom particular outcomes are especially likely. We cannot outline all of the analyses permitted by this data set. However, we will present analytic plans for several important outcomes. These will reveal the strategies that we will use and the relations to be tested.

We will identify a critical question, use regression methods to identify significant predictors of ultimate endpoints, and then use structural models to explore more complex relations among significant predictors across time. We will assess genetic correlates of critical phenotypes (e.g., nicotine dependence, quitting success, mental health status, and atherosclerosis status and progression) in a parallel research track. We will eventually combine genetic and nongenetic variables in integrated prediction models to determine potential mediators of genetic relations (e.g., via personality) and whether candidate genes predict particular endophenotypes (e.g., smokers reporting escalating or prolonged withdrawal^124,125^). Construction of genetic models will follow the principles outlined below.

*Predicting Discrete Endpoints.* Many clinicians and others interested in public health will primarily be interested in best-fitting models that predict outcomes (e.g., psychiatric diagnostic outcomes such as depression, or diagnoses such as diabetes, quality of life) at some future point in time. To address the prediction of outcomes at discrete points in time, we will use logistic and multiple regression models (for categorical & continuous dependent variables, respectively) in which ultimate status at Year 2 or 3 is predicted by both prequit and postquit variables.

In testing predictive relations we will follow a general strategy consistent with the model building recommendations made by Hosmer & Lemeshow.^277^

- We will identify on an a priori, substantive basis, the major predictors to be tested, control variables, and critical interactions. Predictors will include both static and dynamic variables gathered both before and after the quit attempt.
- We will then examine distributions and use transformations where appropriate.
- Missingness will be addressed through direct Maximum Likelihood estimation^278^ assuming that ignorability assumptions are appropriate and standard error and test statistics are robust.^279^
- We will test univariate relations for all substantive variables so that their nonresidualized relations may be reported. Univariate variables will be modeled in various ways discussed in the section “Representing Constructs”) in order to determine the generalizability of obtained relations.
- We will use model-building procedures described below to identify best-fitting models using all promising candidate measures identified in univariate analyses.
- We will engage in *secondary analyses* to explore the relations of other variables with important targeted outcomes. These analyses will explore models that are less clinically or theoretically important than those tested in primary analyses, but ones that may provide the basis for future hypotheses and research. If reported, secondary analyses will clearly be labeled as exploratory.

All logistic regression models will be developed and tested using procedures and recommendations of Hosmer & Lemeshow.^277^ For instance, variable selection will start with a careful univariate analysis of each variable, and computation of smoothed scatterplots for continuous variables. After univariate analyses, all independent variables with P-values <0.25 will be candidates for multivariate analyses. Subsequent variable selection will occur in the context of forward selection with a test for backwards elimination. Subsequent to the identification of a multivariate logistic model, the performance of each variable will be evaluated via inspection of the Wald statistic, and the comparison of the multivariate and univariate errors and regression coefficients. If a new model is generated this will be compared with the larger former model via the likelihood ratio test. Eliminated variables will be re-entered to ensure that they have not become valid predictors through the exclusion of other variables. The performance of all continuous variables will be tested for linearity of the logit and any hypothesized (*a priori*) interactions tested. Model fit will be assessed via the Hosmer-Lemeshow test and via area under the ROC curve. Standard model diagnostics will be used (e.g., inspection of leverage values, variance of residuals for covariate patterns). Construction of multiple regression models will be similarly standardized and subjected to appropriate diagnostics.^280^

Model building will follow strategies frequently used in longitudinal research.^1^ Thus, *control variables* will be routinely entered (e.g., gender, age, site, ethnicity, Project 1 treatment, SES). Change will be modeled via entry of pretreatment (baseline) measures of the dependent variable and via change scores (which protect against interpretive biases of partialled scores.^281,282^ Next, relevant *individual differences* will be entered: e.g., nicotine dependence, personality, and genotypes, depending upon the analysis. Then other *modifiable risk factors* will be entered: i.e., measures of diet, alcohol use/abuse, weight/BMI/waist circumference, stressors/distress, exercise, blood pressure, serum lipids, and exposure to second hand smoke. Principal relations will be presented both in univariate and multivariate models. Two-way interactions to be tested will be based upon a priori hypotheses for each tested model.

*Principal Hypotheses*

Hypothesis 1: Smoking cessation (amount of abstinence over the follow-up period coded either dichotomously or continuously) will be associated with *reversal or arrest of atherosclerosis* as assessed via CIMT. Baseline CIMT scores will be used to determine change. *Individual differences and control variables*: Progression will be greater among males^283,284^ and among those high in trait (MPQ) Negative Emotionality.^2,4,8^ The following *modifiable risk factors* will predict greater atherosclerotic progression: weight gain, fat intake,^1^ BMI, second-hand smoke, sedentariness, and high levels of stress (e.g., ^5-7^). Exercise/activity, however, will predict greater regression. Two critical interactions with smoking will involve second-hand smoke and gender since research suggests different impacts of smoking depending upon these variables.^1,283^

Hypothesis 2. Smoking cessation/reduction will be associated with positive changes in *mental health functioning* (from baseline status). Change will be measured from either baseline (lifetime) history of diagnosis, or baseline symptom levels, depending upon the analysis. While cessation may initially increase depression/depressive symptoms, long-term cessation will be associated with improved mental health status as reflected by lower rates of psychiatric diagnoses and reduced symptoms (thus, some analyses will focus on Year 2 and 3 symptoms/diagnoses). *Individual differences and control variables*: post-cessation symptoms will be positively related to trait (MPQ) Negative Emotionality and female gender (especially if diagnoses are restricted to internalizing disorders) and severity of prequit nicotine dependence. *Modifiable risk factors*: mental health symptoms/diagnoses will be inversely related to post-quit social support and physical health, and positively related to post-quit stressor occurrence and frequency of postquit heavy drinking days (relevant to dependent variables that do not themselves comprise alcohol diagnoses). No interactions are predicted for this family of analyses.

Hypothesis 3. Smoking cessation/reduction will lead to improved *quality of life*. We expect continuing smokers to report diminishing QOL levels over the follow-up period (relative to baseline), and we expect exsmokers to report improving QOL. Change will be measured from baseline levels of tested measures (e.g., Quality of Life Questionnaire or other QOL indicator: e.g., 30 day functioning). *Individual differences and control variables*: Improvement in QOL will be inversely related to age.  *Modifiable risk factors:* QOL improvements will be inversely related to postquit heavy drinking days, postquit increase in BMI, post-quit measures of physical health symptoms (CIDI Chronic Conditions & 30 Day Symptoms) and positively related to postquit social support available in the social network. We make a further prediction that much of the improvement in QOL will be mediated by postquit psychiatric symptoms/disorders. We predict that the influence of smoking on QOL will be moderated by postquit weight gain/BMI as those gaining more weight will show a reduced relation between cessation and QOL improvements.^11^

Hypothesis 4. Smoking relapse late in the follow-up period (>1 yr postquit) will be related to presence of alcohol use in the person’s social network/dyad and to presence of smokers in the social network/dyad and alcohol use. Late relapse will be modeled both as 3-year point-prevalence or latency from 1-year post-quit. *Individual differences and control variables*: Importantly, baseline nicotine dependence will *not* predict late relapse, nor will female gender (although this may be because these factors may have already exerted their influence in the first year of follow-up which we will examine). *Modifiable risk factors:* We expect late relapse likelihood will be positively related to both number of drinking days and heavy drinking days (both postquit), to number of smokers in the social network and contact with such smokers (postquit), and to spousal smoking among partnered participants.

Hypothesis 5. Abstinence from smoking will be associated with reductions in the frequency of drinking-days in the post-quit period. Change will be determined from prequit frequency of drinking. *Individual differences and control variables*: Postquit drinking frequency will be positively related to male gender, and inversely related to MPQ Constraint and SES. *Modifiable risk factors:* Postquit drinking frequency will be inversely related to presence of drinking and smoking in the social network, and inversely related to exercise level. Finally, we expect an interaction between prequit diagnosis of alcohol abuse or alcohol problems (SIP) and smoking status. We believe these variables will reduce the impact of smoking cessation on drinking. Thus, smoking cessation will affect drinking frequency principally among those with fewer drinkers in their social network and who show no prequit drinking problems. These analyses will not reveal the temporal ordering of drinking and smoking influences, or their reciprocal effects over time. These issues will be addressed in structural models described below.

Secondary analyses. These will examine the predictors of the following postquit outcomes: (1) *physiological outcomes other than CIMT*: BART endothelial function, lipid measures, blood pressure, diabetes indicators, (2) *diet/nutrition/weight*: BMI, waist circumference, diet measures, (3) *physical fitness/activity*: pedometry, treadmill, IPAQ (4) *health-related quality of life*: NCS-R-CIDI physical symptoms & functioning, (5) *quality/nature of the social network*: size, smoking and alcohol use in network, supportiveness of network. It is important to note that the above models will be carefully refined with on-going consultation with our expert Co-Investigators in relevant fields.

*Structural Models.* We will use structural modeling to take advantage of the truly prospective, multiple measurement properties of the design. In particular we will use latent trajectory models (LTMs) to examine direct, indirect, and reciprocal relations among critical variables (including latent variables) over time. In these models intercepts and trajectories of repeated measures (e.g., smoking over time) will be modeled as random variables. In constructing trajectory models linear and quadratic trajectories will be modeled, as well as completely latent change functions since different inter-measure intervals may increase the value of shrinking or stretching time to capture trajectories.^285,286^ Analyses will be developed programmatically such that more complex structures will be tested only after characterization of simpler (nested) models. For instance, in modeling change in smoking or alcohol intake over time, we will first determine functional forms of these dynamic variables, then determine how they are influenced by key individual-difference exogenous variables (e.g., baseline nicotine dependence, history of psychiatric disorder), and then determine whether obtained relations are affected by inclusion of control variables (gender, site, ethnicity). Fit will be determined via conventional fit statistics.^287^

LTM methods allow for modeling of both mediational and moderating effects. In the former one can determine factors that mediate the effects of variables on the intercepts or trajectories of targeted variables (e.g., mental health outcomes). In the latter, one can determine *under what conditions* a relation exists between a predictor and a targeted construct. Moreover, we will use an autoregressive LTM to characterize the performance of two different variables measured repeatedly and their interdependencies over time.^286^ This will reveal, for example, whether time-specific values in drinking, relative to average drinking values, predict subsequent values in smoking (relative to average levels of smoking), and vice versa. There will be six repeated measures for follow-up phone variables and four measures for those gathered at in-person visits. (LTM will not be used for variables gathered only twice: e.g., Carotid IMT). Latent variables may be used for some central constructs (e.g., mental health status).

Figure 5 depicts an example of a multivariate unconditional latent trajectory model (LTM) for exploring alcohol use and smoking (modeled with only 4 repeated measures). Such models might also be used to explore relations between smoking and psychiatric symptoms, and between smoking and weight gain. Control variables such as those described in the “Predicting Discrete Endpoints” will be used as exogenous variables (MPQ Constraint, Gender, and Smoking in the Social Network). One exemplar set of predictions (Figure 5) would be that Gender will influence both initial drinking and smoking indices (Intercepts) with males showing higher levels. Low levels of trait Constraint would predict high initial smoking and drinking, and predict high levels of smoking over time. Post-quit smoking in the Social Network would also predict a reduced decline in smoking over time. Smoking and drinking might be inter-related in that higher smoking intercepts predict reduced declines in drinking over time.

Power

After accounting for relapse and attrition (initial N=900), we expect that data for at least 300 continuously abstinent and 450 smoking participants to be available for analysis. These estimates are based on the expectation that approximately 9% of the participants enrolled in Project 2 will either relapse (approximately 4% per year) or drop out of the study (approximately 5% per year) in years 2 and 3. The large number of primary and secondary hypotheses precludes presentation of power estimates for all possible comparisons. Two power analyses are presented below of central, discrete endpoint hypotheses.

*Long-term smoking status and atherosclerotic progression:* The best relevant estimates of CIMT effect sizes were reported by Howard et al.^1^ These authors analyzed CIMT change over a 3-year period in terms of smoking status (current, past, never) and ETS exposure. Howard et al.^1^ used mean common CIMT as the measure of progression (CIMT_mean_ ). The authors computed linear regression models that controlled for demographic, life-style, and other risk factors and found a significant difference between current and past smokers of 7.3 μm, equivalent to a 20% difference in progression.

## In the current study, we will use a more sensitive measure of carotid IMT (CIMT_max_ ) that averages segmental maximum carotid wall thicknesses from all measurable carotid artery segments (up to a maximum of six segments). Bots et al.^288^ recommended CIMT_max_ for intervention trials and also computed pooled estimates of the annual rate of CIMT change across multiple atherosclerosis trials. These pooled estimates were then used to compute sample size estimates for intervention trials. We use these same pooled estimates of CIMT_max_ change in power calculations for the current study. Other assumptions include: α=.05, a 20% difference in CIMT progression between abstinent participants (N = 300) and continuing smokers (N = 450), and use of the CIMT_max_ measure with an estimated change in CIMT for continuing smokers of 5.28 μm (SD= 5.0 μm).^288^ Based on these assumptions, we will have power of .81 to detect a 20% difference in progression between abstinent and smoking participants. This is a conservative power estimate as control variables should reduce error and our N is likely to exceed 750, and the projected effect size is smaller than that reported by Howard et al.^1^ Thus, we believe that the current study is adequately powered to detect predicted differences in CIMT progression over three years for the comparison of smokers versus continuously abstinent ex-smokers. Analyses of continuous measures of post-quit smoking also may yield greater power.

## Long-term smoking status and psychiatric morbidity. We predict that, compared to continuing smokers, abstinent ex-smokers will experience lower rates of occurrence of new psychiatric disorders during the 3-year follow-up. In one test of this hypothesis, we will use a composite measure of total incidence^289^ that combines mood, anxiety, and substance use disorders. Total incidence refers to disorder onsets (counting both new and recurrent cases) within a defined time period (e.g., 2 or 3 years). Power computation is difficult because to our knowledge, no population-based total incidence data for psychiatric disorders are available for smokers, ex-smokers, or never smokers. General population incidence rate data are available for certain psychiatric disorders (e.g., major depressive disorder, panic disorder) but these rates often exclude individuals with a past history of a given disorder (these are “initial incidence” rates) and the time frame is typically one year.

Although there are almost no data on total annual incidence of psychiatric disorders among quitting vs. continuing smokers (and hence, one reason for doing the proposed research), there are data that show incidence rates in the general population. An analysis of five Epidemiologic Catchment Area sites revealed a 1-year total incidence rate of about 12% for any diagnosis of affective, anxiety, or substance use disorders.^93^ However, we will be studying incidence over three years and the ECA data do not model the impact of recurrences. Perhaps the best data on 3-year incidence comes from De Graaf et al.^290^ These authors reported a 3-year incidence of 10% for initial diagnoses in those with no prior psychiatric diagnosis. We expect a higher incidence among nicotine dependent, continuing smokers given their higher rates of prevalence than nonsmokers (and hence higher rates than in the general population) and the majority of diagnoses tend to be recurrences.^93,291^ Regular smokers often have rates of psychiatric disorder that are 2-4 times the rates of nonsmokers (e.g., ^292,293^, and we expect their three-year total incidence rate to reflect that). We propose that continuing smokers will compile a 3-year incidence of 30% for affective, anxiety and alcohol diagnoses combined.^93^ We expect quitting to reduce 3-year incidence by about a third (a smaller effect size than was reported for a cessation-induced reduction of panic attacks.^84^ We believe such a decrease would be significant from a public health and clinical perspective.

Given the above assumptions, we predict that approximately 20% of abstinent participants and 30% of continuing smokers will develop a psychiatric disorder over the three-year post-quit period. Setting α=.05, the current study has power of > .80 to detect a difference of this magnitude. Table 5 provides power estimates for various rates in the abstinent ex-smokers and 30% for the continuing smokers. As shown in Table 5, our group sample sizes provide sufficient power to detect a difference in rates as small as 9% (i.e., 30% versus 21%) with power of .79.

It is important to observe that the above power estimate does not take into account the impact of a prior diagnosis history covariate which should be significantly related to post-quit incidence. Also, we expect to have even greater power to detect change in continuous symptom measures since the one relevant study^97^ suggests a moderate effect size.

**Limitations**

One limitation is that the smokers who volunteer, may not be representative of smokers in general. However, we will compare smokers in our study to smokers in general on the basis of SES, educational attainment, smoking rates, ethnicity, and so forth. Recent BRFSS data and Wisconsin Tobacco Survey data (2003) will provide opportunities to assess sample representativeness. Also, we will compare treatment adherers vs. dropouts in order to gauge attrition bias. In addition, those quitting in response to Project 1 treatments may not show the same outcomes as those making unaided quit attempts. As with any longitudinal study, this study does not permit absolute certainty with respect to causal inferences. Also, it is possible that the assessment activities involved in this research may produce reactivity: e.g., questions about smoking might elicit smoking urges and prompt relapse - - or medical assessments might make participants more health conscious and adopt healthier lifestyles.

Another important concern is that the assessment burden might cause many participants to refuse to enroll or to drop out. The research has many features designed to reduce or manage the burden: skip patterns, individualization of assessments, and compensation. We will regularly monitor participants’ reports of overload and strive to achieve maximal amounts of information while *placing greater emphasis on maintaining participants in the study*. Modern missingness techniques permit meaningful analysis despite incomplete data sets. Some outcomes (e.g., some diagnostic classes) will be infrequent and so cannot be analyzed as discrete outcomes with adequate power. However, many of these variables can be meaningfully analyzed as composites (e.g., collapsing across internalizing diagnoses). In addition, it is important to collect information on some infrequent outcomes since they provide a more comprehensive picture of the sample and its performance over time and can help establish the validity of some of the other measures.

# Connections with TTURC Theme and Companion Research Proposals

Each of the proposals in this TTURC application will advance our understanding of tobacco dependence treatments and treatment outcomes. The present study will track the health and psychosocial outcomes associated with quitting or continuing smoking over three years, targeting a broad array of important physical and mental domains. In this way, this project focuses on diverse ultimate outcomes associated with tobacco cessation. This study will complement other studies in this TTURC proposal that address other dimensions of treatment outcomes. For example, Project 1 will address short-term outcomes such as withdrawal symptoms and early abstinence. Project 4, *Healthcare Costs and Utilization of Smoking and Quitting*, also focuses on the longterm outcomes of a quit attempt, but in a different domain and context. Whereas the current project will track individual and dyad outcomes along health and psychosocial dimensions, Project 4 will track the systems-level health care cost and utilization outcomes associated with successful or unsuccessful quit attempts. Projects 2 and 4 also differ in terms of study population and research context. Whereas Project 2 involves intensive assessment of research volunteers seeking treatment, Project 4 involves unobtrusive assessment of primary care patients who accepted treatment offered as part of their normal healthcare. Conducting these projects jointly as part of the UW TTURC will advance our understanding of the immediate and delayed, and specific and global effects associated with tobacco dependence treatment in a way that no single project could accomplish-- by tackling core research questions on multiple levels of analysis, across distinct populations, and in diverse contexts.

**E. HUMAN SUBJECTS**

Risks to the Subjects

*Human Subjects Involvement and Characteristics:* While 1520 participants will be enrolled at the baseline of this study (Project 1: Efficacy), only 900 will participate in the proposed follow-up study (Project 2). All individuals who report 7-day point-prevalence abstinence at Week 52 of Project 1 (approximate n = 360) will be invited to participate in the proposed study. The remainder of Project 2 participants (approximate n = 540) will be randomly selected from all relapsed participants from Project 1. All participants will be 18 years of age or older. Inclusion criteria at baseline for Project 1 will be: (1) smoking at least 10 cigarettes/day (on average) for the previous 6 months, (2) a breath sample with a CO level > 9 ppm, (3) being motivated to quit, (4) able to read and write English, (5) agree to respond to EMA prompts throughout the day, (6) plan to remain in the treatment catchment area for at least 12 months after the initiation of treatment, (7) no medical contraindications for use of bupropion (e.g., no uncontrolled hypertension, history of bipolar illness, recent myocardial infarction, recent heavy alcohol use, seizure history, or use of MAO inhibitors or other contraindicated medications), (8) no imminent risk of suicide, (9) no other member of the household is participating, and (10) women are not pregnant and agree to prevent pregnancy during the course of study pharmacotherapy. There will be no restrictions on participation based on sex or race.

*Sources of Materials:* Participants in this program will provide data for the express purpose of research. Data will consist of answers to questionnaires and interviews assessing smoking history, demographics, nicotine dependence, personality, affect and affective vulnerability, psychopathology, stress, social networks, physical activity and food intake. Other assessments will include vital signs (blood pressure, heart and respiratory rate), body weight, waist circumference, height, breath samples to permit determination of carbon monoxide, blood samples for cotinine, DNA, lipid profiles, creatinine, simultaneous fasting blood glucose and insulin, hemoglobin A1C, c-reactive protein, and possibly blood carotenoids. Carbon monoxide and cotinine assays reflect smoking status. Data regarding lung and cardiovascular function will be collected using specific medical procedures, including ultrasound Carotid Intima-Media Thickness test (CIMT), Ultrasound Brachial Artery Reactivity Test (UBART) of endothelial functioning, and an exercise stress test.

*Potential Risks:* Although participants will have received cessation treatment as part of Project 1, they will not be receiving any treatment as a part of this proposed study, therefore, the risks associated with this research are judged to be minimal. The only significant risks are those posed by the exercise stress test. The most substantial risk associated with the exercise stress test is the risk of a coronary event. However, the risk of a coronary event in medically supervised exercise treadmill tests is less than 1 per 5000 - 10,000 tests, and events are usually minor in nature due to the test being supervised (for angina, dizziness, etc). Serious coronary or other events are rare due to the monitoring and the cessation of the test in the presence of ECG changes, any arrhythmias, abnormal symptoms, etc. Medical supervision will be available for all tests. One final potential risk includes the fact that participants will be informed about any urgent medical condition that they may have previously been unaware of (e.g., advanced atherosclerosis that might lead to stroke). The other medical assessments pose little or no risk. Brachial and carotid artery ultrasound studies are safe, noninvasive, and without discomfort. There are no known adverse physiologic effects related to diagnostic medical ultrasound. Subjects who elect not to participate in this research program, or are eliminated due to screening failure, will be given a list of alternative smoking cessation programs. Finally, we will not give subjects feedback regarding our assessments until the end of the entire follow-up study. However, if we discover evidence of a significant new disease (e.g., advanced carotid atherosclerosis suggesting a high risk of an acute stroke) we will inform the subject of the finding, and urge him or her to address it immediately. With the permission of the participant, we will send a letter to a health care provider.

Adequacy of Protection Against Risks

*Recruitment and Informed Consent:* As in our previous research, participants will be recruited at baseline via advertisements in newspapers, radio, television, billboards, and other media. In addition, free media (e.g., press conferences) will be utilized. Participants will be recruited in both Madison and Milwaukee. Advertisements and publicity will contain a phone number for interested individuals to call to contact study personnel. After calling this number, participants will undergo initial phone screening to rule out those with clear exclusions. The study will be briefly described, questions answered, and potentially qualifying individuals will be invited to attend an Orientation Session. At the Orientation Session, the general requirements for participation will be reviewed (e.g., session attendance, need for follow-up, participation in assessments, participation for at least 3 years). In addition, participants will be informed of the nature of the treatments involved. After answering additional questions about research participation and treatment, participants will be asked to read and sign a consent form as well as an appropriate HIPAA form. Subjects will also be given copies of the consent form and HIPAA form to take home. After signing a consent agreement, potential subjects will be scheduled for an individual screening session, where previously assessed inclusion/exclusion criteria will be confirmed and other inclusion/exclusion criteria will be assessed. Individuals will be encouraged to ask any further questions about the study protocol throughout the study.

*Protection Against Risk:* With respect to the treadmill stress electrocardiogram tests, participants will participate only if they do not have contraindications and are clinically stable. Participants will be screened prior to the test by trained physiologists for absolute and relative contraindications to stress testing, using physician reviewed protocols. Participants will have documented clearance by physicians for testing prior to the test, and informed consent will be obtained. Twelve-lead ECGs will be reviewed by a physician prior to the test to ensure that the participant does not have ECG or clinical contraindications to the test. Test termination criteria are also standard for stress testing, including the development of chest pain, more than 1 mm. of horizontal or downsloping ST segment depression in standard leads and other usual ECG criteria in addition to clinical criteria including abnormal hemodynamics, serious arrhythmias, abnormal symptoms, significant dyspnea, or abnormal oxygen saturation measurements. Subjects will be given a telephone number to contact the study physician if they experience serious adverse effects. Adverse events (AEs) and serious adverse events (SAEs) will be assessed at each study visit and study staff will verify that all adverse effects are being addressed either by the subject, his/her regular physician, or offer to have the study physician address the concern. In addition, suicidality will be assessed at every visit and follow-up contact. Individuals who report any suicidal ideation will be contacted by a licensed psychologist who will assess the level of risk and provide referrals as needed. Confidentiality of subject data and information will be accomplished by using subject numbers as unique identifiers, allowing us to keep subject data separate from identifying information. No publications or presentations resulting from this research program will contain any identifying information about individual participants.

Potential Benefits of the Proposed Research to the Subjects and Others

The potential benefits for smokers participating in this study include the chance to be informed of any urgent health risks of which they many not have been previously aware that become apparent as part of the study assessment procedures. The risks of this research are chiefly associated with the exercise stress test discussed above. In addition, this research has the potential to provide important information about quitting patterns and longterm health and psychosocial outcomes of quitting or not quitting smoking.

Importance of the Knowledge to be Gained

This is the first research study to provide longterm assessment of the physical health, mental health, lifestyle factors, and overall quality of life using state-of-the-art measures. The data from this study may allow researchers and clinicians to better understand the important health outcomes that result from quitting or not quitting smoking. The data will also allow researchers to understand the interactions among the different longterm outcomes. Given the minimal risks of the exercise stress test and the rigorous pre-treatment screening and the availability of both physicians and psychologists to address any adverse effects, we believe that the potential risks involved in participating in the study are outweighed by the benefits to both society and the individual.

# Participation of Women

Based on previous research conducted in Madison and Milwaukee (see Preliminary Studies), we project that approximately 56% of participants will be women. Because women are well represented in the two communities, there is no need for a specific recruitment strategy. The research plan does include analyses that will evaluate data by gender. Differential response to treatment based on gender is one of the central hypotheses of the research. See targeted enrollment table.

# Participation of Minorities

Previous work in Madison and Milwaukee (see Preliminary Studies) indicates that the proposed recruitment strategies will result in a diverse mix of participants. We anticipate approximately 17% African-American participants, with another 2% split between Native American/Alaska Native and Asian participants. Approximately 2% of participants will have Hispanic heritage. The research plan also includes some analyses that will evaluate data by minority status. Given the demographics of the communities where recruitment is occurring and the size of the study, it is anticipated that the study will recruit sufficient African American participants to allow these data to be separately evaluated. See targeted enrollment table.

# Participation of Children

Children under the age of 18 will not be included in the proposed clinical research because none of the medications being used in the proposed baseline study (Project 1: Efficacy) have been approved by the FDA for use by children under that age. Therefore, no children under the age of 18 will be in the eligible pool of participants from which participants for this proposed study will be drawn. Individuals between 18 and 21 will be eligible to participate. In the state of Wisconsin, persons of this age are allowed to consent to their own participation in clinical research.

Data and Safety Monitoring Plan

The following Data Safety and Monitoring Plan (DSMP) pertains to all research that is supported under the National Cancer Institute/National Institute on Drug Abuse/National Institute on Alcohol Abuse and Alcoholism Transdisciplinary Tobacco Use Research Center (TTURC) Award. This plan comprises not only the research conducted directly by the University of Wisconsin Center for Tobacco Research and Intervention (CTRI) researchers, but also research conducted by other investigators who are supported by TTURC funds. All investigators must agree to comply with the procedures outlined in this DSMP. This DSMP does not reduce any investigator’s obligation to comply with the requirements of the Institutional Review Board (IRB) at his/her home institution or the IRB of any collaborating organizations.

# *Monitoring the progress of trials and the safety of participants.* Each Project Principal Investigator is responsible for routine monitoring of the trial’s progress. This includes scheduled biweekly meetings with study staff and review of written documentation. Data that are reviewed at these meetings include the number and type of participants enrolled, the number and reasons for exclusions from enrollment, the number treated and the stage of treatment, summary of adverse events, individual review of serious adverse events and study participation and outcome data.

Additionally, each Project Principal Investigator is responsible for briefing the TTURC Principal Investigator on the trial’s progress on a regularly scheduled basis (typically biweekly). As data become available, the Data Manager, Project Principal Investigator, and TTURC Principal Investigator will review the data on a regularly scheduled basis (typically biweekly) to determine progress.

To facilitate participant safety, study participants must meet study inclusion criteria. Once enrolled in the trial, follow-up protocols will assess for the presence of adverse events. Should either excessive risk to study participants and/or lack of measurable benefit to study participants be determined, the study will be stopped and all participants notified in a manner appropriate to the nature of the risk and/or lack of benefit.

# *Plans for assuring compliance with requirements regarding the reporting of adverse events*. This DSMP requires that investigators notify the National Cancer Institute (NCI) and the University of Wisconsin IRB of the occurrence of any serious adverse event (SAE), or any adverse event (AE) which is severe, unexpected, and possibly related to study medication or protocol. Such notification must occur within five days of investigators becoming aware of the event.

If the study in question involves a pharmaceutical agent, and the serious adverse event might be related to drug use, both the Food and Drug Administration and the manufacturer will also be notified with five days of investigators becoming aware of the event. Examples of serious adverse events would be untoward medical or treatment occurrences that result in death, are life-threatening, require hospitalization or prolonging of existing hospitalization, create persistent or significant disability/incapacity, or involve congenital abnormality/birth defects. Unanticipated adverse events would include less serious problems that merit reporting because they are severe, unexpected, and possibly related to study participation. Any serious adverse event (SAE) will be queried and reported even if it appears that the serious adverse event is unrelated to treatment participation.

Not only will investigators report any serious adverse events (whether or not they are unanticipated), but the Principal Investigator of each project will also be responsible for the accurate documentation, investigation and follow-up of all study-related adverse events.

Adverse event assessment, recording, reporting and investigation will be accomplished through staff training, structured or standardized assessments of untoward occurrences/events, and regular monitoring by study investigators. The Project Principal Investigator and the TTURC Principal Investigators have ultimate responsibility for ensuring that serious adverse events are detected and reported in a timely manner. Additionally, the IRB will receive an annual report of all serious adverse events and adverse events meeting the criteria listed above.

*Plans for assuring that any action resulting in a temporary or permanent suspension of an NCI-funded clinical trial is reported to the NCI grant program director responsible for the grant.* The NCI grant program director will be notified within five days if the TTURC Principal Investigator deems it necessary to suspend a clinical trial. In the case of a temporary suspension, the TTURC Principal Investigator and Project Principal Investigator will develop a plan for continuation of the study and discuss this plan with the NCI grant program director in a reasonable time frame.

# *Plans for assuring data accuracy and confidentiality and protocol compliance*. The Data Manager and Project Principal Investigators will develop plans for assuring data accuracy and protocol compliance, which will be reviewed and approved by the TTURC Principal Investigator. Such plans will include data verification and protocol compliance checks. The Data Manager and Project Principal Investigator shall also be responsible for ensuring that the data for the project are securely stored, that storage is in compliance with University and federal regulations and that no unauthorized persons have access (electronic or physical) to any participant-identifiable data. In addition, all HIPAA regulations and guidelines will be followed.

# F. Vertebrate Animals – N/A

**G. Literature Cited**

1. Howard G, Wagenknecht LE, Burke GL, Diez-Roux A, Evans GW, McGovern P, Nieto FJ, Tell GS: Cigarette smoking and progression of atherosclerosis: The Atherosclerosis Risk in Communities (ARIC) Study. JAMA 1998, 279:119-124

2. Barefoot JC, Schroll M: Symptoms of depression, acute myocardial infarction, and total mortality in a community sample. Circulation 1996, 93:1976-1980

3. Matthews KA, Gump BB: Chronic work stress and marital dissolution increase risk of posttrial mortality in men from the Multiple Risk Factor Intervention Trial. Arch Intern Med 2002, 162:309-315

4. Smith TW, Ruiz JM: Psychosocial influences on the development and course of coronary heart disease: current status and implications for research and practice. J Consult Clin Psychol 2002, 70:548-568

5. Lacy CR, Contrada RJ, Robbins ML, Tannenbaum AK, Moreyra AE, Chelton S, Kostis JB: Coronary vasoconstriction induced by mental stress (simulated public speaking). Am J Cardiol 1995, 75:503-505

6. von Kanel R, Dimsdale JE, Ziegler MG, Mills PJ, Patterson TL, Lee SK, Grant I: Effect of acute psychological stress on the hypercoagulable state in subjects (spousal caregivers of patients with Alzheimer's disease) with coronary or cerebrovascular disease and/or systemic hypertension. Am J Cardiol 2001, 87:1405-1408

7. Whiteman MC, Deary IJ, Fowkes FG: Personality and social predictors of atherosclerotic progression: Edinburgh Artery Study. Psychosom Med 2000, 62:703-714

8. Goodman M, Quigley J, Moran G, Meilman H, Sherman M: Hostility predicts restenosis after percutaneous transluminal coronary angioplasty. Mayo Clin Proc 1996, 71:729-734

9. Parrott AC: Does cigarette smoking cause stress? American Psychologist 1999, 54:817-820

10. Solomon RL: An opponent-process theory of acquired motivation: The affective dynamics of addiction. Psychopathology: Experimental models. Edited by Seligman JDMaMEP. San Francisco, Witt Freeman, 1977, pp 66-103

11. Department of Health and Human Services: Women and smoking: A report of the Surgeon General. Rockville, MD, U.S. Department of Health and Human Services, 2001

12. Krall EA, Garvey AJ, Garcia RI: Smoking relapse after 2 years of abstinence: Findings from the VA Normative Aging Study. Nicotine & Tobacco Research 2002, 4:95-100

13. Gilpin EA, Pierce JP: Duration of *smoking* abstinence and success in quitting. Journal of the National Cancer Institute 1997, 89:572-577

14. Eisinger RA: Psychosocial predictors of smoking recidivism. J Health Soc Behav 1971, 12:355-362

15. Curtin JJ, Fairchild BA: Alcohol and cognitive control: implications for regulation of behavior during response conflict. J Abnorm Psychol 2003, 112:424-436

16. Robinson TE, Berridge KC: The neural basis of drug craving: an incentive-sensitization theory of addiction. Brain Res Brain Res Rev 1993, 18:247-291

17. Annual smoking-attributable mortality, years of potential life lost, and economic costs--United States, 1995-1999. MMWR 2002, 51:300-303

18. Fathi R, Marwick TH: Noninvasive tests of vascular function and structure: why and how to perform them. Am Heart J 2001, 141:694-703

19. Crouse JR, 3rd: Predictive value of carotid 2-dimensional ultrasound. Am J Cardiol 2001, 88:27E-30E

20. Chambless LE, Folsom AR, Clegg LX, Sharrett AR, Shahar E, Nieto FJ, Rosamond WD, Evans G: Carotid wall thickness is predictive of incident clinical stroke: the Atherosclerosis Risk in Communities (ARIC) study. American Journal of Epidemiology 2000, 151:478-487

21. Salonen JT, Salonen R: Ultrasound B-mode imaging in observational studies of atherosclerotic progression. Circulation 1993, 87:II56-1165

22. Burke GL, Evans GW, Riley WA, Sharrett AR, Howard G, Barnes RW, Rosamond W, Crow RS, Rautaharju PM, Heiss G: Arterial wall thickness is associated with prevalent cardiovascular disease in middle-aged adults. The Atherosclerosis Risk in Communities (ARIC) Study. Stroke 1995, 26:386-391

23. Chambless LE, Heiss G, Folsom AR, Rosamond W, Szklo M, Sharrett AR, Clegg LX: Association of coronary heart disease incidence with carotid arterial wall thickness and major risk factors: The Atherosclerosis Risk in Communities (ARIC) Study, 1987-1993. American Journal of Epidemiology 1997, 146:483-494

24. O'Leary DH, Polak JF, Kronmal RA, Manolio TA, Burke GL, Wolfson SK, Jr.: Carotid-artery intima and media thickness as a risk factor for myocardial infarction and stroke in older adults. Cardiovascular Health Study Collaborative Research Group. New England Journal of Medicine 1999, 340:14-22

25. Hodis HN, Mack WJ, LaBree L, Selzer RH, Liu CR, Liu CH, Azen SP: The role of carotid arterial intima-media thickness in predicting clinical coronary events. Annals of Internal Medicine 1998, 128:262-269

26. Howard G, Sharrett AR, Heiss G, Evans GW, Chambless LE, Riley WA, Burke GL: Carotid artery intimal-medial thickness distribution in general populations as evaluated by B-mode ultrasound. ARIC Investigators. Stroke 1993, 24:1297-1304

27. Greenland P, Abrams J, Aurigemma GP, Bond MG, Clark LT, Criqui MH, Crouse JR, 3rd, Friedman L, Fuster V, Herrington DM, Kuller LH, Ridker PM, Roberts WC, Stanford W, Stone N, Swan HJ, Taubert KA, Wexler L: Prevention Conference V: Beyond secondary prevention: identifying the high-risk patient for primary prevention: noninvasive tests of atherosclerotic burden: Writing Group III. Circulation 2000, 101:E16-22

28. Intima-media thickness and atherosclerosis: Predicting the risk? New York, Parthenon Publishing, 1997

29. Bonithon-Kopp C, Scarabin PY, Taquet A, Touboul PJ, Malmejac A, Guize L: Risk factors for early carotid atherosclerosis in middle-aged French women. Arterioscler Thromb 1991, 11:966-972

30. O'Leary DH, Polak JF, Kronmal RA, Kittner SJ, Bond MG, Wolfson SK, Jr., Bommer W, Price TR, Gardin JM, Savage PJ: Distribution and correlates of sonographically detected carotid artery disease in the Cardiovascular Health Study. The CHS Collaborative Research Group. Stroke 1992, 23:1752-1760

31. Bonithon-Kopp C, Touboul PJ, Berr C, Leroux C, Mainard F, Courbon D, Ducimetiere P: Relation of intima-media thickness to atherosclerotic plaques in carotid arteries. The Vascular Aging (EVA) Study. Arterioscler Thromb Vasc Biol 1996, 16:310-316

32. Howard G, Burke GL, Szklo M, Tell GS, Eckfeldt J, Evans G, Heiss G: Active and passive smoking are associated with increased carotid wall thickness. The Atherosclerosis Risk in Communities Study. Archives of Internal Medicine 1994, 154:1277-1282

33. Salonen R, Salonen JT: Progression of carotid atherosclerosis and its determinants: A population-based ultrasonography study. Atherosclerosis 1990, 81:33-40

34. Thomson CC, Rigotti NA: Hospital- and clinic-based smoking cessation interventions for smokers with cardiovascular disease. Prog Cardiovasc Dis 2003, 45:459-479

35. Yusuf S, Ounpuu S, Anand S: The global epidemic of atherosclerotic cardiovascular disease. Med Princ Pract 2002, 11 Suppl 2:3-8

36. Jabbour S, Reddy KS, Muna WF, Achutti A: Cardiovascular disease and the global tobacco epidemic: a wake-up call for cardiologists. Int J Cardiol 2002, 86:185-192

37. Bernhard D, Pfister G, Huck CW, Kind M, Salvenmoser W, Bonn GK, Wick G: Disruption of vascular endothelial homeostasis by tobacco smoke: impact on atherosclerosis. FASEB J 2003, 17:2302-2304

38. Kannel WB, Neaton JD, Wentworth D, Thomas HE, Stamler J, Hulley SB, Kjelsberg MO: Overall and coronary heart disease mortality rates in relation to major risk factors in 325,348 men screened for the MRFIT. Multiple Risk Factor Intervention Trial. Am Heart J 1986, 112:825-836

39. Braunwald E: Shattuck lecture--cardiovascular medicine at the turn of the millennium: triumphs, concerns, and opportunities. N Engl J Med 1997, 337:1360-1369

40. Freund KM, D'Agostino RB, Belanger AJ, Kannel WB, Stokes J, 3rd: Predictors of smoking cessation: the Framingham Study. Am J Epidemiol 1992, 135:957-964

41. Steenland K, Thun M, Lally C, Heath C, Jr.: Environmental tobacco smoke and coronary heart disease in the American Cancer Society CPS-II cohort. Circulation 1996, 94:622-628

42. Kawachi I, Colditz GA, Stampfer MJ, Willett WC, Manson JE, Rosner B, Hunter DJ, Hennekens CH, Speizer FE: Smoking cessation and decreased risks of total mortality, stroke, and coronary heart disease incidence among women: A prospective cohort study. Changes in cigarette-related disease risks and their implications for prevention and control. Edited by Burns DM, Garfinkel L, Samet JM. Bethesda, MD, National Cancer Institute Monograph, 1997, pp 531-565

43. Mowbray CT, Ribisl KM, Solomon M, Luke DA, Kewson TP: Characteristics of dual diagnosis patients admitted to an urban, public psychiatric hospital: an examination of individual, social, and community domains. Am J Drug Alcohol Abuse 1997, 23:309-326

44. Flegal KM, Troiano RP, Pamuk ER, Kuczmarski RJ, Campbell SM: The influence of smoking cessation on the prevalence of overweight in the United States. N Engl J Med 1995, 333:1165-1170

45. Howard G, Wagenknecht LE, Cai J, Cooper L, Kraut MA, Toole JF: Cigarette smoking and other risk factors for silent cerebral infarction in the general population. Stroke 1998, 29:913-917

46. Heiss G, Sharrett AR, Barnes R, Chambless LE, Szklo M, Alzola C: Carotid atherosclerosis measured by B-mode ultrasound in populations: associations with cardiovascular risk factors in the ARIC study. Am J Epidemiol 1991, 134:250-256

47. Belcaro G, Laurora G, Cesarone MR, De Sanctis MT, Incandela L, Barsotti A: Progression of subclinical atherosclerosis in 6 years. Ultrasound evaluation of the average, combined femoral and carotid bifurcation intima-media thickness. Vasa 1995, 24:227-232

48. Bovet P, Perret F, Cornuz J, Quilindo J, Paccaud F: Improved smoking cessation in smokers given ultrasound photographs of their own atherosclerotic plaques. Prev Med 2002, 34:215-220

49. Klesges RC, Winders SE, Meyers AW, Eck LH, Ward KD, Hultquist CM, Ray JW, Shadish WR: How much weight gain occurs following smoking cessation? A comparison of weight gain using both continuous and point prevalence abstinence. J Consult Clin Psychol 1997, 65:286-291

50. O'Hara P, Connett JE, Lee WW, Nides M, Murray R, Wise R: Early and late weight gain following smoking cessation in the Lung Health Study. Am J Epidemiol 1998, 148:821-830

51. Williamson DF, Madans J, Anda RF, Kleinman JC, Giovino GA, Byers T: Smoking cessation and severity of weight gain in a national cohort. N Engl J Med 1991, 324:739-745

52. Froom P, Melamed S, Benbassat J: Smoking cessation and weight gain. J Fam Pract 1998, 46:460-464

53. Burnette MM, Meilahn E, Wing RR, Kuller LH: Smoking cessation, weight gain, and changes in cardiovascular risk factors during menopause: the Healthy Women Study. Am J Public Health 1998, 88:93-96

54. Becona E, Vazquez FL: Smoking cessation and weight gain in smokers participating in a behavioral treatment at 3-year follow-up. Psychol Rep 1998, 82:999-1005

55. Caan B, Coates A, Schaefer C, Finkler L, Sternfeld B, Corbett K: Women gain weight 1 year after smoking cessation while dietary intake temporarily increases. J Am Diet Assoc 1996, 96:1150-1155

56. Emont SL, Cummings KM: Weight gain following smoking cessation: a possible role for nicotine replacement in weight management. Addict Behav 1987, 12:151-155

57. Frederick SL, Hall, S.M., Fumfleet G.L., Munoz, R.F.: Sex differences in the relation of mood to weight gain after quitting smoking. Exp Clin Psychopharmacol 1996, 4:178-185

58. Klesges RC, Ward KD, Ray JW, Cutter G, Jacobs DR, Jr., Wagenknecht LE: The prospective relationships between smoking and weight in a young, biracial cohort: the Coronary Artery Risk Development in Young Adults Study. J Consult Clin Psychol 1998, 66:987-993

59. Stamford BA, Matter S, Fell RD, Papanek P: Effects of smoking cessation on weight gain, metabolic rate, caloric consumption, and blood lipids. Am J Clin Nutr 1986, 43:486-494

60. Expert Panel on Detection E, and Treatment of High Blood Cholesterol in Adults,: National cholesterol education program: Adult treatment panel III report. Publication no. 01-3095. Bethesda, MD, National Heart, Lung, and Blood Institute, 2001

61. Barrett-Connor E, Khaw KT: Cigarette smoking and increased central adiposity. Ann Intern Med 1989, 111:783-787

62. Revicki D, Sobal J, DeForge B: Smoking status and the practice of other unhealthy behaviors. Fam Med 1991, 23:361-364

63. French SA, Hennrikus DJ, Jeffery RW: Smoking status, dietary intake, and physical activity in a sample of working adults. Health Psychol 1996, 15:448-454

64. McTiernan A, Stanford JL, Daling JR, Voigt LF: Prevalence and correlates of recreational physical activity in women aged 50-64 years. Menopause 1998, 5:95-101

65. Britton JA, Gammon MD, Kelsey JL, Brogan DJ, Coates RJ, Schoenberg JB, Potischman N, Swanson CA, Stanford JL, Brinton LA: Characteristics associated with recent recreational exercise among women 20 to 44 years of age. Women Health 2000, 31:81-96

66. Perkins KA: Weight gain following smoking cessation. J Consult Clin Psychol 1993, 61:768-777

67. Sternfeld B, Ainsworth BE, Quesenberry CP: Physical activity patterns in a diverse population of women. Prev Med 1999, 28:313-323

68. Perkins KA, Rohay J, Meilahn EN, Wing RR, Matthews KA, Kuller LH: Diet, alcohol, and physical activity as a function of smoking status in middle-aged women. Health Psychol 1993, 12:410-415

69. Kawachi I, Troisi RJ, Rotnitzky AG, Coakley EH, Colditz GA: Can physical activity minimize weight gain in women after smoking cessation? Am J Public Health 1996, 86:999-1004

70. Marcus BH, Albrecht AE, King TK, Parisi AF, Pinto BM, Roberts M, Niaura RS, Abrams DB: The efficacy of exercise as an aid for smoking cessation in women: a randomized controlled trial. Arch Intern Med 1999, 159:1229-1234

71. McHenry PL, Faris JV, Jordan JW, Morris SN: Comparative study of cardiovascular function and ventricular premature complexes in smokers and nonsmokers during maximal treadmill exercise. Am J Cardiol 1977, 39:493-498

72. Leon AS, Jacobs DR, Jr., DeBacker G, Taylor HL: Relationship of physical characteristics and life habits to treadmill exercise capacity. American Journal of Epidemiology 1981, 113:653-660

73. Lochan M, Rasmussen, K: The Tromso study: Physical fitness, self reported phusical activity, and their relationship to other coronary risk factors. Journal of Epidemiology and Community Health 1992, 46:103-107

74. Conway TL, Cronan TA: Smoking, exercise, and physical fitness. Preventive Medicine 1992, 21:723-734

75. Sandvik L, Erikssen G, Thaulow E: Long term effects of smoking on physical fitness and lung function: a longitudinal study of 1393 middle aged Norwegian men for seven years. British Medcial Journal 1995, 311:715-718

76. Sidney S, Haskell WL, Crow R, Sternfeld B, Oberman A, Armstrong MA, Cutter GR, Jacobs DR, Savage PJ, Van Horn L: Symptom-limited graded treadmill exercise testing in young adults in the CARDIA study. Med Sci Sports Exerc 1992, 24:177-183

77. Albrecht AE, Marcus BH, Roberts M, Forman DE, Parisi AF: Effect of smoking cessation on exercise performance in female smokers participating in exercise training. Am J Cardiol 1998, 82:950-995

78. Lauer MS, Pashkow FJ, Larson MG, Levy D: Association of cigarette smoking with chronotropic incompetence and prognosis in the Framingham Heart Study. Circulation 1997, 96:897-903

79. Gordon DJ, Leon AS, Ekelund LG, Sopko G, Probstfield JL, Rubenstein C, Sheffield LT: Smoking, physical activity, and other predictors of endurance and heart rate response to exercise in asymptomatic hypercholesterolemic men. The Lipid Research Clinics Coronary Primary Prevention Trial. American Journal of Epidemiology 1987, 125:587-600

80. Srivastava R, Blackstone EH, Lauer MS: Association of smoking with abnormal exercise heart rate responses and long-term prognosis in a healthy, population-based cohort. Am J Med 2000, 109:20-26

81. Sidney S, Sternfeld B, Gidding SS, Jacobs DR, Jr., Bild DE, Oberman A, Haskell WL, Crow RS, Gardin JM: Cigarette smoking and submaximal exercise test duration in a biracial population of young adults: The CARDIA study. Med Sci Sports Exerc 1993, 25:911-916

82. Perkins KA: Effects of tobacco smoking on caloric intake. Br J Addict 1992, 87:193-205

83. Hall SM, McGee R, Tunstall C, Duffy J, Benowitz N: Changes in food intake and activity after quitting smoking. J Consult Clin Psychol 1989, 57:81-86

84. Breslau N, Klein DF: Smoking and panic attacks: an epidemiologic investigation. Arch Gen Psychiatry 1999, 56:1141-1147

85. Johnson JG, Cohen P, Pine DS, Klein DF, Kasen S, Brook JS: Association between cigarette smoking and anxiety disorders during adolescence and early adulthood. JAMA 2000, 284:2348-2351

86. Jorm AF: Association between smoking and mental disorders: results from an Australian National Prevalence Survey. Aust N Z J Public Health 1999, 23:245-248

87. Lasser K, Boyd JW, Woolhandler S, Himmelstein DU, McCormick D, Bor DH: Smoking and mental illness: A population-based prevalence study. JAMA 2000, 284:2606-2610

88. Borrelli B, Abrams D, Niaura R, Keuthen N, Goldstein M, DePue J, Murphy C: Development of major depressive disorder during smoking-cessation treatment. Journal of Clinical Psychiatry 1996, 57:534-538

89. Covey LS, Glassman AH, Stetner F: Major depression following smoking cessation. Am J Psychiatry 1997, 154:263-265

90. Killen JD, Fortmann SP, Schatzberg A, Hayward C, Varady A: Onset of major depression during treatment for nicotine dependence. Addictive Behaviors 2003, 28:461-470

91. Tsoh JY, Humfleet GL, Munoz RF, Reus VI, Hartz DT, Hall SM: Development of major depression after treatment for smoking cessation. The American Journal of Psychiatry 2000, 157:368-374

92. Eaton WW, Anthony JC, Gallo J, Cai G, Tien A, Romanoski A, Lyketsos C, Chen LS: Natural history of Diagnostic Interview Schedule/DSM-IV major depression. The Baltimore Epidemiologic Catchment Area follow-up. Arch Gen Psychiatry 1997, 54:993-999

93. Regier DA, Narrow WE, Rae DS, Manderscheid RW, Locke BZ, Goodwin FK: The de facto US mental and addictive disorders service system. Epidemiologic catchment area prospective 1-year prevalence rates of disorders and services. Arch Gen Psychiatry 1993, 50:85-94

94. Mulder I, Tijhuis M, Smit HA, Kromhout D: Smoking cessation and quality of life: The effect of amount of smoking and time since quitting. Preventive Medicine 2001, 33:653-660

95. Tillmann M, Silcock J: A comparison of smokers' and ex-smokers' health-related quality of life. J Public Health Med 1997, 19:268-273

96. Stewart AL, King, A. C., Killen, J. D., & Ritter, P.L.: Does smoking cessation improve health-related quality of life? Annals of Behavioral Medicine 1995, 17:331-338

97. Mino Y, Shigemi J, Otsu T, Tsuda T, Babazono A: Does smoking cessation improve mental health? Psychiatry Clin Neurosci 2000, 54:169-172

98. Breslau N: Psychiatric comorbidity of smoking and nicotine dependence. Behavior Genetics 1995, 25:95-101

99. Batel P, Pessione F, Maitre C, Rueff B: Relationship between alcohol and tobacco dependencies among alcoholics who smoke. Addiction 1995, 90:977-980

100. Burling TA, Ziff D: Tobacco smoking: a comparison between alcohol and drug abuse inpatients. Addictive Behaviors 1988, 13:185-190

101. McClure JB, Wetter DW, de Moor C, Cinciripini PM, Gritz ER: The relation between alcohol consumption and smoking abstinence: Results from the Working Well Trial. Addictive Behaviors 2002, 27:367-379

102. Miller NS, Gold MS: Comorbid cigarette and alcohol addiction: epidemiology and treatment. J Addict Dis 1998, 17:55-66

103. Breslau N, Peterson E, Schultz L, Andreski P, Chilcoat H: Are smokers with alcohol disorders less likely to quit? American Journal of Public Health 1996, 86:985-990

104. John U, Hanke M: Tobacco-smoking prevalence among physicians and nurses in countries with different tobacco-control activities. Eur J Cancer Prev 2003, 12:235-237

105. Carmelli D, Swan GE, Robinette D: The relationship between quitting smoking and changes in drinking in World War II veteran twins. J Subst Abuse 1993, 5:103-116

106. Murray RP, Istvan JA, Voelker HT, Rigdon MA, Wallace MD: Level of involvement with alcohol and success at smoking cessation in the lung health study. J Stud Alcohol 1995, 56:74-82

107. Keenan RM, Hatsukami DK, Pickens RW, Gust SW, Strelow LJ: The relationship between chronic ethanol exposure and cigarette smoking in the laboratory and the natural environment. Psychopharmacology (Berl) 1990, 100:77-83

108. Maletsky BM, & Klotter, J.: Smoking and alcoholism. American Journal of Psychiatry 1974, 131:445-447

109. Murray RP, Cribbie RA, Istvan JA, Barnes GE: Longitudinal analysis of the relationship between changes in smoking and changes in drinking in a community sample: The Winnipeg Health and Drinking Survey. Health Psychology 2002, 21:237-243

110. Nothwehr F, Lando HA, Bobo JK: Alcohol and tobacco use in the Minnesota Heart Health Program. Addictive Behaviors 1995, 20:463-470

111. Slutske WS, Heath AC, Madden PA, Bucholz KK, Statham DJ, Martin NG: Personality and the genetic risk for alcohol dependence. J Abnorm Psychol 2002, 111:124-133

112. Bierut LJ, Rice JP, Goate A, Hinrichs AL, Saccone NL, Foroud T, Edenberg HJ, Cloninger CR, Begleiter H, Conneally PM, Crowe RR, Hesselbrock V, Li TK, Nurnberger JI, Jr., Porjesz B, Schuckit MA, Reich T: A genomic scan for habitual smoking in families of alcoholics: common and specific genetic factors in substance dependence. Am J Med Genet 2004, 124A:19-27

113. Franks MM, Pienta AM, Wray LA: It takes two: Marriage and smoking cessation in the middle years. Journal of Aging and Health 2002, 14:336-354

114. Burman B, Margolin G: Analysis of the association between marital relationships and health problems: an interactional perspective. Psychol Bull 1992, 112:39-63

115. Coyne JC, Smith, D.A. F.: Couples coping with a myocardialo infarction: contextual perspective on self-efficacy. Journal of Family Psychology 1994, 8:1-13

116. Ross CE, Mirowsky J, Goldsteen K: The impact of the family on health: The decade in review. Journal of Marriage and the Family 1990, 52:1059-1078

117. Chassin L, Presson CC, Sherman SJ, Kim K: Long-term psychological sequelae of smoking cessation and relapse. Health Psychol 2002, 21:438-443

118. Cohen S, Lichtenstein E: Perceived stress, quitting smoking, and smoking relapse. Health Psychol 1990, 9:466-478

119. Chassin L, Presson CC, Pitts SC, Sherman SJ: The natural history of cigarette smoking from adolescence to adulthood in a midwestern community sample: multiple trajectories and their psychosocial correlates. Health Psychol 2000, 19:223-231

120. Baker TB, Piper ME, McCarthy DE, Majeskie MR, Fiore MC: Addiction motivation reformulated: An affective processing model of negative reinforcement. Psychol Rev (in press)

121. Wilson D, Parsons J, Wakefield M: The health-related quality-of-life of never smokers, ex-smokers, and light, moderate, and heavy smokers. Prev Med 1999, 29:139-144

122. Woolf SH, Rothemich SF, Johnson RE, Marsland DW: Is cigarette smoking associated with impaired physical and mental functional status? An office-based survey of primary care patients. Am J Prev Med 1999, 17:134-137

123. Kenford SL, Smith SS, Wetter DW, Jorenby DE, Fiore MC, Baker TB: Predicting relapse back to smoking: Contrasting affective and physical models of dependence. Journal of Consulting and Clinical Psychology 2002, 70:216-227

124. Piasecki TM, Jorenby DE, Smith SS, Fiore MC, Baker TB: Smoking withdrawal dynamics: II. Improved tests of withdrawal-relapse relations. Journal of Abnormal Psychology 2003, 112:14-27

125. Piasecki TM, Jorenby DE, Smith SS, Fiore MC, Baker TB: Smoking withdrawal dynamics: I. Abstinence distress in lapsers and abstainers. Journal of Abnormal Psychology 2003, 112:3-13

126. Shiffman S, Gnys M, Richards TJ, Paty JA, Hickcox M, Kassel JD: Temptations to smoke after quitting: A comparison of lapsers and maintainers. Health Psychology 1996, 15:455-461

127. Piper ME, Curtin JJ: The effects of withdrawal on voluntary affect regulation. Talk presented at the 10th Annual Meeting of the Society for Research on Nicotine and Tobacco, Scottsdale, AZ, 2004

128. Mermelstein R, Cohen S, Lichtenstein E, Baer JS, Kamarck T: Social support and smoking cessation and maintenance. Journal of Consulting & Clinical Psychology 1986, 54:447-453

129. Killen JD, Fortmann SP, Kraemer HC, Varady AN, Davis L, Newman B: Interactive effects of depression symptoms, nicotine dependence, and weight change on late smoking relapse. Journal of Consulting and Clinical Psychology 1996, 64:1060-1067

130. Swan GE, Denk CE, Parker SD, Carmelli D, Furze CT, Rosenman RH: Risk factors for late relapse in male and female ex-smokers. Addict Behav 1988, 13:253-266

131. Ockene JK, Emmons KM, Mermelstein RJ, Perkins KA, Bonollo DS, Voorhees CC, Hollis JF: Relapse and maintenance issues for smoking cessation. Health Psychology 2001, 19:17-31

132. Hall SM, Delucchi KL, Velicer WF, Kahler CW, Ranger-Moore J, Hedeker D, Tsoh JY, Niaura R: Statistical analysis of randomized trials in tobacco treatment: longitudinal designs with dichotomous outcome. Nicotine Tob Res 2001, 3:193-202

133. Piasecki TM, Fiore MC, McCarthy DE, Baker TB: Have we lost our way? The need for dynamic formulations of smoking relapse proneness. Addiction 2002, 97:1093-1108

134. Luedemann J, Schminke U, Berger K, Piek M, Willich SN, Doring A, John U, Kessler C: Association between behavior-dependent cardiovascular risk factors and asymptomatic carotid atherosclerosis in a general population. Stroke 2002, 33:2929-2935

135. Schmitz N, Kruse J, Kugler J: Disabilities, quality of life, and mental disorders associated with smoking and nicotine dependence. Am J Psychiatry 2003, 160:1670-1676

136. Batra V, Patkar AA, Berrettini WH, Weinstein SP, Leone FT: The genetic determinants of smoking. Chest 2003, 123:1730-1739

137. Rossing MA: Genetic influences on smoking: candidate genes. Environ Health Perspect 1998, 106:231-238

138. Piper ME, Piasecki TM, Federman EB, Bolt DM, Smith SS, Fiore MC, Baker TB: A multiple motives approach to tobacco dependence: The Wisconsin Inventory of Smoking Dependence Motives (WISDM-68). Journal of Consulting & Clinical Psychology (in press)

139. Arinami T, Ishiguro H, Onaivi ES: Polymorphisms in genes involved in neurotransmission in relation to smoking. Eur J Pharmacol 2000, 410:215-226

140. Lerman C, Caporaso NE, Audrain J, Main D, Bowman ED, Lockshin B, Boyd NR, Shields PG: Evidence suggesting the role of specific genetic factors in cigarette smoking. Health Psychol 1999, 18:14-20

141. Dick DM, Foroud T: Candidate genes for alcohol dependence: a review of genetic evidence from human studies. Alcohol Clin Exp Res 2003, 27:868-879

142. Rauramaa R, Vaisanen SB, Luong LA, Schmidt-Trucksass A, Penttila IM, Bouchard C, Toyry J, Humphries SE: Stromelysin-1 and interleukin-6 gene promoter polymorphisms are determinants of asymptomatic carotid artery atherosclerosis. Arterioscler Thromb Vasc Biol 2000, 20:2657-2662

143. Karvonen MK, Valkonen VP, Lakka TA, Salonen R, Koulu M, Pesonen U, Tuomainen TP, Kauhanen J, Nyyssonen K, Lakka HM, Uusitupa MI, Salonen JT: Leucine7 to proline7 polymorphism in the preproneuropeptide Y is associated with the progression of carotid atherosclerosis, blood pressure and serum lipids in Finnish men. Atherosclerosis 2001, 159:145-151

144. de Waart FG, Kok FJ, Smilde TJ, Hijmans A, Wollersheim H, Stalenhoef AF: Effect of glutathione S-transferase M1 genotype on progression of atherosclerosis in lifelong male smokers. Atherosclerosis 2001, 158:227-231

145. Risley P, Jerrard-Dunne P, Sitzer M, Buehler A, von Kegler S, Markus HS: Promoter polymorphism in the endotoxin receptor (CD14) is associated with increased carotid atherosclerosis only in smokers: the Carotid Atherosclerosis Progression Study (CAPS). Stroke 2003, 34:600-604

146. Krueger RF, Hicks BM, Patrick CJ, Carlson SR, Iacono WG, McGue M: Etiologic connections among substance dependence, antisocial behavior, and personality: modeling the externalizing spectrum. J Abnorm Psychol 2002, 111:411-424

147. Patrick CJ, Curtin JJ, Tellegen A: Development and validation of a brief form of the Multidimensional Personality Questionnaire. Psychol Assess 2002, 14:150-163

148. Ory MG, Jordan PJ, Bazzarre T: The Behavior Change Consortium: setting the stage for a new century of health behavior-change research. Health Educ Res 2002, 17:500-511

149. Sobell LC, Sobell MB, Agrawal S: Self-change and dual recoveries among individuals with alcohol and tobacco problems: current knowledge and future directions. Alcohol Clin Exp Res 2002, 26:1936-1938

150. Strecher V, Wang C, Derry H, Wildenhaus K, Johnson C: Tailored interventions for multiple risk behaviors. Health Educ Res 2002, 17:619-626

151. Jorenby DE, Leischow SJ, Nides MA, Rennard SI, Johnston JA, Hughes AR, Smith SS, Muramoto ML, Daughton DM, Doan K, Fiore MC, Baker TB: A controlled trial of sustained-release bupropion, a nicotine patch, or both for smoking cessation. New England Journal of Medicine 1999, 340:685-691

152. Piper ME, Federman EB, Smith SS, Fiore MC, Baker TB: Efficacy of bupropion SR alone and combined with 4-mg nicotine gum. Talk to be presented at the 10th Annual Meeting of the Society for Research on Nicotine and Tobacco, Scottsdale, AZ, 2004

153. Fiore MC, McCarthy DE, Jackson TC, Zehner ME, Jorenby DE, Mielke MM, Smith S, Guiliani TA, Baker TB: Integrating smoking cessation treatment into primary care: An effectiveness study. Preventive Medicine (in press)

154. Smith SS, Jorenby DE, Fiore MC, Anderson JE, Mielke MM, Beach KE, Piasecki TM, Baker TB: Strike while the iron is hot: Can stepped-care treatments resurrect relapsing smokers? Journal of Consulting & Clinical Psychology 2001, 69:429-439

155. McCarthy DE, Fiore MC, Piasecki TM, Baker TB: Life before and after quitting smoking. Journal of Abnormal Psychology (under review)

156. Piper ME, Ma GX, Bolt DM, McCarthy DE, Baker TB: The Wisconsin Predicting Relapse in Smoking Measure (WI-PRISM) - A new measure of relapse proneness. Poster presented at the 10th Annual Meeting of the Society for Research on Nicotine and Tobacco Scottsdale, Az (in preparation)

157. Welsch SK, Smith SS, Wetter DW, Jorenby DE, Fiore MC, Baker TB: Development and validation of the Wisconsin Smoking Withdrawal Scale. Experimental and Clinical Psychopharmacology 1999, 7:354-361

158. Piasecki TM, Jorenby DE, Smith SS, Fiore MC, Baker TB: Smoking Withdrawal Dynamics: III. Correlates of Withdrawal Heterogeneity. Exp Clin Psychopharmacol 2003, 11:276-285

159. Smith SS, Jorenby DE, Leischow SJ, Nides MA, Rennard SI, Johnston JA, Jamerson B, Fiore MC, Baker TB: Targeting smokers at increased risk for relapse: Treating women and those with a history of depression. Nicotine & Tobacco Research 2003, 5:99-109

160. Colbert LH, Hootman JM, Macera CA: Physical activity-related injuries in walkers and runners in the aerobics center longitudinal study. Clin J Sport Med 2000, 10:259-263

161. Colbert LH, Lacey JV, Jr., Schairer C, Albert P, Schatzkin A, Albanes D: Physical activity and risk of endometrial cancer in a prospective cohort study (United States). Cancer Causes Control 2003, 14:559-567

162. Colbert LH, Hartman TJ, Malila N, Limburg PJ, Pietinen P, Virtamo J, Taylor PR, Albanes D: Physical activity in relation to cancer of the colon and rectum in a cohort of male smokers. Cancer Epidemiol Biomarkers Prev 2001, 10:265-268

163. Colbert LH, Hartman TJ, Tangrea JA, Pietinen P, Virtamo J, Taylor PR, Albanes D: Physical activity and lung cancer risk in male smokers. Int J Cancer 2002, 98:770-773

164. Limburg PJ, Stolzenberg-Solomon RZ, Colbert LH, Perez-Perez GI, Blaser MJ, Taylor PR, Virtamo J, Albanes D: Helicobacter pylori seropositivity and colorectal cancer risk: A prospective study of male smokers. Cancer Epidemiology Biomarkers & Prevention 2002, 11:1095-1099

165. Fleming MF: Brief interventions and the treatment of alcohol use disorders: current evidence. Recent Dev Alcohol 2003, 16:375-390

166. Fleming MF, Graham AW: Screening and brief interventions for alcohol use disorders in managed care settings. Recent Dev Alcohol 2001, 15:393-416

167. Fleming MF, Manwell LB, Barry KL, Johnson K: At-risk drinking in an HMO primary care sample: prevalence and health policy implications. Am J Public Health 1998, 88:90-93

168. Fleming MF, Mundt MP, French MT, Manwell LB, Stauffacher EA, Barry KL: Brief physician advice for problem drinkers: long-term efficacy and benefit-cost analysis. Alcohol Clin Exp Res 2002, 26:36-43

169. Murray M, Fleming M: Prevention and treatment of alcohol-related problems: an international medical education model. Acad Med 1996, 71:1204-1210

170. Brown RL, Fleming MF: Training the trainers: substance abuse screening and intervention. Int J Psychiatry Med 1998, 28:137-146

171. Fleming MF, Barry KL, Manwell LB, Johnson K, London R: Brief physician advice for problem alcohol drinkers. A randomized controlled trial in community-based primary care practices. Jama 1997, 277:1039-1045

172. Adams AK, Wermuth EO, McBride PE: Antioxidant vitamins and the prevention of coronary heart disease. Am Fam Physician 1999, 60:895-904

173. Keevil JG, Stein JH, McBride PE: Cardiovascular disease prevention. Prim Care 2002, 29:667-696

174. Stein JH, McBride PE: Screening and managing patients with lipoprotein (a) excess. Intern Med 1999, 20:9-21

175. Schauer JE, Stein JH, McBride PE: Atherosclerotic vascular disease prevention: Advances in risk factor management. Family Practice Recertification 1998, 20:13-35

176. Stein JH, McBride PE: Hyperhomocysteinemia and atherosclerotic vascular disease: pathophysiology, screening, and treatment. off. Arch Intern Med 1998, 158:1301-1306

177. Stein JH, McBride PE: Benefits of cholesterol screening and therapy for primary prevention of cardiovascular disease: a new paradigm. J Am Board Fam Pract 1998, 11:72-77

178. Fallest-Strobl PC, Koch DD, Stein JH, McBride PE: Homocysteine: a new risk factor for atherosclerosis. Am Fam Physician 1997, 56:1607-1612, 1615-1606

179. Chambless LE, Folsom AR, Sharrett AR, Sorlie P, Couper D, Szklo M, Nieto FJ: Coronary heart disease risk prediction in the Atherosclerosis Risk in Communities (ARIC) study. J Clin Epidemiol 2003, 56:880-890

180. Nieto FJ, Harrington DM, Redline S, Benjamin EJ, Robbins JA: Sleep apnea and markers of vascular endothelial function in a large community sample of older adults. Am J Respir Crit Care Med 2003

181. Ding J, Diez Roux AV, Nieto FJ, McNamara RL, Hetmanski JB, Taylor HA, Jr., Tyroler HA: Racial disparity in long-term mortality rate after hospitalization for myocardial infarction: the Atherosclerosis Risk in Communities study. Am Heart J 2003, 146:459-464

182. Quan SF, Griswold ME, Iber C, Nieto FJ, Rapoport DM, Redline S, Sanders M, Young T: Short-term variablility of respiration and sleep during unattended nonlaboratory polysomnogaphy--the Sleep Heart Health Study. Sleep 2002, 25:843-849

183. Leonard KE, Roberts LJ: The effects of alcohol on the marital interactions of aggressive and nonaggressive husbands and their wives. J Abnorm Psychol 1998, 107:602-615

184. Roberts LJ, Greenberg DR: Observational windows to intimacy processes in marriage. Understanding marriage: Developments in the study of couple interaction. Edited by Noller P, Feeny J. New York, Cambridge University Press, 2002, pp 118-149

185. Roberts LJ, Leonard KE: Gender differences and similarities in the alcohol and marriage relationship. Gender and alcohol: Individual and social perspectives. Edited by Wisnack S, Wilsnack R. New Brunswick, NJ, Rutgers Center of Alcohol Studies, 1997, pp 289-311

186. Roberts LJ, Linney KD: Alcohol problems and couples: Drinking in an intimate, relational context. The psychology of couples and illness. Edited by Schmaling K, Goldman Sher T. Washington, D.C, American Psychological Association, 2000, pp 269-310

187. Roberts LJ, McCrady BS: Alcohol problems in intimate relationships: Identification and intervention (NIH publication number 03-5284). Washington, D.C., U.S. Government Printing Office, 2003

188. Stein JH, Carlsson CM, Papcke-Benson K, Aeschlimann SE, Bodemer A, Carnes M, McBride PE: The effects of lipid-lowering and antioxidant vitamin therapies on flow-mediated vasodilation of the brachial artery in older adults with hypercholesterolemia. J Am Coll Cardiol 2001, 38:1806-1813

189. Stein JH, Fraizer MC, Aeschlimann SE, Nelson-Worel J, McBride PE, Douglas PS: Individualizing coronary risk assessment using carotid intima media thickness measurements to estimate vascular age, Clinical Cardiology, (in press)

190. Chuang ML, Douglas PS, Bisinov EA, Stein JH: Effect of cardiac cycle on ultrasound assessment of endothelial function. Vasc Med 2002, 7:103-108

191. Stein JH, Keevil JG, Wiebe DA, Aeschlimann S, Folts JD: Purple grape juice improves endothelial function and reduces the susceptibility of LDL cholesterol to oxidation in patients with coronary artery disease. Circulation 1999, 100:1050-1055

192. Stein JH, Klein MA, Bellehumeur JL, McBride PE, Wiebe DA, Otvos JD, Sosman JM: Use of human immunodeficiency virus-1 protease inhibitors is associated with atherogenic lipoprotein changes and endothelial dysfunction. Circulation 2001, 104:257-262

193. Hu FB, Stampfer MJ, Haffner SM, Solomon CG, Willett WC, Manson JE: Elevated risk of cardiovascular disease prior to clinical diagnosis of type 2 diabetes. Diabetes Care 2002, 25:1129-1134

194. Fung TT, Hu FB, Pereira MA, Liu S, Stampfer MJ, Colditz GA, Willett WC: Whole-grain intake and the risk of type 2 diabetes: a prospective study in men. Am J Clin Nutr 2002, 76:535-540

195. Osganian SK, Stampfer MJ, Rimm E, Spiegelman D, Manson JE, Willett WC: Dietary carotenoids and risk of coronary artery disease in women. Am J Clin Nutr 2003, 77:1390-1399

196. McCullough ML, Feskanich D, Stampfer MJ, Giovannucci EL, Rimm EB, Hu FB, Spiegelman D, Hunter DJ, Colditz GA, Willett WC: Diet quality and major chronic disease risk in men and women: moving toward improved dietary guidance. Am J Clin Nutr 2002, 76:1261-1271

197. Cho E, Manson JE, Stampfer MJ, Solomon CG, Colditz GA, Speizer FE, Willett WC, Hu FB: A prospective study of obesity and risk of coronary heart disease among diabetic women. Diabetes Care 2002, 25:1142-1148

198. Wu K, Willett WC, Chan JM, Fuchs CS, Colditz GA, Rimm EB, Giovannucci EL: A prospective study on supplemental vitamin e intake and risk of colon cancer in women and men. Cancer Epidemiol Biomarkers Prev 2002, 11:1298-1304

199. Al-Delaimy WK, Rimm E, Willett WC, Stampfer MJ, Hu FB: A prospective study of calcium intake from diet and supplements and risk of ischemic heart disease among men. Am J Clin Nutr 2003, 77:814-818

200. Michaud DS, Giovannucci E, Willett WC, Colditz GA, Fuchs CS: Dietary meat, dairy products, fat, and cholesterol and pancreatic cancer risk in a prospective study. Am J Epidemiol 2003, 157:1115-1125

201. Feskanich D, Willett WC, Colditz GA: Calcium, vitamin D, milk consumption, and hip fractures: a prospective study among postmenopausal women. Am J Clin Nutr 2003, 77:504-511

202. Baker T, Hatsukami D, Lerman C, O'Malley S, Shields A, Fiore M: Transdisciplinary science applied to the evaluation of treatments for tobacco use. Nicotine & Tobacco Research 2003, 5:S89-99

203. Fiore MC, Bailey WC, Cohen SJ: Treating tobacco use and dependence: Clinical Practice Guideline. Rockville, MD, U.S. Department of Health and Human Services, U.S. Public Health Service, 2000

204. McKenzie M, Tulsky JP, Long HL, Chesney M, Moss A: Tracking and follow-up of marginalized populations: a review. J Health Care Poor Underserved 1999, 10:409-429

205. Jorenby DE, Fiore MC: The Agency for Health Care Policy and Research smoking cessation clinical practice guideline: basics and beyond. Prim Care 1999, 26:513-528

206. Bohadana A, Nilsson F, Rasmussen T, Martinet Y: Nicotine inhaler and nicotine patch as a combination therapy for smoking cessation: a randomized, double-blind, placebo-controlled trial. Arch Intern Med 2000, 160:3128-3134

207. Blondal T, Gudmundsson LJ, Olafsdottir I, Gustavsson G, Westin A: Nicotine nasal spray with nicotine patch for smoking cessation: randomised trial with six year follow up. Bmj 1999, 318:285-288

208. Tonnesen P, Paoletti P, Gustavsson G, Russell MA, Saracci R, Gulsvik A, Rijcken B, Sawe U: Higher dosage nicotine patches increase one-year smoking cessation rates: results from the European CEASE trial. Collaborative European Anti-Smoking Evaluation. European Respiratory Society. Eur Respir J 1999, 13:238-246

209. Daughton D, Susman J, Sitorius M, Belenky S, Millatmal T, Nowak R, Patil K, Rennard SI: Transdermal nicotine therapy and primary care. Importance of counseling, demographic, and participant selection factors on 1-year quit rates. The Nebraska Primary Practice Smoking Cessation Trial Group. Arch Fam Med 1998, 7:425-430

210. Killen JD, Fortmann SP, Davis L, Varady A: Nicotine patch and self-help video for cigarette smoking cessation. J Consult Clin Psychol 1997, 65:663-672

211. Richmond R, Zwar N: Review of bupropion for smoking cessation. Drug Alcohol Rev 2003, 22:203-220

212. Badawi MA, Eaton WW, Myllyluoma J, Weimer LG, Gallo J: Psychopathology and attrition in the Baltimore ECA 15-year follow-up 1981-1996. Soc Psychiatry Psychiatr Epidemiol 1999, 34:91-98

213. Deeg DJ, van Tilburg T, Smit JH, de Leeuw ED: Attrition in the Longitudinal Aging Study Amsterdam. The effect of differential inclusion in side studies. J Clin Epidemiol 2002, 55:319-328

214. Bjornson W, Rand C, Connett JE, Lindgren P, Nides M, Pope F, Buist AS, Hoppe-Ryan C, O'Hara P: Gender differences in smoking cessation after 3 years in the Lung Health Study. Am J Public Health 1995, 85:223-230

215. BootsMiller BJ, Ribisl KM, Mowbray CT, Davidson WS, Walton MA, Herman SE: Methods of ensuring high follow-up rates: lessons from a longitudinal study of dual diagnosed participants. Subst Use Misuse 1998, 33:2665-2685

216. Cotter RB, Burke, J.D., Loeber, R., & Navratil, J.L.: Innovative retention methods in longitudinal research: a case of the developmental trends study. Journal of Child and Family Studies 2002, 11:485-498

217. Claus RE, Kindleberger LR, Dugan MC: Predictors of attrition in a longitudinal study of substance abusers. J Psychoactive Drugs 2002, 34:69-74

218. Janson SL, Alioto ME, Boushey HA: Attrition and retention of ethnically diverse subjects in a multicenter randomized controlled research trial. Control Clin Trials 2001, 22:236S-243S

219. Navaratil JL, Green, S. M., Loeber, R., & Lahey, B. B.: Minimizing subject loss in a longitudinal study of deviant behavior. Journal of Child and Family Studies 1994, 3:89-106

220. Stouthamer-Loeber M, Van Kammen, W., Loeber, R.: The nuts and bolts of implementing large-scale longitudinal studies. Violence Vict 1992, 7:63-78

221. Siddiqui O, Flay BR, Hu FB: Factors affecting attrition in a longitudinal smoking prevention study. Prev Med 1996, 25:554-560

222. Prinz RJ, Smith EP, Dumas JE, Laughlin JE, White DW, Barron R: Recruitment and retention of participants in prevention trials involving family-based interventions. Am J Prev Med 2001, 20:31-37

223. Li Q, Hyland, A., Bauer, J., Giovino, G., & Cummings, M.: Long-term predictors of indicators of smoking cessation and relapse. Presented at the Society for Research on Nicotine and Tobacco, New Orleans, February, 2003 2003

224. Nordstrom BL, Kinnunen T, Utman CH, Krall EA, Vokonas PS, Garvey AJ: Predictors of continued smoking over 25 years of follow-up in the normative aging study. American Journal of Public Health 2000, 90:404-406

225. Touboul PJ, Prati P, Scarabin PY, Adrai V, Thibout E, Ducimetiere P: Use of monitoring software to improve the measurement of carotid wall thickness by B-mode imaging. J Hypertens Suppl 1992, 10:S37-41

226. Riley W, Barnes, R., Bond, M., Evans G., Chambless, L., Heiss, G.: High-resolution B-mode ultrasound reading methods in the Atherosclerosis Risk in Communities (ARIC) cohort. The ARIC Study Group. J Neuroimaging 1991, 1:168-172

227. Wendelhag I, Liang Q, Gustavsson T, Wikstrand J: A new automated computerized analyzing system simplifies readings and reduces the variability in ultrasound measurement of intima-media thickness. Stroke 1997, 28:2195-2200

228. Selzer RH, Hodis HN, Kwong-Fu H, Mack WJ, Lee PL, Liu CR, Liu CH: Evaluation of computerized edge tracking for quantifying intima-media thickness of the common carotid artery from B-mode ultrasound images. Atherosclerosis 1994, 111:1-11

229. Celermajer DS, Sorensen KE, Gooch VM, Spiegelhalter DJ, Miller OI, Sullivan ID, Lloyd JK, Deanfield JE: Non-invasive detection of endothelial dysfunction in children and adults at risk of atherosclerosis. Lancet 1992, 340:1111-1115

230. Corretti MC, Plotnick GD, Vogel RA: Technical aspects of evaluating brachial artery vasodilatation using high-frequency ultrasound. Am J Physiol 1995, 268:H1397-1404

231. Celermajer DS, Sorensen KE, Georgakopoulos D, Bull C, Thomas O, Robinson J, Deanfield JE: Cigarette smoking is associated with dose-related and potentially reversible impairment of endothelium-dependent dilation in healthy young adults. Circulation 1993, 88:2149-2155

232. Heitzer T, Yla-Herttuala S, Luoma J, Kurz S, Munzel T, Just H, Olschewski M, Drexler H: Cigarette smoking potentiates endothelial dysfunction of forearm resistance vessels in patients with hypercholesterolemia. Role of oxidized LDL. Circulation 1996, 93:1346-1353

233. Corretti MC, Anderson TJ, Benjamin EJ, Celermajer D, Charbonneau F, Creager MA, Deanfield J, Drexler H, Gerhard-Herman M, Herrington D, Vallance P, Vita J, Vogel R: Guidelines for the ultrasound assessment of endothelial-dependent flow-mediated vasodilation of the brachial artery: a report of the International Brachial Artery Reactivity Task Force. J Am Coll Cardiol 2002, 39:257-265

234. Celermajer DS, Adams MR, Clarkson P, Robinson J, McCredie R, Donald A, Deanfield JE: Passive smoking and impaired endothelium-dependent arterial dilatation in healthy young adults. New England Journal of Medicine 1996, 334:150-154

235. Celermajer DS: Endothelial dysfunction: does it matter? Is it reversible? J Am Coll Cardiol 1997, 30:325-333

236. Mano T, Masuyama T, Yamamoto K, Naito J, Kondo H, Nagano R, Tanouchi J, Hori M, Inoue M, Kamada T: Endothelial dysfunction in the early stage of atherosclerosis precedes appearance of intimal lesions assessable with intravascular ultrasound. Am Heart J 1996, 131:231-238

237. Suwaidi JA, Hamasaki S, Higano ST, Nishimura RA, Holmes DR, Jr., Lerman A: Long-term follow-up of patients with mild coronary artery disease and endothelial dysfunction. Circulation 2000, 101:948-954

238. Neunteufl T, Heher S, Katzenschlager R, Wolfl G, Kostner K, Maurer G, Weidinger F: Late prognostic value of flow-mediated dilation in the brachial artery of patients with chest pain. Am J Cardiol 2000, 86:207-210

239. Vogel RA: Measurement of endothelial function by brachial artery flow-mediated vasodilation. Am J Cardiol 2001, 88:31E-34E

240. Mannion TC, Vita JA, Keaney JF, Jr., Benjamin EJ, Hunter L, Polak JF: Non-invasive assessment of brachial artery endothelial vasomotor function: the effect of cuff position on level of discomfort and vasomotor responses. Vasc Med 1998, 3:263-267

241. Herrington DM, Werbel BL, Riley WA, Pusser BE, Morgan TM: Individual and combined effects of estrogen/progestin therapy and lovastatin on lipids and flow-mediated vasodilation in postmenopausal women with coronary artery disease. J Am Coll Cardiol 1999, 33:2030-2037

242. Uehata A, Lieberman EH, Gerhard MD, Anderson TJ, Ganz P, Polak JF, Creager MA, Yeung AC: Noninvasive assessment of endothelium-dependent flow-mediated dilation of the brachial artery. Vasc Med 1997, 2:87-92

243. Sorensen KE, Celermajer DS, Spiegelhalter DJ, Georgakopoulos D, Robinson J, Thomas O, Deanfield JE: Non-invasive measurement of human endothelium dependent arterial responses: Accuracy and reproducibility. Br Heart J 1995, 74:247-253

244. Otvos JD: Measurement of lipoprotein subclass profiles by nuclear magnetic resonance spectroscopy. Handbook of lipoprotein testing, 2nd edition. Edited by Rifai N, Warnick G, Dominiczak MH. Washington, D.C, AACC Press, pp 609-623

245. Otvos JD: Why cholesterol measurements may be misleading about lipoprotein levels and cardiovascular disease risk - clinical implication of lipoprotein quantification using NMR spectroscopy. Journal of Laboratory Medicine 2002, 26:544-550

246. Rosenson RS, Otvos JD, Freedman DS: Relations of lipoprotein subclass levels and low-density lipoprotein size to progression of coronary artery disease in the Pravastatin Limitation of Atherosclerosis in the Coronary Arteries (PLAC-I) trial. Am J Cardiol 2002, 90:89-94

247. Otvos JD, Jeyarajah EJ, Cromwell WC: Measurement issues related to lipoprotein heterogeneity. Am J Cardiol 2002, 90:22i-29i

248. Kuller L, Arnold A, Tracy R, Otvos J, Burke G, Psaty B, Siscovick D, Freedman DS, Kronmal R: Nuclear magnetic resonance spectroscopy of lipoproteins and risk of coronary heart disease in the cardiovascular health study. Arterioscler Thromb Vasc Biol 2002, 22:1175-1180

249. Blake GJ, Otvos JD, Rifai N, Ridker PM: Low-density lipoprotein particle concentration and size as determined by nuclear magnetic resonance spectroscopy as predictors of cardiovascular disease in women. Circulation 2002, 106:1930-1937

250. Mackey RH, Kuller LH, Sutton-Tyrrell K, Evans RW, Holubkov R, Matthews KA: Lipoprotein subclasses and coronary artery calcium in postmenopausal women from the healthy women study. Am J Cardiol 2002, 90:71i-76i

251. Craig CL, Marshall AL, Sjostrom M, Bauman AE, Booth ML, Ainsworth BE, Pratt M, Ekelund U, Yngve A, Sallis JF, Oja P: International physical activity questionnaire: 12-country reliability and validity. Med Sci Sports Exerc 2003, 35:1381-1395

252. Bassett Jr. DR, Strath, SJ.: Use of pedometers to assess physical activity. Physical activity assessments for health-related research. Edited by Welk G. Champaign, IL, Human Kinetics Publishers, Inc., 2002

253. Pate RR, Pratt M, Blair SN, Haskell WL, Macera CA, Bouchard C, Buchner D, Ettinger W, Heath GW, King AC, et al.: Physical activity and public health. A recommendation from the Centers for Disease Control and Prevention and the American College of Sports Medicine. JAMA 1995, 273:402-407

254. Schneider PL, Crouter SE, Lukajic O, Bassett DR, Jr.: Accuracy and reliability of 10 pedometers for measuring steps over a 400-m walk. Med Sci Sports Exerc 2003, 35:1779-1784

255. Tudor-Locke CE, Myers AM: Methodological considerations for researchers and practitioners using pedometers to measure physical (ambulatory) activity. Res Q Exerc Sport 2001, 72:1-12

256. Balke B, Ware RW: An experimental study of physical fitness of Air Force personnel. U S Armed Forces Medical Journal 1959, 10:875-888

257. Rimm EB, Giovannucci EL, Stampfer MJ, Colditz GA, Litin LB, Willett WC: Reproducibility and validity of an expanded self-administered semiquantitative food frequency questionnaire among male health professionals. Am J Epidemiol 1992, 135:1114-1126; discussion 1127-1136

258. Willett WC, Colditz GA: Approaches for conducting large cohort studies. Epidemiol Rev 1998, 20:91-99

259. Willett WC: Nutritional epidemiology, 2nd edition. New York, Oxford University Press, 1998

260. Stryker WS, Kaplan LA, Stein EA, Stampfer MJ, Sober A, Willett WC: The relation of diet, cigarette smoking, and alcohol consumption to plasma beta-carotene and alpha-tocopherol levels. Am J Epidemiol 1988, 127:283-296

261. Ascherio A, Stampfer MJ, Colditz GA, Rimm EB, Litin L, Willett WC: Correlations of vitamin A and E intakes with the plasma concentrations of carotenoids and tocopherols among American men and women. J Nutr 1992, 122:1792-1801

262. Giovannucci E, Stampfer MJ, Colditz GA, Rimm EB, Trichopoulos D, Rosner BA, Speizer FE, Willett WC: Folate, methionine, and alcohol intake and risk of colorectal adenoma. J Natl Cancer Inst 1993, 85:875-884

263. Hunter DJ, Rimm EB, Sacks FM, Stampfer MJ, Colditz GA, Litin LB, Willett WC: Comparison of measures of fatty acid intake by subcutaneous fat aspirate, food frequency questionnaire, and diet records in a free-living population of US men. Am J Epidemiol 1992, 135:418-427

264. London SJ, Sacks FM, Caesar J, Stampfer MJ, Siguel E, Willett WC: Fatty acid composition of subcutaneous adipose tissue and diet in postmenopausal US women. Am J Clin Nutr 1991, 54:340-345

265. Rifas-Shiman SL, Willett WC, Lobb R, Kotch J, Dart C, Gillman MW: PrimeScreen, a brief dietary screening tool: reproducibility and comparability with both a longer food frequency questionnaire and biomarkers. Public Health Nutr 2001, 4:249-254

266. Robins LN, Regier DA: Psychiatric disorders in America. New York, MacMillan, 1991

267. Kessler RC, McGonagle KA, Zhao S, Nelson CB, Hughes M, Eshleman S, Wittchen HU, Kendler KS: Lifetime and 12-month prevalence of DSM-III-R psychiatric disorders in the United States. Results from the National Comorbidity Survey. Archives of General Psychiatry 1994, 51:8-19

268. Wittchen HU, Robins LN, Cottler LB, Sartorius N, Burke JD, Regier D: Cross-cultural feasibility, reliability and sources of variance of the Composite International Diagnostic Interview (CIDI). The Multicentre WHO/ADAMHA Field Trials. Br J Psychiatry 1991, 159:645-653, 658

269. Wittchen HU: Reliability and validity studies of the WHO--Composite International Diagnostic Interview (CIDI): a critical review. J Psychiatr Res 1994, 28:57-84

270. Holden C: Mental health. Global survey examines impact of depression. Science 2000, 288:39-40

271. Kessler RC, Ustun TB: The World Health Organization World Mental Health 2000 Initiative. Hospital Management International 2000:195-196

272. Girolamo G, Bassi M: Community surveys of mental disorders: Recent achievements and works in progress. Current Opinion in Psychiatry 2003, 16:403-411

273. Kessler RC, Berglund P, Demler O, Jin R, Koretz D, Merikangas KR, Rush AJ, Walters EE, Wang PS: The epidemiology of major depressive disorder: results from the National Comorbidity Survey Replication (NCS-R). Jama 2003, 289:3095-3105

274. Watson D, Clark LA, Tellegen A: Development and validation of brief measures of positive and negative affect: the PANAS scales. J Pers Soc Psychol 1988, 54:1063-1070

275. Frisch MB, Cornell J, Villanueva M, Retzlaff PJ: Clinical validation of the Quality of Life Inventory: A measure of life satisfaction for use in treatment planning and outcome assessments. Psychological Assessment 1992, 4:92-101

276. Alloy L, Abramson L, Metalsky G, Harlages S: The Depression Proneness Inventory: A stress-responsive measure of vulnerability to depression. (unpublished manuscript) 1990

277. Hosmer DW, Lemeshow S: Applied Logistic Regression, Second Edition. New York, John Wiley & Sons, Inc., 2000

278. Allison PD: Missing data techniques for structural equation modeling. J Abnorm Psychol 2003, 112:545-557

279. Yuan KH, Chan W, Bentler PM: Robust transformation with applications to structural equation modelling. Br J Math Stat Psychol 2000, 53 ( Pt 1):31-50

280. Cohen J, Cohen P, West SG, Aiken LS: Applied multiple regression/correlation analysis in the behavioral sciences (3rd ed.). Malwah, NJ, Lawrence Erlbaum Associates, 2003

281. Rogosa D: Myths about longitudinal research. Methodological issues in aging research. Edited by K.W. S, R.T. Campbell, W. Meredith & S.C. Rawlings. San Diego, Springer, 1988, pp 171-210

282. Maris E: Covariance adjustment versus gain scores-revisited. Psychological Methods 1998, 3:309-327

283. Gariepy J, Denarie N, Chironi G, Salomon J, Levenson J, Simon A: Gender difference in the influence of smoking on arterial wall thickness. Atherosclerosis 2000, 153:139-145

284. Mowbray PI, Lee AJ, Fowkes GR, Allan PL: Cardiovascular risk factors for early carotid atherosclerosis in the general population: the Edinburgh Artery Study. J Cardiovasc Risk 1997, 4:357-362

285. Aber MS, McArdle JJ: Latent growth curve approaches to modeling the development of competence. Criteria for competence. Edited by Chandler M, Chapman M. Newbury Park, CA, Sage, 1991, pp 231-258

286. Curran PJ, Hussong AM: The use of latent trajectory models in psychopathology research. J Abnorm Psychol 2003, 112:526-544

287. Tomarken AJ, Baker TB: Introduction to the special section on structural equation modeling. J Abnorm Psychol 2003, 112:523-525

288. Bots ML, Evans GW, Riley WA, Grobbee DE: Carotid intima-media thickness measurements in intervention studies: design options, progression rates, and sample size considerations: a point of view. Stroke 2003, 34:2985-2994

289. Eaton WW: Studying the natural history of psychopathology. Textbook in psychiatric epidemiology (2nd ed.). Edited by Tsuang MT, Tohen A. New York, Wiley-Liss, 2002, pp 215-238

290. De Graaf R, Bijl RV, Ten Have M, Beekman AT, Vollebergh WA: Rapid onset of comorbidity of common mental disorders: findings from the Netherlands Mental Health Survey and Incidence Study (NEMESIS). Acta Psychiatr Scand 2004, 109:55-63

291. Kessler RC: Epidemiology of depression. Handbook of depression. New York, Guilford, 2002, pp 23-42

292. Breslau N, Kilbey M, Andreski P: Nicotine dependence, major depression, and anxiety in young adults. Archives of General Psychiatry 1991, 48:1069-1074

293. Breslau N, Johnson EO: Predicting smoking cessation and major depression in nicotine-dependent smokers. American Journal of Public Health 2000, 90:1122-1128

**H. Consortium/Contractual Arrangements** – N/A

**I. Consultants**

University of Wisconsin budgetary procedures do not permit co-investigators who are not UW employees to be listed in the personnel section of the budget. Hence, co-investigators who are not UW employees are listed as consultants.

**J. Letters of Support/Commitment**

See attached letters.
